# A subnational affordability assessment of nutritious foods for complementary feeding in Kenya

**DOI:** 10.1111/mcn.13373

**Published:** 2022-06-06

**Authors:** Theresa Ryckman, Patrick Codjia, Stella Nordhagen, Caroline Arimi, Veronica Kirogo, Laura Kiige, Penjani Kamudoni, Ty Beal

**Affiliations:** ^1^ Department of Epidemiology Johns Hopkins Bloomberg School of Public Health Baltimore Maryland USA; ^2^ UNICEF Kenya Country Office Nairobi Kenya; ^3^ Global Alliance for Improved Nutrition (GAIN) Geneva Switzerland; ^4^ Division of Nutrition and Dietetics Ministry of Health Nairobi Kenya; ^5^ Global Alliance for Improved Nutrition (GAIN) Washington DC USA

**Keywords:** affordability, child undernutrition, complementary feeding, dietary diversity, Kenya, micronutrients, price

## Abstract

Complementary feeding among children aged 6–23 months is a key determinant of micronutrient deficiencies and childhood stunting, the burdens of which remain high in Kenya. This study examines the affordability of complementary foods to increase young children's nutrient consumption across eight provinces in Kenya. We combined data from household surveys, food composition tables and published sources to estimate the cost of portion sizes that could meet half of the children's daily iron, vitamin A, calcium, zinc, folate, vitamin B_12_ and protein requirements from complementary feeding. These costs were compared to current household food expenditures. The selection of foods and price and expenditure data were stratified by province. Our analysis indicates that vitamin A, vitamin B_12_ and folate are affordable to most households in Kenya via liver, beans and in some provinces, orange‐fleshed fruits and vegetables, avocado and small dried fish. Calcium, animal‐source protein, zinc and iron were less affordable and there was more provincial variation. In some provinces, small dried fish were an affordable source of calcium, protein and zinc. In others (North Eastern, Central, Eastern, parts of Rift Valley and Coast), small dried fish were not commonly consumed and other foods were less affordable. Future research should consider interventions aimed at reducing prices, increasing availability and changing behaviours related to these foods. Solutions such as supplementation and fortification may be needed for iron and zinc in some locations. Food affordability presented the greatest barriers in North Eastern province, which had lower dietary diversity and may require additional targeted interventions.

## Key messages

- Small dried fish, liver, milk, dark green leafy vegetables, beans and eggs are the most affordable food sources of nutrients lacking during the complementary feeding period in Kenya.
- Unaffordability prevents adequate consumption of iron and zinc among young children. Fortification and supplementation may be needed.
- Availability of small dried fish—which is lowest in Central, Eastern, North Eastern and parts of Rift Valley and Coast provinces—is a key determinant of calcium, animal‐source protein and zinc affordability.
- Gaps in vitamin A, vitamin B_12_ and folate consumption among young children are likely rooted in other causes, such as inconsistent availability or demand‐side barriers.

## Graphical abstract

This study examines the affordability of complementary foods to increase young children's nutrient consumption across eight provinces in Kenya. Vitamin A, vitamin B_12_ and folate are affordable to most households in Kenya via liver, beans and in some provinces, orange‐fleshed fruits and vegetables, avocado and small dried fish, while calcium, animal‐source protein, zinc and iron are less affordable with more provincial variation (depending in large part on small dried fish availability). Food affordability presented the greatest barriers in North Eastern province, which had lower dietary diversity and may require additional targeted interventions.

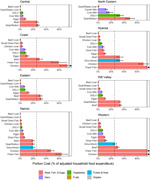

## 1 INTRODUCTION

Under‐five stunting is strongly linked to increased risk of infection, chronic conditions later in life, educational and developmental consequences and lower lifetime earning potential. Most childhood growth faltering occurs during the complementary feeding period: between 6 and 23 months of life when breast milk is no longer solely sufficient to meet children's nutritional needs. Kenya is home to an estimated 1.8 million stunted children. 26% of children under five are stunted and 78% of children aged 6–23 months are not fed a minimally acceptable diet (Kenya National Bureau of Statistics et al., 2015). A recent assessment of nutrient gaps in Kenya found that many children in the complementary feeding period are unlikely to consume adequate quantities of iron, vitamin A, calcium, zinc, folate and vitamin B_12_, putting them at increased risk of both micronutrient deficiencies and growth faltering (Global Alliance for Improved Nutrition & United Nations Children's Fund, 2021).

Kenya is home to ethnic, cultural and agroecological diversity, which affects food availability and preferences. There is substantial subnational variation in nutritional outcomes. Although Kenya is considered to have a high prevalence of stunting nationally (26%), stunting prevalence is very high (≥30%) in 8 of the 47 counties and moderate (10%–20%) in 10 others (de Onis et al., 2019; Kenya National Bureau of Statistics et al., 2015). In 2010 in a process known as devolution, Kenya enacted a new constitution that decentralized governance and policymaking, including the implementation of many health and nutrition programs, to the level of its 47 counties. Designing appropriate subnational nutrition programming thus requires information on how contributors to malnutrition manifest differently across the country's diverse subnational areas.

Other research has explored sociocultural drivers of complementary feeding practices (Kimiywe et al., 2022). Previous research has also highlighted unaffordability as a major barrier to the consumption of several micronutrients among children of complementary feeding age in other countries in Eastern and Southern Africa (Ryckman, Beal, Nordhagen, Chimanya, et al., 2021). However, the extent to which unaffordability drives suspected complementary feeding nutrient deficiencies and which nutrients and foods are most affordable in Kenya is largely unknown. Moreover, there is also limited research on the affordability of specific nutrients and foods sub‐nationally within countries, including Kenya.

In this study, we analyze household survey data on food consumption in Kenya to assess food and nutrient affordability. We build upon a previously developed approach to assessing affordability by conducting analysis at the provincial level, with the objective of elucidating subnational variation in food consumption, prices and affordability. Our findings can be used to inform efforts to increase consumption of affordable foods and identify locations, foods and nutrients for which affordability barriers exist and other programmatic actions may be needed.

## 2 METHODS

### 2.1 Overview

The methods used to conduct this analysis are consistent with previous analyses (Ryckman, Beal, Nordhagen, Chimanya, et al., 2021; Ryckman, Beal, Nordhagen, Murira, et al., 2021) and are described more in Box 1. There were three major components: (1) identifying foods that are commonly consumed by households and could supply one or more nutrients of interest, (2) calculating quantities (portion sizes) of these foods that could provide half of the daily requirements for each nutrient and an average of one‐third of requirements across six micronutrients from complementary feeding and (3) assessing the costs of these quantities and comparing them to current household food expenditures, adjusted for household composition. Previous analyses using this method were conducted by country (Ryckman, Beal, Nordhagen, Chimanya, et al., 2021; Ryckman, Beal, Nordhagen, Murira, et al., 2021); we extended these analyses by conducting both components 1 and 3 nationally and separately for each province. Before the 2010 devolution to counties, provinces were the highest subnational administrative unit in Kenya (Table 1).

Box 1 **Analytical steps in assessing food and nutrient affordability**

**Table:** 

**Step**	**Source(s)**	**Which analysis?**	**Rationale**
**Component 1. Food and nutrient identification**			
Identify foods consumed by at least 10% of households in each province in Kenya	KIHBS (Kenya National Bureau of Statistics, 2018)	Single and Joint	Goal is to identify foods that will be locally available and acceptable.
Identify nutrients with evidence of complementary feeding gaps in Kenya	Global Alliance for Improved Nutrition and United Nations Children's Fund (2021)	Single and Joint	A comprehensive assessment was taken to identify nutrients with evidence of gaps. See Global Alliance for Improved Nutrition and United Nations Children's Fund (2021) for more details.
**Component 2. Portion size calculation**			
Obtain data on each food's density of identified micronutrients from component 1 (iron, vitamin A, calcium, zinc, folate and vitamin B_12_), refuse and cooking yield.	Bognar (2002); Kabahenda et al. (2011); Korkalo et al. (2011); Lukmanji et al. (2008); Mwai et al. (2018); Nyirenda et al. (2009); Roseland et al. (2014); Stadlymayr et al. (2012); Steiner‐Asiedu et al. (1993); U.S. Department of Agriculture Agricultural Research Service (2019). See Supporting Information: Appendix for details	Single and Joint	We used the Kenya FCT whenever possible because it provides local data and supplemented with other FCTs when needed. We included data on refuse and cooking yield because many foods are purchased with refuse (e.g. bones, peels) that are not consumed and nutrient density data are often for the cooked form of food, but food weight changes from cooking.
For animal‐source foods only, obtain data on each food's protein density	Bognar (2002); Kabahenda et al. (2011); Korkalo et al. (2011); Lukmanji et al. (2008); Mwai et al. (2018); Nyirenda et al. (2009); Roseland et al. (2014); Stadlymayr et al. (2012); Steiner‐Asiedu et al. (1993); U.S. Department of Agriculture Agricultural Research Service (2019)	Single only	Plant‐based protein sources generally do not provide complete amino acids.
Calculate half of a 6‐ to 23‐month‐old child's daily requirements for protein and micronutrients from complementary foods	Dewey (2001); World Health Organization and Food and Agriculture Organization of the United Nations (2004)	Single and Joint	Half was selected because children consume nutrients through a variety of foods in the diet, not just one food or food group. Nutrient requirements from complementary foods exclude the suggested portion of requirements from breast milk.
Calculate adjusted daily requirements for iron and zinc from plant‐source foods	World Health Organization and Food and Agriculture Organization of the United Nations (2004)	Single and Joint	Absorption of iron and zinc from plant‐source foods is lower than from animal‐source foods.
Match daily nutrient requirements calculations with nutrient density data to calculate a portion size for each food‐nutrient combination	Analytical step	Single only	Portion sizes depend on foods' nutrient densities and on nutrient requirements.
Calculate the portion size for which a food provides an average of one‐third of joint requirements for the micronutrients from component 1 (in combination)	Analytical step—see Supporting Information: Appendix page 5 for details	Joint only	Most foods provide multiple nutrients, which are not accounted for in the single nutrient analysis. One‐third was selected as a benchmark to determine which foods to supply reasonably high quantities of multiple micronutrients.
Exclude foods with portion sizes >100 g for solid foods (>80 g for milk)	Analytical step	Single and Joint	Children will not be capable of consuming unreasonably large portions of a food.
**Component 3: Affordability assessment**			
Identify local prices for each food	KIHBS (Kenya National Bureau of Statistics, 2018)	Single and Joint	Food prices are matched to each household; prices vary both between and within provinces.
Identify current total household food expenditures per AEQ	KIHBS (Kenya National Bureau of Statistics, 2018)	Single and Joint	Expenditures vary by household. AEQs allow the analysis to incorporate household size and composition.
Calculate the cost of each food‐nutrient portion size for each household, based on the price data and food‐nutrient portion sizes	Analytical step	Single only	The cost of a quantity of a food equals its unit price multiplied by the quantity.
Calculate the cost of each portion as a percentage of current food expenditures per AEQ for each household. Compare this percentage to a 7.6% affordability threshold.	Analytical step	Single only	10% is a benchmark used in other analyses. We adjusted it for the lower average expenditures of households with young children in the KIHBS, compared to all households.
Calculate the cost of meeting one‐third of joint nutrient requirements, based on the price data and food portion sizes	Analytical step	Joint only	The cost of obtaining a quantity of a food equals its unit price multiplied by the quantity.
Calculate the cost of each portion as a percentage of current food expenditures per AEQ for each household. Compare this to a 25.2% threshold	Analytical step	Joint only	33.3% corresponds to meeting one‐third of joint requirements and is higher than the single nutrient thresholds because multiple requirements are met simultaneously. We adjusted the 33.3% threshold down to 25.2% to account for the lower average expenditures of households with young children, compared to all households.
**Component 4. Sensitivity analysis**			
Identify current household consumption of each food	KIHBS	Single only	This analysis accounts for the fact that households are often already consuming a food and thus may not have to purchase the full portion size. It is left as a sensitivity analysis because the KIHBS lacks data on which household members consume a food.
Calculate adjusted portion sizes (specific to each household) by subtracting out current consumption	Analytical step	Single only	
Recalculate portion cost as a share of current food expenditures, using the adjusted portion sizes	Analytical step	Single only	
Assess sensitivity of results to uncertainty in nutrient densities	FCTs/Analytical step	Single only	This analysis explores the extent to which results rely on specific nutrient density values, some of which are uncertain or vary across settings.

Abbreviations: AEQ, adult equivalent; FCT, food composition table; KIHBS, Kenya Integrated Household Budget Survey.

**Table 1:** Kenya provinces and counties.

Province	Counties
Central	Kiambu, Kirinyaga, Murang'a, Nyandarua, Nyeri
Coast	Kilifi, Kwale, Lamu, Mombasa, Taita‐Taveta, Tana River
Eastern	Embu, Isiolo, Kitui, Machakos, Makueni, Marsabit, Meru, Tharaka‐Nithi
Nairobi	Nairobi City
North Eastern	Garissa, Mandera, Wajir
Nyanza	Homa Bay, Kisii, Kisumu, Migori, Nyamira, Siaya
Rift Valley	Baringo, Bomet, Elgeyo‐Marakwet, Kajiado, Kericho, Laikipia, Nakuru, Nandi, Narok, Samburu, Trans Nzoia, Turkana, Uasin Gishu, West Pokot
Western	Bungoma, Busia, Kakamega, Vihiga

### 2.2 Data

To identify foods and compare their costs to household food expenditure, we made use of data from the 2015–16 Kenya Integrated Household Budget Survey (KIHBS) (Kenya National Bureau of Statistics, 2018), a nationally representative household survey designed to obtain data on a range of socioeconomic indicators. The KIHBS contains data on the quantities of foods that households consumed over a 7‐day period. Food expenditures, defined as the monetary value of food consumed, were captured based on the monetary amount households spent on food purchases and the value of foods consumed from home production, gifts and other sources. Data on local market prices (i.e., prices paid by consumers) were also collected. 21,744 households with complete information on food expenditures were interviewed from September 2015 to August 2016. The survey is representative at the national, provincial and county levels. However, because of the small sample sizes of food price observations at the county level and the initial observation that food consumption patterns were generally similar across most counties in a province, the analysis was run at the national and provincial levels.

Selection of data on nutrient requirements from complementary foods for children aged 6–23 months followed previously published approaches (Supporting Information: Appendix Table 1) (Dewey, 2001; Food and Nutrition Board Institute of Medicine of the National Academies, 2005; Institute of Medicine US Committee to Review Dietary Reference Intakes for Vitamin D and Calcium, 2011; Institute of Medicine US Panel on Micronutrients, 2001; Institute of Medicine US Standing Committee on the Scientific Evaluation of Dietary Reference Intakes and its Panel on Folate Other B Vitamins and Choline, 1998; Ryckman, Beal, Nordhagen, Chimanya, et al., 2021; World Health Organization & Food and Agriculture Organization of the United Nations, 2004). These requirements assume a portion of overall nutrient needs will be met through breast milk or formula and the remainder will need to come from complementary foods. The lower bioavailability of plant‐source foods relative to animal‐source foods was incorporated for zinc and iron (World Health Organization & Food and Agriculture Organization of the United Nations, 2004). Data on nutrient densities came from Kenya food composition tables and published sources (Bognar, 2002; Kabahenda et al., 2011; Korkalo et al., 2011; Lukmanji et al., 2008; Mwai et al., 2018; Nyirenda et al., 2009; Roseland et al., 2014; Stadlymayr et al., 2012; Steiner‐Asiedu et al., 1993; U.S. Department of Agriculture Agricultural Research Service, 2019). We used median nutrient densities across all sources and assessed alternative nutrient densities for a selection of foods in the sensitivity analysis (Supporting Information: Appendix Tables 2–4). In addition to the six micronutrients with evidence of complementary feeding gaps (iron, vitamin A, calcium, zinc, folate, vitamin B_12_), we also assessed protein, focusing on animal sources of protein because plant‐based sources of protein are generally not complete in essential amino acids critical for child growth and development (Semba et al., 2016).

### 2.3 Statistical analysis

To target foods that are likely locally available and acceptable, we selected foods that were consumed by at least 10% of households in a province (based on KIHBS data). In the single nutrient analysis, we calculated portion sizes of these foods that could meet half of the daily nutrient requirements for each nutrient (Supporting Information: Appendix page 1 and Table 5). Half of the daily needs was chosen because nutrient requirements are generally met through a combination of different foods. We excluded foods with unreasonably large portion sizes for a young child. Foods and portion sizes therefore vary by both province and nutrient. Consumption and price data were not available on specific cuts of meat, but due to the high nutrient density of liver as compared to flesh meat, any time we included the meat of an animal (e.g., beef, chicken) for a province, we also included the liver of that animal (e.g., beef liver, chicken liver) in our analysis as a distinct food.

We multiplied the portion sizes by local prices from the KIHBS to calculate the cost of purchasing each portion size and divided these costs by adjusted household food expenditures to calculate the portion of current household resources for food that would need to be shifted to purchase a given food. Household food expenditures were adjusted for household size and composition based on the ‘adult equivalents’ living in the household, allowing us to account for the relative caloric requirements of household members (Claro et al., 2010; Coates et al., 2017).

Foods provide several nutrients and in many cases, the portion size of a food meeting half of the daily needs for one micronutrient would also provide a substantial quantity of another nutrient (Beal et al., 2021). In addition to assessing the affordability of foods for fulfilling a single nutrient requirement in isolation, we also assessed foods' joint micronutrient compositions. In this multiple nutrient analysis, the average share of micronutrient requirements measures the extent to which a food contributes meaningfully to the requirements for all six micronutrients (Supporting Information: Appendix Figures 1–3 and page 6). We calculated the cost, as a share of adjusted household food expenditure, of portion sizes that could meet an average of one‐third of joint micronutrient requirements, excluding foods with daily portion sizes greater than 100 g. One‐third corresponds to a portion fulfilling 100% of requirements for two of the six micronutrients, one‐third of requirements for each of the six micronutrients or something in between.

The calculations described thus far—cost of a portion size as a share of adjusted household food expenditures—allow for the comparison of relative affordability across nutrients and foods. We also assessed the absolute affordability of foods and nutrients by comparing the cost of foods per adjusted household food expenditure against affordability thresholds of 7.6% and 25.2%. Thresholds were selected based on previous analyses (Ryckman, Beal, Nordhagen, Chimanya, et al., 2021; Ryckman, Beal, Nordhagen, Murira, et al., 2021) and then adjusted downwards to account for the lower average food expenditures among households with children with young children observed in the KIHBS. More details are available in the Supporting Information: Appendix (page 10).

The base case analysis does not incorporate current consumption of a food, due to a lack of evidence on how consumption is spread across household members. In sensitivity analysis, we estimated the net cost of a food by subtracting out current expenditure on that food (adjusted for household size and composition) from the portion cost.

The analysis was conducted using *Stata 15* and the *svy* family of commands, which allows survey characteristics such as weighting and sample design to be incorporated so that the estimates we report are nationally and provincially representative (StataCorp, 2017).

## 3 RESULTS

### 3.1 Food consumption

**Figure 1:**
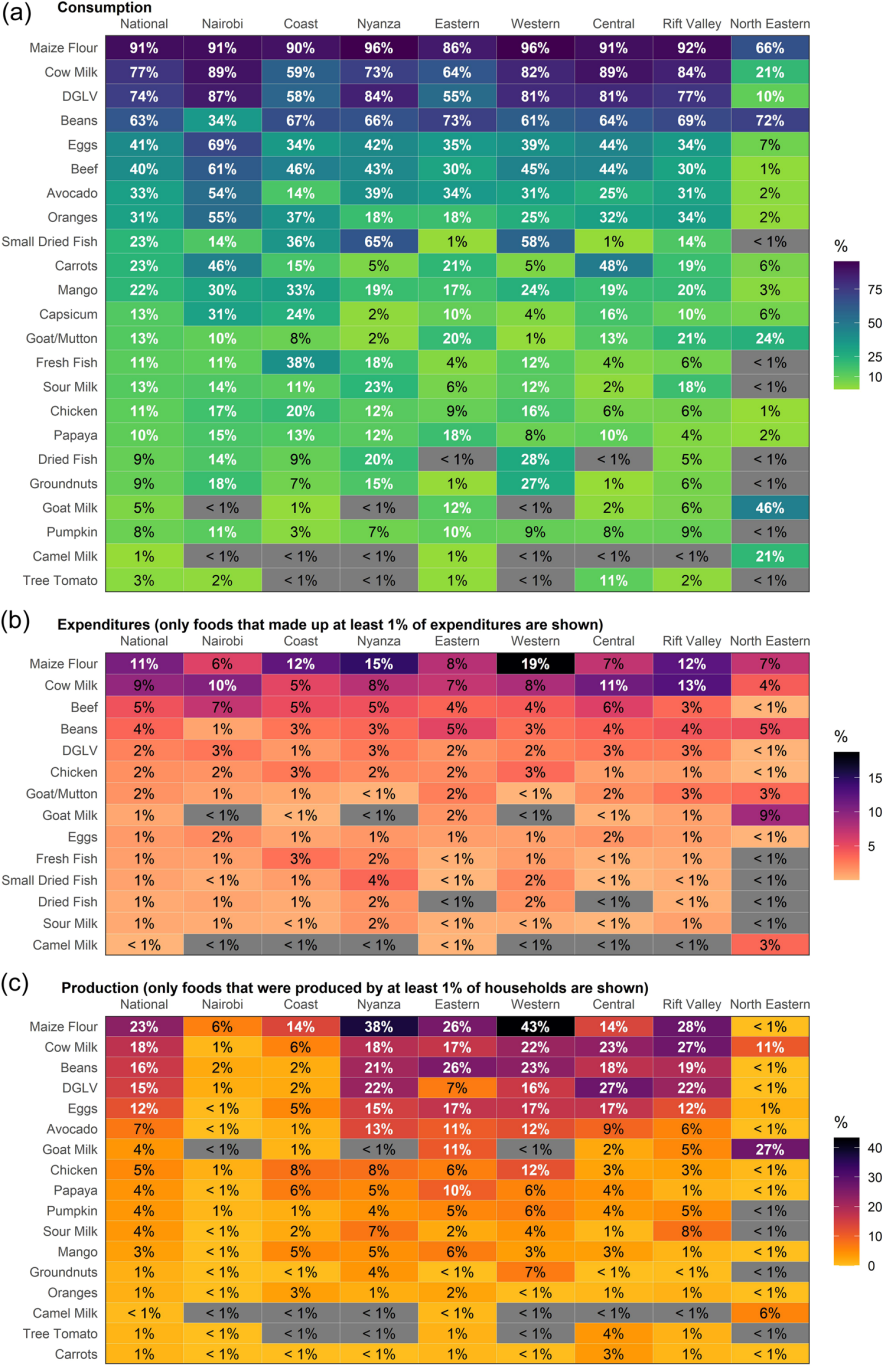
Household food consumption, expenditures and production, by province. (a) Percentage of households consuming each food in the past week. Foods with ≥10% consumption, shown in white, were selected for the affordability analysis for each province (and nationally). Provinces are sorted, from left to right, by the number of foods that were consumed by ≥10% of households. (b) Proportion of total food expenditures that each food accounted for, on average, only for those foods that made up at least 1% of food expenditures for the average household in any province. (c) Percentage of households producing each food in the past week, only for those foods that were consumed from home production by at least 1% of food expenditures in any province. Across all three panels, values are shaded in grey if a food was consumed by <1% of households in a province.

Across all households in Kenya, the most commonly consumed nutritious foods included dark green leafy vegetables, beans and fresh cow milk, all of which were consumed by over half of households in the country in the week before being surveyed (Figure 1a). Eggs, beef and avocado were also consumed by one‐third or more of households. However, even these most commonly consumed foods were consumed less than unfortified maize flour (commonly known as ‘ugali’), an energy‐dense but nutrient‐poor staple.

There is substantial provincial variation in foods consumed, with four distinct patterns observed. In the North Eastern province, much of which has a relatively arid climate, 10% of households or fewer consumed any of the fruits or vegetables included in this analysis, including dark green leafy vegetables. It was more common to consume goat, mutton or camel products than beef products (such as milk and meat) in the North Eastern province. In the Coast province, the only province in Kenya that borders the ocean and in the Western and Nyanza provinces, which border Lake Victoria, consumption of several different categories of fish (fresh, dried or small dried fish, more commonly known as ‘omena’) was more common. In Eastern, Central and Rift Valley provinces, fish consumption was less common. Dietary diversity was generally the greatest and consumption varied more across households in Nairobi.

The only nutritious foods to which households allocated more than 5% of food expenditures were milk (cow milk in all provinces except goat milk in North Eastern), beef and beans (Figure 1b). In many provinces (except Central, Nairobi and North Eastern), maize flour (ugali) accounted for a greater share of expenditures than any of the nutritious foods.

Many of the most commonly consumed foods overall were also the same foods that were likely to be consumed from home production, including milk, beans, dark green leafy vegetables, eggs and avocado, but not including beef (Figure 1c). In Nairobi and the Coast province, none of the foods were consumed from home production by 10% or more of households. Only milk was consumed from production by 10% or more of households in the North Eastern province.

County‐level analysis of food consumption revealed some variation within provinces (Supporting Information: Appendix Figure 4). Counties within a province varied in fish consumption and whether beef or goat/mutton was more commonly consumed. Beyond these differences, some provinces had certain outlier counties with lower dietary diversity and different dietary patterns than the provincial averages, often including less consumption of animal‐source foods, vegetables and fruits. In the Eastern province, compared to other counties, Kitui, Marsabit and Isiolo had lower consumption of eggs and some fruits and vegetables and Marsabit and Isiolo had lower consumption of chicken. Turkana and Samburu counties in the Rift Valley province exhibited lower consumption of dark green leafy vegetables, far few households in Turkana consumed eggs or any type of milk and there was lower consumption of several other fruits and vegetables in Turkana, Samburu and West Pokot. In the Coast province, Tana River had a much lower consumption of eggs, chicken, beef, fish and several fruits and vegetables than the remaining counties. There were also different consumption patterns across the three counties in the North Eastern province (Garissa, Mandera and Wajir).

### 3.2 Food prices

**Figure 2:**
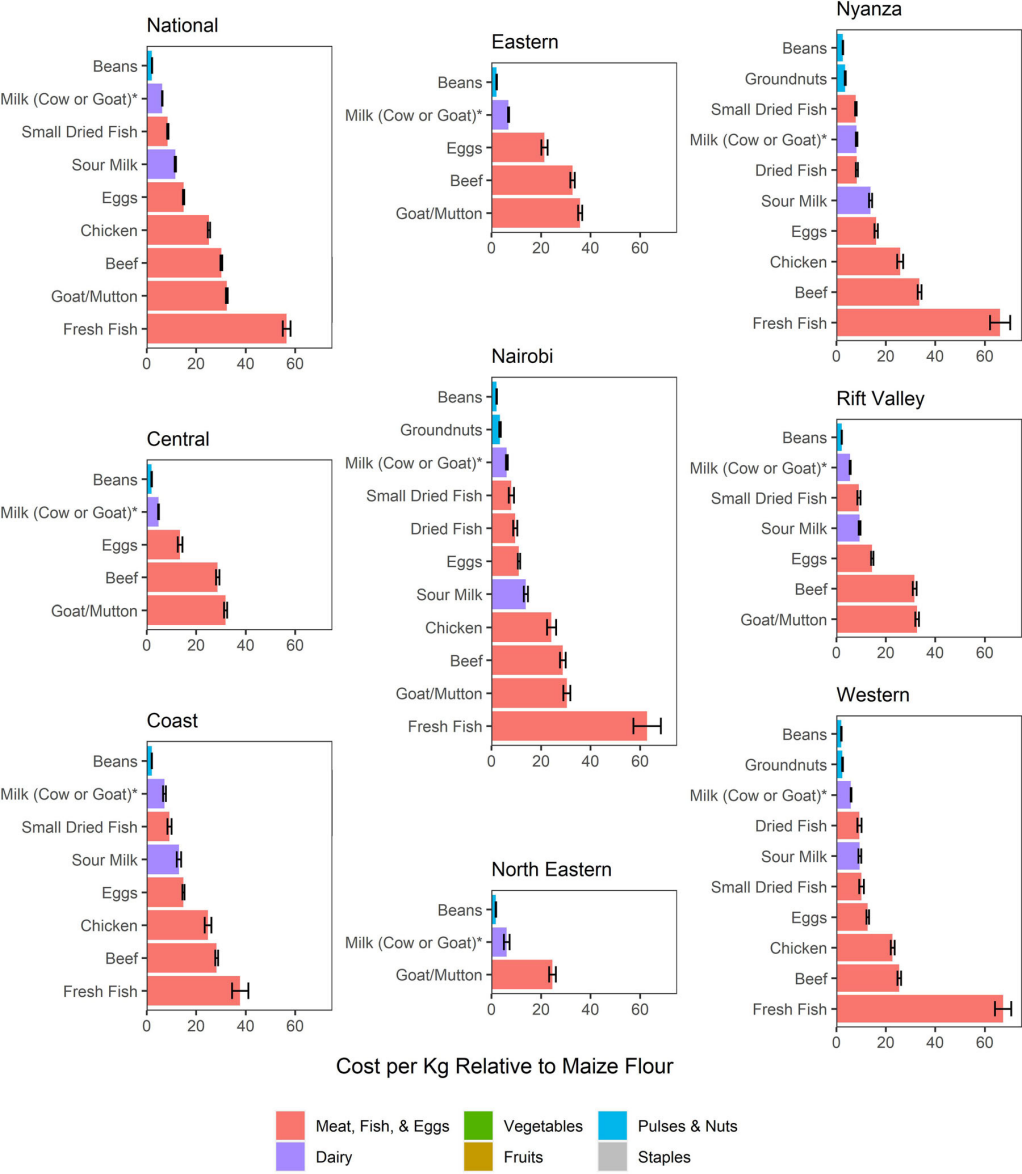
Food cost per kcal relative to maize flour (ugali). *Cow milk is shown for all provinces except North Eastern, where goat milk was more commonly consumed. Foods are only included in the figure if they were consumed by ≥10% of households in a province and supply at least 80 kcal in a 100 g portion (50 g for milk). Liver was excluded because price data separate from flesh meat were not available (see details in Section 2, [Link]). Error bars indicate 95% CIs on the means. Maize flour was selected as the comparator because it was the most commonly consumed staple.

We compared the price per kcal for those foods that supplied at least 80 kcal in a 100 g portion (50 g for milk) and were consumed by at least 10% of households in a province against the price per kcal of unfortified maize flour (ugali) (Figure 2). Ugali was selected as a comparator because it was found to be the most commonly consumed low‐cost and low‐nutritional‐value food in the consumption analysis. All foods cost significantly more than this staple. Plant‐source foods (beans and, in some provinces, groundnuts) were the lowest‐cost per kcal nutritious foods, followed by dairy (milk and sour milk). Small dried fish (omena), dried fish and eggs were the next lowest‐cost nutritious foods on a per kcal basis, while beef, goat/mutton, chicken and fresh fish were the most expensive. While there were some differences on average prices across provinces, these patterns were not consistent across foods.

### 3.3 National affordability by single nutrient

**Figure 3:**
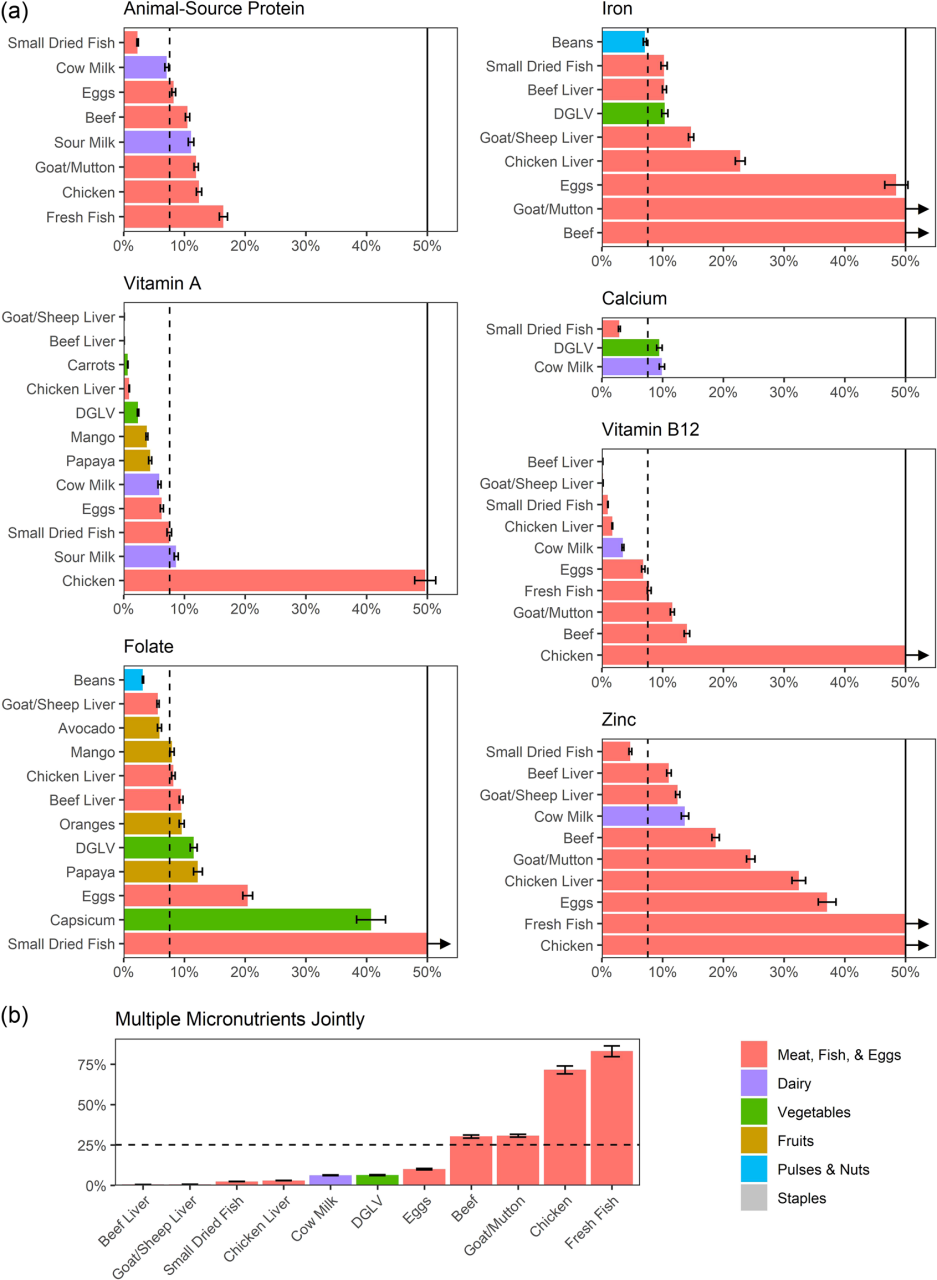
Cost of food portions that could meet half of the single nutrient requirements or an average of one‐third of joint nutrient requirements, as a share of adjusted household food expenditure (national level). Bars in panel A were truncated at 50%; arrows indicate that the true values extend beyond 50%. The dashed lines in (a) indicate the adjusted single nutrient affordability threshold of 7.6%; bars below that threshold are considered affordable for the average household. In (b), the adjusted affordability threshold is higher (25.2%) because this panel shows the cost of foods that could meet an average of one‐third of joint requirements for all six micronutrients (vitamin A, folate, iron, calcium, vitamin B_12_, zinc). Error bars indicate 95% CIs on the means. Costs are displayed as a percentage of household food expenditures. The thresholds of 7.6% and 25.2% were selected by adjusting the 10% and 33.3% thresholds used in previous affordability analyses for the lower average food expenditures among households with children aged 6–23 months in Kenya, as observed in the survey data.

Nationally, iron, calcium, zinc and animal‐source protein stood out as unaffordable nutrients: zero foods or only one food could meet half of the daily complementary feeding requirements at an average cost per adjusted household food expenditure that falls below the affordability threshold of 7.6% (Figure 3a). The only food to affordably meet calcium, zinc and animal‐source protein needs was small dried fish (omena). Beans were the lowest‐cost source of iron. Cow milk and eggs were marginally affordable protein sources.

There were several foods that could meet half of the requirements for vitamin A, folate and vitamin B_12_ at even lower affordability thresholds (<5% of adjusted household food expenditure). Goat/sheep liver, beef liver, chicken liver, carrots, dark green leafy vegetables, mango and papaya (vitamin A); beans, goat/sheep liver and avocado (folate); and beef live, goat/sheep liver, small dried fish (omena), chicken liver, milk and eggs (vitamin B_12_) were affordable sources of these nutrients.

### 3.4 National affordability across multiple nutrients

Small dried fish (omena), liver, milk and eggs were also among the most affordable foods when multiple micronutrient requirements were considered in combination (Figure 3b). Dark green leafy vegetables were also affordable when all six micronutrients were considered jointly and were the only plant‐source food considered in this analysis because no other plant‐source foods (that were consumed by 10% of households) could meet an average of one‐third of joint requirements with a portion size of 100 g or less. As in the single nutrient analysis, beef, goat/mutton, chicken and fresh fish were largely unaffordable.

### 3.5 Provincial affordability

The nationally representative averages displayed in Figure 3 mask subnational variation in affordability. Vitamin A, vitamin B_12_ and folate remain affordable for the average household in all eight provinces, via beef or goat/sheep liver (both vitamins) and beans (folate) (Table 2, Supporting Information: Appendix Figures 5 and 6). Folate (via beans) costs relatively more for households in the North Eastern province than in the other provinces. For most provinces, dark green leafy vegetables and orange‐fleshed fruits and vegetables were also affordable sources of vitamin A (except in North Eastern), milk was an affordable source of vitamin B_12_ (camel milk in North Eastern, cow milk in other provinces) and avocado was an affordable source of folate (except in North Eastern and Coast).

**Table 2:** Summary of provincial food and nutrient affordability.

	Animal‐source protein	Vitamin A	Folate	Iron	Calcium	Vitamin B_12_	Zinc
Central	Cow milk, eggs	Goat liver, beef liver, carrots	Beans, avocado, goat liver	Beans, DGLV	DGLV, cow milk	Beef liver, goat liver, cow milk	‐
Coast	Omena	Beef liver, chicken liver, carrots	Beans, mango	Beans	Omena	Beef liver, omena, chicken liver	Omena
Eastern	Cow milk	Goat liver, beef liver, carrots	Beans, mango, goat liver	Beans	‐	Beef liver, goat liver, cow milk	‐
Nairobi	Omena, dried fish, eggs	Goat liver, beef liver, carrots	Beans, avocado, goat liver	DGLV, beans, omena	Omena, DGLV, cow milk	Beef liver, goat liver, omena	Omena, beef liver
North Eastern	‐	Goat liver	Beans	‐	‐	Goat liver, camel milk	‐
Nyanza	Omena, dried fish	Beef liver, chicken liver, DGLV	Beans, avocado	‐	Omena	Beef liver, omena, chicken liver	Omena
Rift Valley	Omena, cow milk	Goat liver, beef liver, carrots	Beans, avocado, goat liver	‐	Omena	Beef liver, goat liver, omena	Omena
Western	Omena, dried fish	Beef liver, chicken liver, DGLV	Beans, avocado, mango	‐	Omena	Beef liver, omena, chicken liver	Omena

*Note*: The table lists foods that fall below the adjusted affordability threshold of 7.6% for each nutrient and province. The threshold of 7.6% was selected by adjusting the 10% threshold used in previous affordability analyses for the lower average food expenditures among households with children aged 6–23 months in Kenya, as observed in the survey data. Abbreviation: DGLV, dark green leafy vegetables.

**Figure 4:**
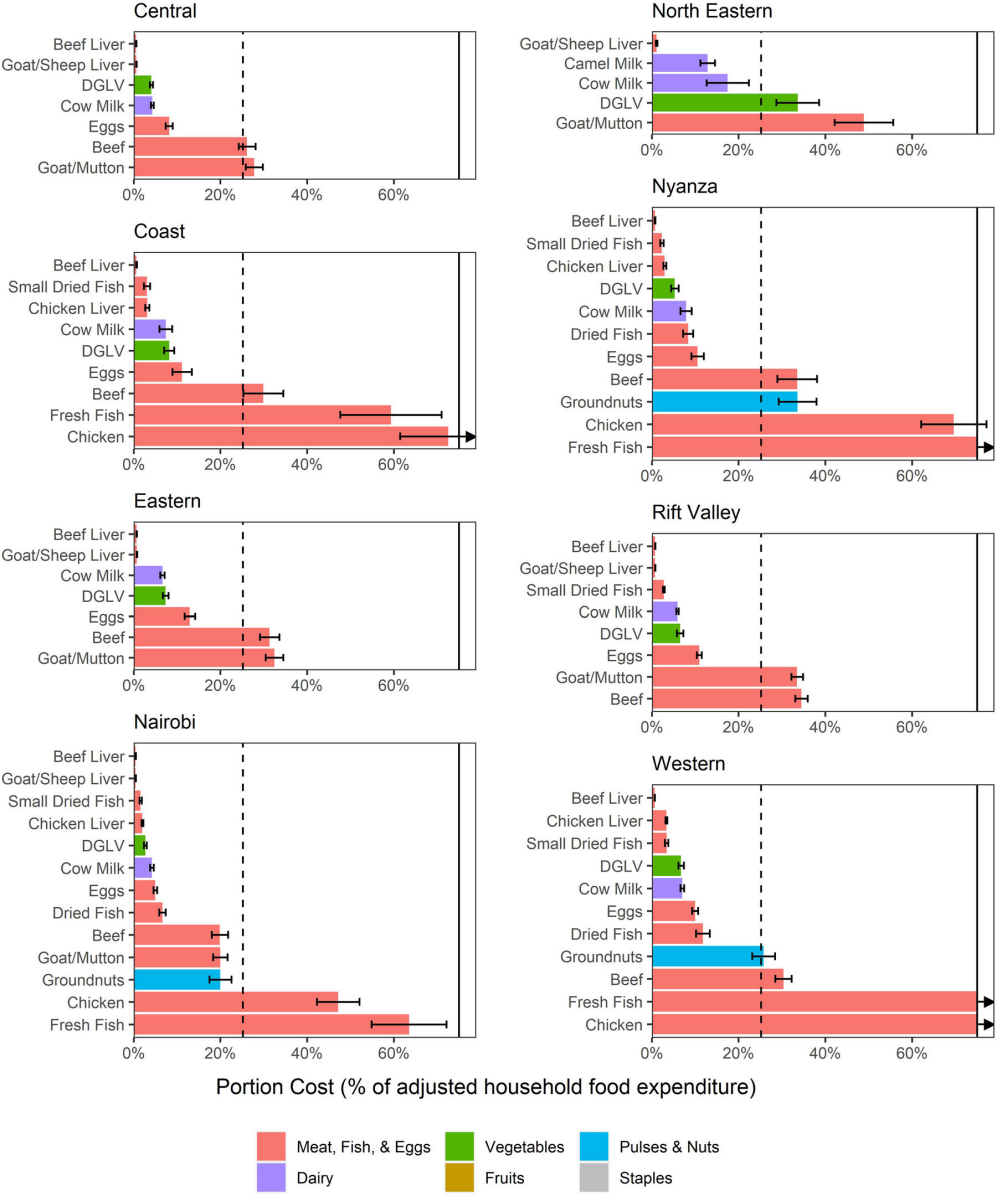
Cost of food portions that could meet one‐third of joint nutrient requirements, as a share of adjusted household food expenditures, by province. Bars were truncated at 75%; arrows indicate that the true values extend beyond 75%. The dashed lines indicate the adjusted joint nutrient affordability threshold of 25.2%; bars below that threshold are considered affordable for the average household. Error bars indicate 95% CIs on the means. The threshold of 25.2% was selected by adjusting the 33.3% threshold used in previous affordability analyses for the lower average food expenditures among households with children aged 6–23 months in Kenya, as observed in the survey data.

Liver (beef or goat/sheep) was also the most affordable food to meet micronutrient requirements jointly in all provinces and cost substantially less than the joint nutrient affordability threshold (Figure 4). Cow milk, dark green leafy vegetables and eggs were also affordable sources of multiple micronutrients in all provinces, except in North Eastern, where dark green leafy vegetables were slightly above the threshold and eggs were not included because of low consumption. Small dried fish (omena), chicken liver, dried fish and camel milk were affordable sources of multiple micronutrients in all the provinces for which they were considered but were only considered for a subset of provinces because consumption was highly variable.

There was greater variation in affordability and the most affordable foods, to meet iron, calcium, zinc and animal‐source protein requirements; these were also the least affordable nutrients nationally. Protein, calcium and zinc cost substantially more for households in the Eastern and North Eastern provinces. In these two provinces, there were no foods that fell below the affordability threshold for the average household. Milk was the most affordable food to meet protein and calcium requirements and liver (beef or goat) was the most affordable food to meet zinc requirements, while in most of the other provinces, small dried fish (omena) was the most affordable source of all three nutrients. Omena was not included for Eastern, North Eastern and Central because it was consumed by few households. Omena was consumed by over 10% of households in the Rift Valley and Coast provinces and thus was included in the analysis. However, omena consumption is uncommon in several counties in these provinces [<10% of households consumed omena in Baringo, Bomet, Kajiado, Laikipia, Samburu, Turkana and West Pokot (Rift Valley) and Lamu and Tana River (both Coast); Supporting Information: Appendix Figure 4]. Although we were unable to assess county‐level affordability, it is likely that affordability of protein, zinc and calcium will also be challenging for households in these counties, as other options to meet the needs of these nutrients cost much more than omena (Supporting Information: Appendix Figure 5).

Overall, iron was only affordable at the base threshold of 7.6% to the average households in only four provinces: Central (beans and dark green leafy vegetables), Nairobi (beans, small dried fish/omena, beef liver and dark green leafy vegetables), Eastern (beans) and Coast (beans). Beans were the lowest‐cost source of iron in all provinces except Nairobi and Nyanza. In these two provinces, dark green leafy vegetables were more affordable. These two provinces also had the highest baseline consumption levels of dark green leafy vegetables.

### 3.6 Sensitivity analysis

**Figure 5:**
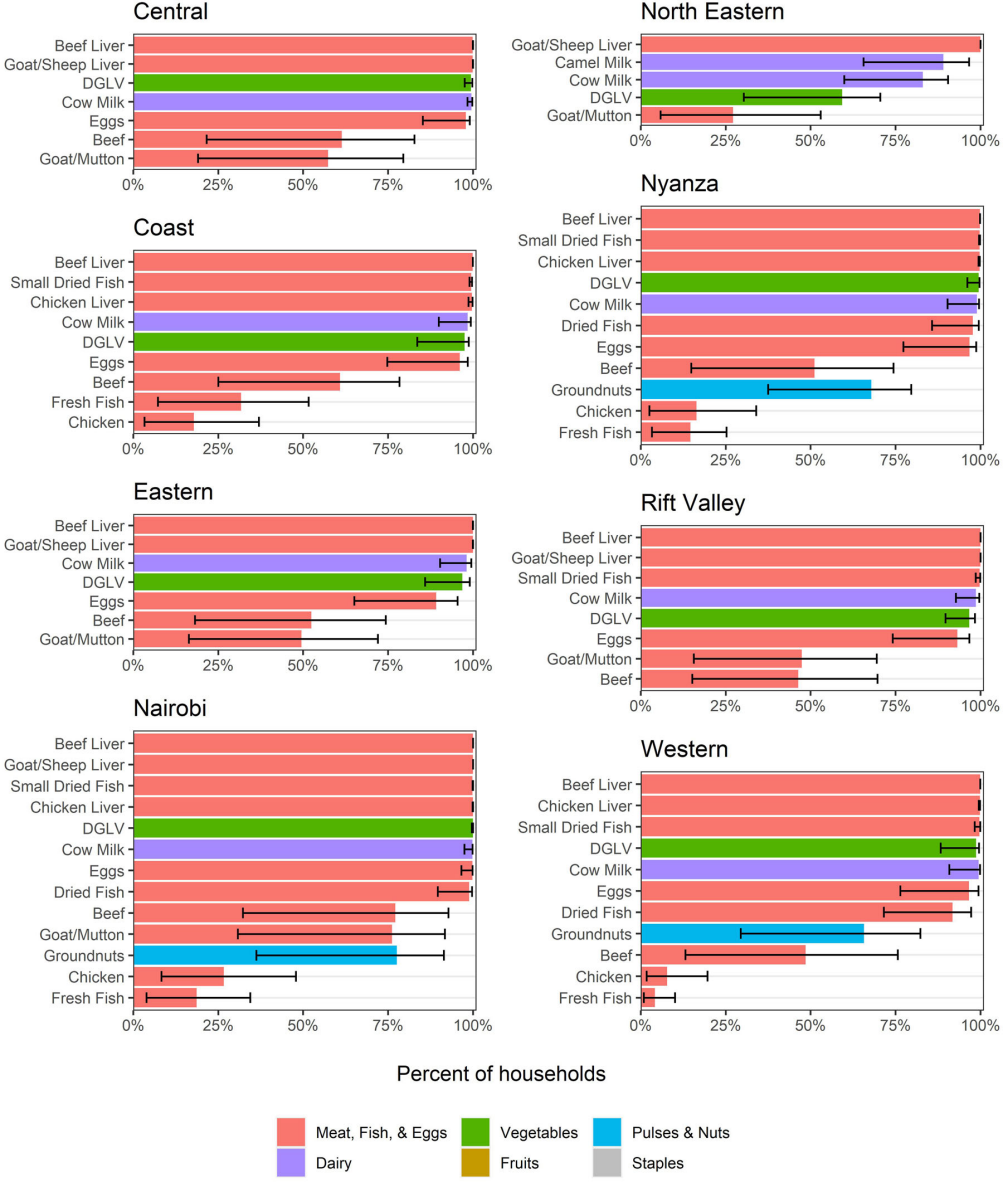
Percent of households that can afford to meet one‐third of joint nutrient requirements at various affordability thresholds, by province. The figure shows the percentage of households that can afford each food at the base affordability threshold (filled bars; 25.2%), a lower threshold (lower errors bars; half the base threshold, requiring that foods cost less to be considered affordable) and a higher threshold (higher error bars; 50% higher than the base threshold, allowing foods to cost more and still be considered affordable).

Several foods, including small dried fish (omena), milk, dark green leafy vegetables and eggs, were considered affordable sources of multiple micronutrients in combination for most households in most provinces at stricter affordability thresholds (Figure 5). This was also the case for vitamins A and B_12_ (via liver, Supporting Information: Appendix Figures 7 and 8). However, the affordability of calcium, protein and folate depended more on the affordability threshold and there were fewer options to affordably meet the requirements for these nutrients. Even though beans (as a source of iron) fell below the 7.6% threshold for the average household, at this threshold, they were unaffordable for more than one in four households. Similarly, small dried fish (omena) were an unaffordable source of zinc for around one in five households. The remaining foods (milk, eggs, dark green leafy vegetables) that could meet half of iron and zinc requirements from complementary foods were unaffordable even at more relaxed affordability thresholds.

Although the KIHBS did not contain data on which household members consumed foods, we can estimate what each household member is currently consuming, assuming that foods are split equally across household members based on their adult equivalents (which are based on energy requirements). This assumption could underestimate current child consumption and overestimate affordability since children may consume less as a share of their nutritional needs than other household members (Behrman & Deolalikar, 1990; Fadare et al., 2019; Rathnayake & Weerahewa, 2002; International Food Policy Research Institute, 1997). In this analysis, two commonly consumed foods—milk and beans—looked substantially more affordable and dark green leafy vegetables, which are also commonly consumed in most provinces, are somewhat more affordable (Supporting Information: Appendix Figures 9 and 10). This result is common across provinces, except North Eastern and, to some extent, Eastern and Coast. In these provinces, especially North Eastern, average expenditures on dark green leafy vegetables and milk were lower. Affordability of the remaining foods in most provinces remained similar to the base analysis, indicating that current consumption levels are relatively low compared to requirements. While nutrient gaps persist among children of complementary feeding age despite common consumption of these foods, this sensitivity analysis reveals that milk, beans and dark leafy green vegetables may be more affordable than the base analysis suggests, especially if households are able to reallocate current consumption to young children.

The affordability of several foods and nutrients that are close to the affordability threshold, including beans (as a source of iron), dark green leafy vegetables (calcium, folate) and small dried fish (omena; iron, zinc, vitamin A) varied, nationally and for most provinces, in an analysis that considered variation in nutrient densities (Supporting Information: Appendix Figure 11). There appears to be especially large variability in the nutrient composition and, thus potentially the affordability of small dried fish (omena).

## 4 DISCUSSION

### 4.1 Summary of findings and implications

This study assessed the affordability of foods that could provide nutrients commonly lacking during the complementary feeding period in Kenya. We focused on foods that are already locally available and consumed by households. Affordability varied widely by food and depended on the nutrients and provinces considered. Where available, small dried fish (omena), liver, milk, dark green leafy vegetables and eggs were generally affordable sources of multiple micronutrients of concern. Interventions that focus on these foods could yield broad benefits, potentially even beyond the nutrients considered here. Most of the lowest‐cost foods that could meet multiple nutrient requirements were animal‐source foods. Plant‐source foods tend to be cheaper calorically in both Kenya and other countries in the region (Headey & Alderman, 2019; Ryckman, Beal, Nordhagen, Chimanya, et al., 2021), illustrating the importance of looking beyond energy density.

### 4.2 Findings and policy implications related to more affordable nutrients

Liver and beans are affordable sources of vitamin A, vitamin B_12_ and folate to almost all households in Kenya. Interventions could focus on increasing consumption of these foods. Although consumption of either beef or goat/mutton was common across provinces, few households reported consuming the liver of these animals. More research is needed on the feasibility of increasing liver consumption; for example, interventions may focus on the availability of small quantities of the liver for purchase separately from flesh meat (UNICEF Kenyatta University Johns Hopkins University & GAIN, 2020, 2021).

Beans are commonly consumed but may not be consumed in large enough quantities by young children to meet half of their folate needs. Beans could be promoted further to help meet folate needs in addition to other essential nutrients and could be supplemented by smaller quantities of other moderately affordable foods if portion sizes are too large. These moderately affordable foods vary more by province. In many provinces, dark green leafy vegetables and orange‐fleshed fruits and vegetables were affordable sources of vitamin A and folate. Dark green leafy vegetables are already commonly consumed in all provinces except North Eastern, but the amounts consumed may be low, they may be fed less commonly to young children, and/or vitamin A absorption from plant‐source foods may be insufficient (von Lintig et al., 2020). Combinations of these foods could be considered in settings where changing habits to increase liver consumption is not feasible.

### 4.3 Findings and policy implications related to less affordable nutrients

Calcium, animal‐source protein, iron and zinc were less affordable and there was more provincial variation in affordability. Calcium, animal‐source protein and zinc affordability were strongly related to the availability of small dried fish (omena). Where omena was included in the analysis (Nyanza, Western, Coast, Rift Valley, Nairobi), it was the most affordable source of these nutrients. In the remaining provinces (North Eastern, Eastern, Central), omena consumption was rare, calcium, animal‐source protein and zinc were less affordable and the most affordable sources of these nutrients varied. The increasing availability of and demand for omena, where it was less commonly consumed (which also includes some counties in Rift Valley and Coast), could help address calcium, protein and zinc gaps. In other provinces, efforts may need to focus on increasing the desirability of omena as a complementary food. Eggs and milk were borderline affordable sources of protein and affordable sources of other nutrients (vitamin A, vitamin B_12_) in all provinces except North Eastern. These foods could be the focus of interventions both to reduce prices and to increase consumption and/or home production.

Zinc and iron were generally the least affordable nutrients and only one or no sources were affordable per province. Beans and dark green leafy vegetables were the most affordable sources of iron in all provinces. Consumption data suggest many children may already be consuming beans and dark green leafy vegetables, but the daily portions required to meet half of the iron requirements from complementary feeding are large for a young child. Thus, the scope for further increasing consumption may be limited. As beans are high in phytate, which can inhibit the absorption of iron and other minerals, it may be important to focus on including sources of heme‐iron for young children, for example, via small additions of liver and small dried fish (omena) to meals. Consumption of flesh foods and vitamin C‐rich foods may also increase absorption of nonheme iron, independent of iron status (Reddy et al., 2000). Supplementation and fortification could also be considered to increase iron and zinc consumption. The promotion of biofortified beans, which have greater density and bioavailability of iron and zinc, is also worth considering (Hummel et al., 2020).

### 4.4 Other policy considerations

The North Eastern province had the lowest dietary diversity and faced the greatest affordability barriers. Fewer nutritious foods were commonly consumed, household resources were lower and the prices of some foods (notably dark leafy green vegetables) were higher. Nutrients were also less affordable in the Eastern and Central provinces, largely due to the presumed low availability of small dried fish (omena). North Eastern and some counties in other provinces (e.g., Samburu, Turkana, Marsabit, Kitui, Isiolo, Tana River) could be the focus of additional interventions aimed at increasing availability and reducing the prices of more diverse diets. Behaviour change interventions may also be needed. North Eastern province is comprised largely of arid‐ and semiarid lands, limiting agricultural crop possibilities (as evidenced by production patterns in the province; see Figure 1b) and has much lower levels of educational outcomes, antenatal care provision and child vaccination coverage than the rest of the country, highlighting the potential challenges in delivering programs to address child feeding gaps in this population (Kenya National Bureau of Statistics et al., 2015). However, there is great potential for sustainably improving the production of livestock, which can make use of marginal arid lands unsuitable for crop production (Rakotoarisoa et al., 2008).

While this paper focused on affordability, policies need to consider household food consumption more holistically. Other barriers, such as low demand (e.g. due to knowledge, cultural practices, convenience/time) or access (e.g. subprovincially or seasonally) are not addressed by this paper and could be the subject of future research. Ethnographic research in two Kenyan counties elsewhere in this supplement (Kimiywe et al., 2022) indicates that a lack of strategies to modify foods that are commonly consumed but not generally given to young children, such as eggs, is one important barrier. Food handling procedures (drying, preservation, storage, transportation) are also critical for many foods, such as omena, as they impact nutritional value, palatability and safety. Some foods, such as beans, take longer to cook, impacting nonfood fuel and time costs (Masters et al., 2021). Future research should assess these and other potential barriers to optimal complementary feeding more comprehensively, to identify their relative importance in each context and inform effective policymaking. Additionally, while this paper is focused on specific foods and nutrients, increasing households' purchasing power, for example, via social protection or subsidy programs, also improves affordability by increasing resources available for food (Traore et al., 2022).

### 4.5 Comparison with other literature

An analysis of complementary feeding affordability in other countries in Eastern and Southern Africa also found that vitamin A, vitamin B_12_ and folate are the most affordable nutrients and iron and especially zinc were the least affordable (Ryckman, Beal, Nordhagen, Chimanya, et al., 2021). The present study is also consistent with a cost of diet analysis conducted in Turkana county (Rift Valley), which found that beans and dark green leafy vegetables were among the most affordable commonly consumed foods to meet 6‐23 month‐old children's nutrient needs (‘A Cost of the Diet Analysis in Turkana County, Kenya’, Resource Centre, 2017). That analysis also found that meeting iron and zinc requirements through commonly consumed foods would be challenging. This convergence of findings regarding the relative unaffordability of iron and zinc further highlights the potential need for other interventions, such as fortification, biofortification, and/or supplementation.

### 4.6 Strengths and limitations

This study builds on the existing affordability literature by stratifying the analysis by both nutrient and province. Our findings underscore the importance of considering subnational variation in a diverse country such as Kenya. For example, had the analysis been conducted at the national level only, we would have concluded that the average household could afford at least one source of each nutrient. subnational analysis reveals that this is not the case in several provinces. Our findings can be used to inform more targeted research and programs focused on specific foods and settings.

Although we were unable to assess county‐level affordability, we did find evidence of within‐province heterogeneity in food consumption patterns, underscoring the likelihood of more granular geographic variation in affordability. Future work could include a more focused analysis of counties in the Coast, Eastern, North Eastern and Rift Valley provinces, where heterogeneity in consumption was greatest. Additionally, the thresholds we used to determine absolute affordability are somewhat arbitrary. However, most conclusions of this study are not dependent on the threshold. Furthermore, wild‐harvested foods were not captured in the survey, but other analyses have found that several wild foods could reduce the cost of iron, zinc and calcium in Kenya (‘A Cost of the Diet Analysis in Turkana County, Kenya’, Resource Centre, 2017; Sarfo et al., 2020; Termote et al., 2014). Finally, while we consider household food costs, we did not consider the costs of proposed interventions that could reduce costs or increase consumption. These costs would presumably be covered by a government or external funder and we leave an analysis of the benefits and costs of these interventions as future work.

## 5 CONCLUSION

Addressing the burden of undernutrition in Kenya depends on improving the diets of young children during the critical complementary feeding period. While many programs focus on caregiver knowledge and practices, their uptake depends on caregivers being able to affordably access nutrient‐dense foods. Our findings highlight the importance of interventions that can increase purchasing power (e.g., by increasing incomes or providing social protection programs) as well as reducing prices for nutrient‐dense foods (e.g., through subsidies, increasing food production and strengthening markets). For some nutrients, such as iron and zinc, we found that additional solutions (e.g., fortification and supplementation) are likely needed, at least in the short term. We also highlighted foods that are already affordable, including as sources of multiple micronutrients and could thus be effectively promoted more widely. The findings can thus be used to inform policies and programs, as well as future research on how to achieve optimal complementary feeding for young children in Kenya.

## AUTHOR CONTRIBUTIONS

Theresa Ryckman and Ty Beal designed the research. Theresa Ryckman was responsible for compiling and cleaning the survey data and running the analysis. Ty Beal and Theresa Ryckman were involved in methodology development. Theresa Ryckman, Ty Beal and Stella Nordhagen contributed to the initial draft of the manuscript. Ty Beal was responsible for the data and analysis related to nutrient requirements and portion sizes. All authors contributed to writing, revisions and interpretation and contextualization of results. All authors have read and approved the final manuscript.

## CONFLICTS OF INTEREST

The authors declare no conflicts of interest.

## Supporting information

Supporting information.

## Data Availability

All data used in this study were obtained from publicly available sources that are cited in the references.
