# Supplementary material for: A subnational affordability assessment of nutritious foods for complementary feeding in Kenya
Source: Matern Child Nutr. 2022 Jun 6;20(Suppl 3):e13373. doi: 10.1111/mcn.13373 (PMC10782139; doi:10.1111/mcn.13373)
Supplement: Supplementary file 1 — Supporting information. [file MCN-20-e13373-s001.docx]

**Appendix for “A subnational affordability assessment of nutritious foods for complementary feeding in Kenya”**

# Supplementary Methods: Portion size calculations

We used data from Kenya food composition tables to calculate portion sizes that could meet half of daily nutrient requirements for each food-nutrient combination (Mwai et al., 2018). These tables included data on the nutrient densities and refuse of numerous foods, many of which overlapped with foods tracked in the KIHBS. We supplemented the data from the Kenya food composition tables with data from published sources and other regional food composition tables on cooking yields and on nutrient densities for two foods: dark green leafy vegetables and small dried fish (commonly known as “omena” in Kenya) (Bognar, 2002; Kabahenda, Amega, Okalany, Husken, & Heck, 2011; Korkalo, Hauta-alus, & Mutanen, 2011; Lukmanji et al., 2008; Nyirenda, Musukwa, Mugode, & Shindano, 2009; Roseland et al., 2014; Stadlymayr et al., 2012; Steiner‐Asiedu, Lied, Lie, Nilsen, & Julshamn, 1993; U.S. Department of Agriculture, Agricultural Research Service, 2019). For these two foods, we included additional sources to account for variation in nutrient densities across several varieties of the food.

# Appendix Table 1: Nutrient requirements from complementary foods for children aged 6-23 months

| **Nutrient** | **Requirement type** | **Animal or plant source*** | **Nutrient Requirements (by age)** | | | **Proportion of nutrient requirements needed from complementary feeding**** |
| --- | --- | --- | --- | --- | --- | --- |
|  |  |  | **6-11 m** | **12-35 m** | **6-23 m (estimated)** |  |
| Protein (g) | RDA | Animal source foods | 11.0 | 13.0 | 12.3 | 0.49 |
| Iron (mg) | RNI | Animal source foods | 6.2 | 3.9 | 4.7 | 0.98 |
| Iron (mg) | RNI | Plant source foods | 9.3 | 5.8 | 7.0 | 0.98 |
| Zinc (mg) | RNI | Animal source foods | 2.5 | 2.4 | 2.4 | 0.87 |
| Zinc (mg) | RNI | Plant source foods | 4.1 | 4.1 | 4.1 | 0.87 |
| Calcium (mg) | RNI | Both | 400.0 | 500.0 | 466.7 | 0.65 |
| Vitamin A (mcg RAE) | RDA | Both | 500.0 | 300.0 | 366.7 | 0.17 |
| Vitamin B12 (mcg) | RDA | Both | 0.5 | 0.9 | 0.8 | 0.70 |
| Folate (mcg DFE) | RDA | Both | 80.0 | 150.0 | 126.7 | 0.60 |

*For iron, we assumed 15% dietary iron bioavailability for animal-source foods and 10% for plant foods; for zinc we assumed 50% dietary zinc bioavailability for animal-source foods and 30% for legumes, nuts, and seeds (World Health Organization & Food and Agriculture Organization of the United Nations, 2004). **Based on (Dewey, 2001).

# Appendix Table 2: Variation in nutrient densities of dark green leafy vegetable varieties

| **Food** | **Food Composition Table (Source)** | **Nutrient Content** | | | |
| --- | --- | --- | --- | --- | --- |
|  |  | **Iron (mg)** | **Vitamin A (mcg RAE)** | **Calcium (mg)** | **Folate (mcg DFE)** |
| Amaranth (boiled) | Kenya (Mwai et al., 2018) | 5.3 | 309 | 280 | 34 |
| Amaranth (stewed) |  | 8.3 | 362 | 280 | 55 |
| Kale/Sukuma Wiki (boiled) |  | 2 | 152 | 364 | 30 |
| Kale (steamed) |  | 2 | 139 | 332 | 33 |
| Ethiopia Kale (boiled) |  | 4 | 101 | 105 | 35 |
| Ethiopia Kale (steamed) |  | 3.9 | 92 | 96 | 38 |
| Pumpkin Leaves (boiled) |  | 4 | 121 | 347 | 17 |
| Pumpkin Leaves (steamed) |  | 3.9 | 111 | 316 | 19 |
| Spinach (boiled) |  | 1.4 | 179 | 131 | 58 |
| Spinach (stewed) |  | 2.1 | 209 | 161 | 95 |
| Swiss Chard (boiled) |  | 3.5 | 180 | 117 | 64 |
| Swiss Chard (stewed) |  | 5.4 | 211 | 144 | 105 |
| Cowpea Leaves (boiled) |  | 1.9 | - | 170 | 45 |
| Cowpea Leaves (steamed) |  | 1.9 | - | 155 | 49 |
| Amaranth Leaves | Mozambique (Korkalo et al., 2011) | 3.8 | - | 407 | 25 |
| Cowpea Leaves |  | 2.3 | 242 | 97 | 18 |
| Pumpkin Leaves |  | 1.2 | 269 | 67 | 26 |
| Sweet Potato Leaves |  | 2.4 | 233 | 98 | 17 |
| Cassava Leaves | Zambia (Nyirenda et al., 2009) | 3.7 | - | - | - |
| Cowpea Leaves |  | 5.7 | - | - | - |
| Pumpkin Leaves |  | 5.6 | - | - | - |
| Rape (Brassica Carinata) |  | 5.5 | - | - | - |
| Amaranth Leaves | West Africa (Stadlymayr et al., 2012) | 4.9 | 228 | - | 42 |
| Cassava Leaves |  | 4.4 | 271 | - | 62 |
| Cowpea Leaves |  | 4.0 | 142 | - | 68 |
| Pumpkin Leaves |  | 1.8 | - | - | 19 |
| Spinach |  | 2.5 | 387 | - | 93 |
| Sweet Potato Leaves |  | 2.8 | 463 | - | 42 |
| Amaranth Leaves | USDA (U.S. Department of Agriculture, Agricultural Research Service, 2019) | 2.3 | 139 | 209 | 57 |
| Mustard Greens |  | 3.2 | 80 | 43 | 25 |
| Pumpkin Leaves |  | 0.6 | 147 | 33 | 49 |
| Sweet Potato Leaves |  | 3.6 | 524 | 136 | 146 |
| Spinach |  | 0.9 | 527 | 118 | 9 |
| Median [10^th^ and 90^th^ percentiles] | | 3.5  [1.5-5.5] | 210 [106-428] | 144 [73-344] | 42 [18-93] |

All nutrient densities are shown per 100 grams of a food.

# Appendix Table 3: Variation in nutrient densities of small dried fish varieties

| **Small Dried Fish** | | | | | | | | |
| --- | --- | --- | --- | --- | --- | --- | --- | --- |
| **Food** | **Location and Source** | **Nutrient Content** | | | | | | |
|  |  | **Iron (mg)** | **Zinc (mg)** | **Calcium (mg)** | **Vitamin B_12_ (mcg)** | **Vitamin A (mcg RAE)** | **Protein (g)** | **Energy (kcal)** |
| Dried Dagaa/ Omena | Kenya FCT (Mwai et al., 2018) | 6.9 | 16.0 | 2790 | 60.0 | 141 | 59.5 | 333 |
| Small Dried Fish | Mozambique GAIN/CGIAR Analysis (unpublished) | 36.8 | 11.1 | 960 | - | - | 56.7 | - |
| Small Dried Fish | Mozambique FCT (Korkalo et al., 2011) | 33.6 | 6.4 | 3436 | 12.1 | - | 54.7 | 255 |
| Small Dried Fish | Tanzania FCT (Lukmanji et al., 2008) | 2.5 | 5.2 | 1700 | 12.0 | - | 58.6 | 335 |
| Dried Mukene | Lake Victoria region, Kabahenda et al. (Kabahenda et al., 2011) | 10.7 | 10.3 | 1556 | - | - | 58.8 | - |
| Dried Kapenta | Zambia (Steiner-Asiedu et al via Longley et al (Steiner‐Asiedu et al., 1993)) | 10.0 | 10.0 | 2360 | - | 186 | 63.3 | - |
| Median [10^th^-90^th^ percentiles] | | 10.0 [5.1-34.9] | 10.2 [5.8-13.6] | 2360 [1318-3174] | 12.1 [12.0-50.4] | 186 [150-469] | 59 [56-63] | 294 [223-334] |

# All nutrient densities are shown per 100 grams of a food.

# Appendix Table 4: Variation in nutrient densities of beans

| **Food** | **Food Composition Table (Source)** | **Nutrient Content** | | | |
| --- | --- | --- | --- | --- | --- |
|  |  | **Iron (mg)** | **Zinc (mg)** | **Energy (kcal)** | **Folate (mcg DFE)** |
| Kidney Beans | Kenya (Mwai et al., 2018) | 2.1 | 0.96 | 117 | 52 |
| Broad Beans |  | 1.6 | 1.14 | 109 | 45 |
| Lima Beans |  | 3.4 | 0.95 | 120 | 76 |
| Median  [10^th^-90^th^ percentiles] | | 2.1  [1.7-3.1] | 0.96  [0.95-1.10] | 117  [111-119] | 52  [46-71] |

All nutrient densities are shown per 100 grams of a food.

**Appendix Table 5: Edible portion sizes for each food and nutrient**

|  | **Animal-Source Protein** | **Iron** | **Vitamin A** | **Calcium** | **Zinc** | **Folate** | **Vitamin B_12_** |
| --- | --- | --- | --- | --- | --- | --- | --- |
| Avocado | - | - | - | - | - | 56.7 | - |
| Beans | - | 162.6 | - | - | - | 73.1 | - |
| Beef | 10.1 | 81.7 | - | - | 17.9 | - | 13.4 |
| Beef Liver | - | 15.7 | 0.1 | - | 16.8 | 14.4 | 0.2 |
| Capsicum | - | - | - | - | - | 73.1 | - |
| Carrots | - | - | 5.5 | - | - | - | - |
| Chicken | 12.6 | - | 50.3 | - | 96.2 | - | 53.7 |
| Chicken Liver | - | 18.6 | 0.8 | - | 26.5 | 6.7 | 1.4 |
| DGLV | - | 97.5 | 22.4 | 89.2 | - | 108.6 | - |
| Dried Fish | 4.2 | 57.2 | 16.8 | 29.4 | 52.9 | - | 5.4 |
| Eggs | 21.6 | 127.0 | 16.5 | - | 96.2 | 53.5 | 17.9 |
| Fresh Fish | 13.4 | - | - | - | 88.2 | - | 6.4 |
| Goat | 11.0 | 54.4 | - | - | 22.7 | - | 10.7 |
| Goat Liver | - | 21.4 | 0.1 | - | 18.2 | 8.2 | 0.3 |
| Groundnuts | - | - | - | - | 64.4 | 39.2 | - |
| Mango | - | - | 33.9 | - | - | 70.4 | - |
| Milk, Camel | 137.3 | 152.4 | - | - | - | - | 44.7 |
| Milk, Cow | 91.6 | - | 76.0 | 127.5 | 176.4 | - | 44.7 |
| Milk, Goat | 86.3 | - | 97.4 | 84.3 | - | - | 76.7 |
| Milk, Sour | 97.5 | - | 76.0 | - | - | - | - |
| Omena | 5.1 | 22.9 | 16.8 | 6.4 | 10.4 | 102.7 | 2.2 |
| Oranges | - | - | - | - | - | 73.1 | - |
| Papaya | - | - | 36.7 | - | - | 102.7 | - |
| Pumpkin | - | - | 22.8 | - | - | - | - |
| Tree Tomato | - | - | - | - | - | 60.3 | - |

DGLV = dark green leafy vegetables. Portion sizes are shown in grams.

**Supplementary Methods – Joint micronutrient analysis**

We assessed the affordability of foods in terms of their provision of multiple micronutrients combined. For a given portion size *i*, of a given food *j*, the average share of nutrient requirements *Y* for a set of micronutrients *A*, is calculated as:

$$Y_{i,j}=\frac{1}{|A|}\sum_{a \in A} min\{\frac{nutrient\_density_{a,j}*i}{nutrient\_requirements_{a}}, 1\}$$

For each food in the analysis, we calculated the portion size *i* which could provide an average of one-third of nutrient requirements and assessed the affordability of those foods for which this portion size was less than or equal to 100 g. All inputs (nutrient requirements from complementary feeding, nutrient densities, refuse, cooking yield) in the joint micronutrient analysis were the same as in the single nutrient analysis.

Appendix Figures 1-3 demonstrate how the average share of nutrient requirements is calculated across a range of portion sizes and foods. Figures 1-2 show the proportion of daily nutrient needs that are met from portion sizes of different foods for single micronutrients. Figure 3 shows the average share of nutrient requirements of these foods and portions. The levels shown in Figure 3 can be visualized as the averages for single nutrients shown in Figures 1 and 2. The foods included in the joint micronutrient analysis are those for which the height of the colored lines exceeds the height of the horizontal dashed black line (at 33.3%) at portion sizes of 100 g or lower.

**Appendix Figure 1: Average share of single micronutrient requirements across portions (plant-source foods)**

**
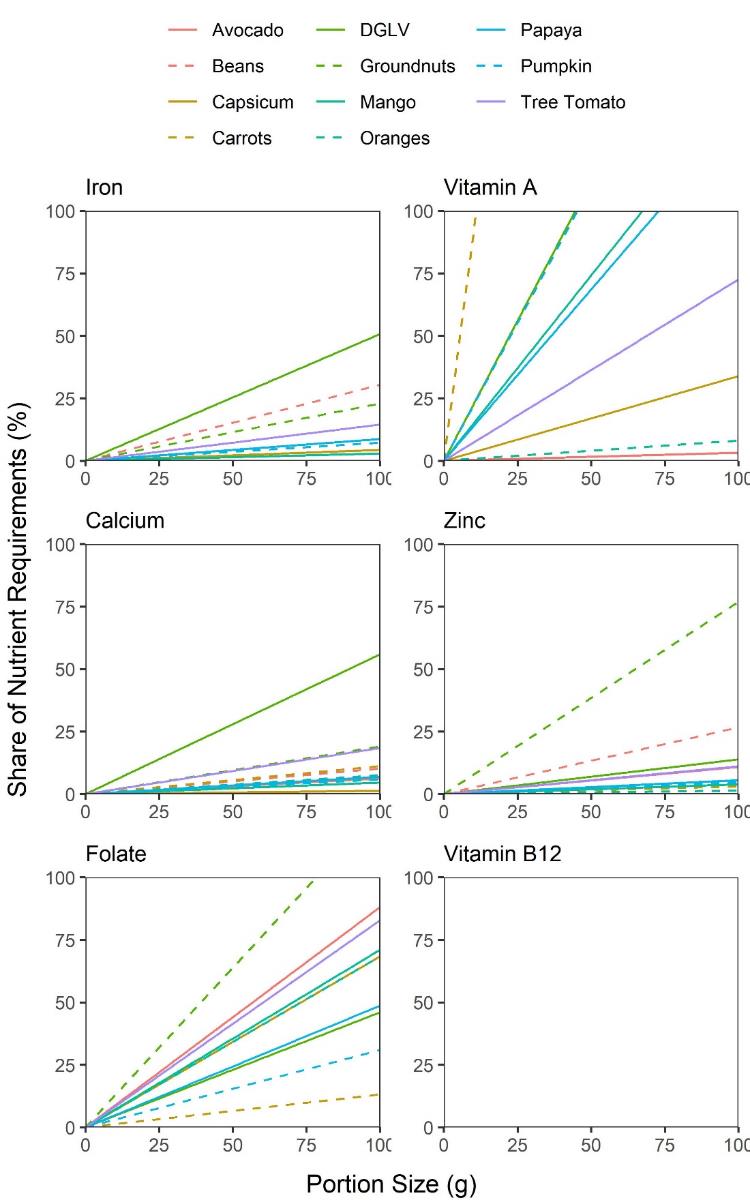
**

DGLV = dark green leafy vegetables

**Appendix Figure 2: Average share of single micronutrient requirements across portions (animal-source foods)**

**
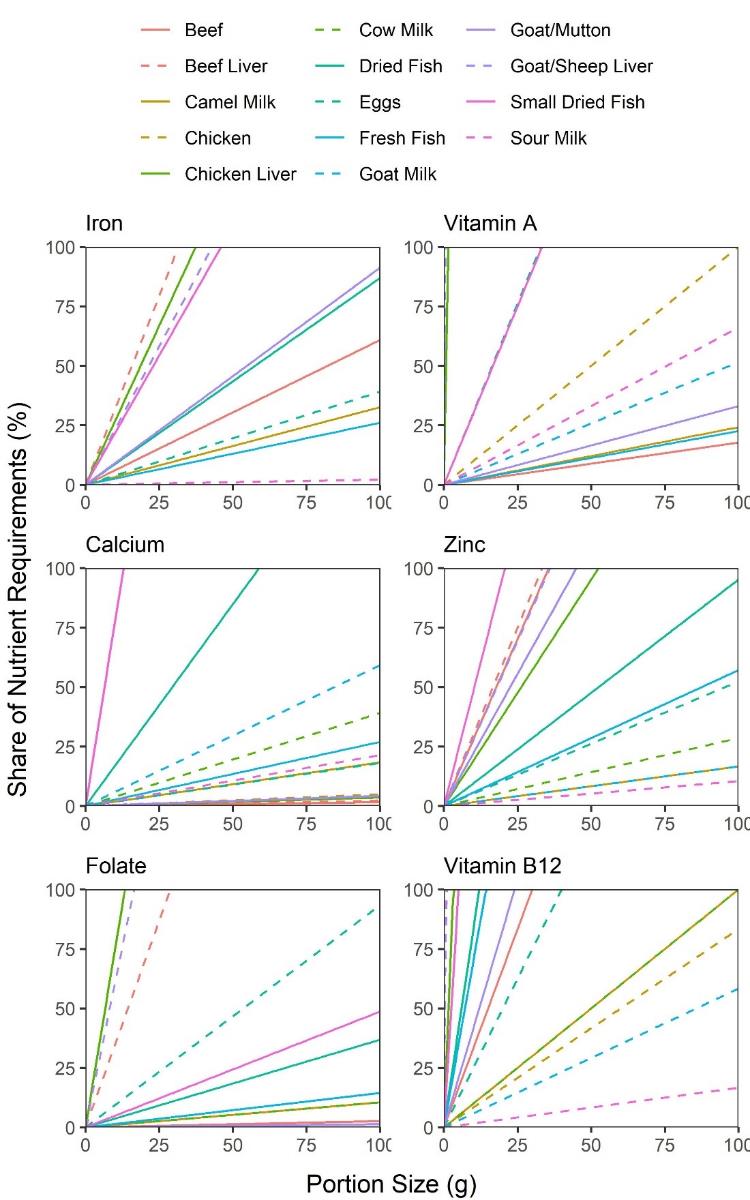
**

**Appendix Figure 3: Average share of micronutrient requirements across portion sizes and foods**

**
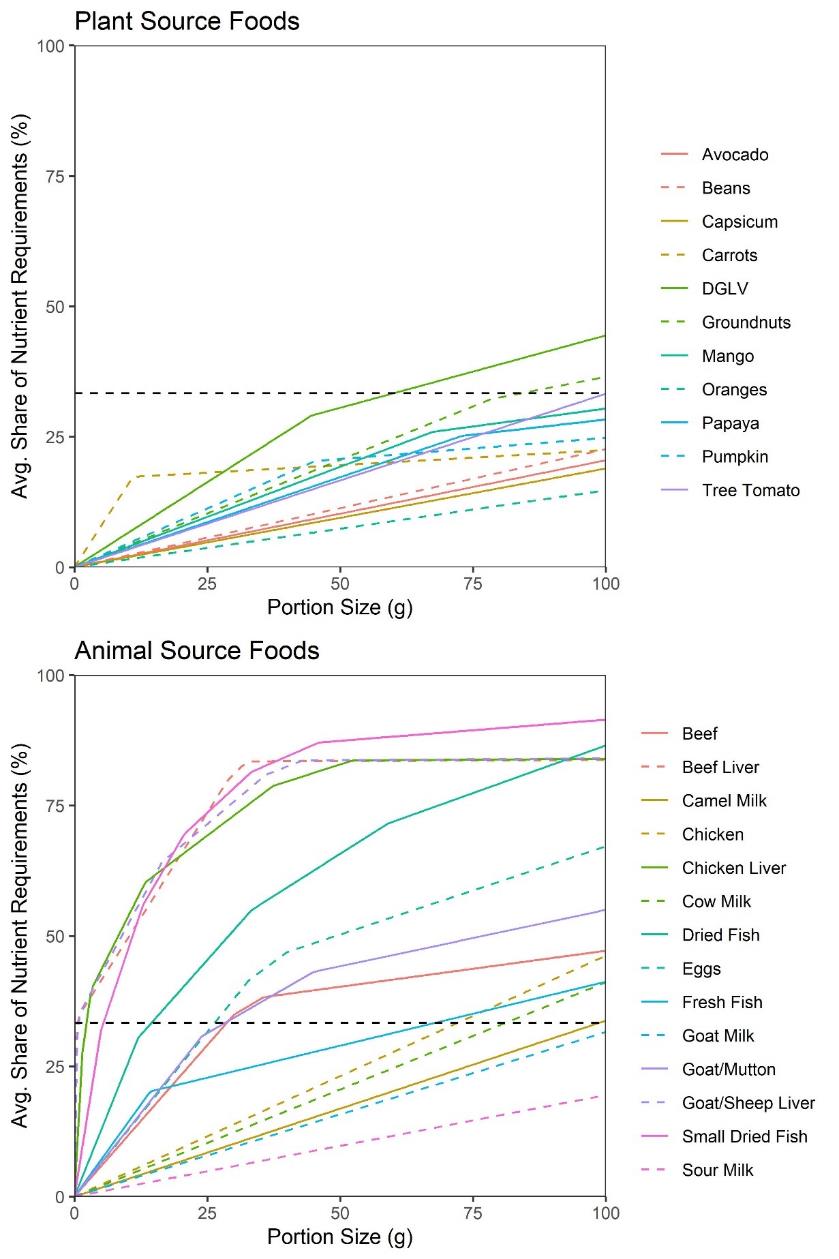
**

DGLV = dark green leafy vegetables

**Supplementary Methods – Affordability thresholds**

In previous analysis (Ryckman, Beal, Nordhagen, Chimanya, & Matji, 2021), we also assessed absolute affordability of foods and nutrients by comparing the cost of foods per adjusted household food expenditure against affordability thresholds of 10% and 33.3% for the single and joint nutrient analyses, respectively. In those studies, we restricted the analysis to only households with children of complementary feeding age since these households tended to have somewhat lower adjusted food expenditures (i.e., fewer resources to shift food consumption) than the average household. However, provincial sample sizes in the KIHBS are small enough that further restricting the sample of surveyed households would prevent the results from being provincially representative. Instead, we adjusted the affordability thresholds of 10% and 33.3% based on the difference between adjusted household food expenditure for the average surveyed household and for the average surveyed household with a child under two. This adjustment yielded revised affordability thresholds of 7.6% for the single nutrient analysis and 25.2% for the joint analysis.

**Appendix References**

Bognar, A. (2002). Tables on weight yield of food and retention factors of food constituents for the calculation of nutrient composition of cooked foods (dishes) (No. BFE-R-- 02-03). Karlsruhe: Bundesforschungsanstalt für Ernährung. Retrieved from Bundesforschungsanstalt für Ernährung website: http://www.fao.org/uploads/media/bognar_bfe-r-02-03.pdf

Dewey, K. G. (2001). Nutrition, Growth, and Complementary Feeding of The Brestfed Infant. Pediatric Clinics of North America, 48(1), 87–104. https://doi.org/10.1016/S0031-3955(05)70287-X

Kabahenda, M. K., Amega, R., Okalany, E., Husken, S. M. C., & Heck, S. (2011). Protein and micronutrient composition of low value fish products commonly marketed in the Lake Victoria region. World Journal of Agricultural Sciences. Retrieved from https://digitalarchive.worldfishcenter.org/handle/20.500.12348/1196

Korkalo, L., Hauta-alus, H., & Mutanen, M. (2011). Food composition tables for Mozambique: Version 2. Helsinki, Finland: Department of Food and Environmental Sciences, University of Helsinki. Retrieved from Department of Food and Environmental Sciences, University of Helsinki website: https://researchportal.helsinki.fi/en/publications/food-composition-tables-for-mozambique-version-2

Lukmanji, Z., Hertzmark, E., Mlingi, N., Assey, V., Ndossi, G., & Fawzi, W. (2008). Tanzania Food Composition Tables. Dar es Salaam, Tanzania: Muhimbili University of Health and Allied Sciences (MUHAS), Tanzania Food and Nutrition Centre (TFNC), Harvard School of Public Health (HSPH). Retrieved from Muhimbili University of Health and Allied Sciences (MUHAS), Tanzania Food and Nutrition Centre (TFNC), Harvard School of Public Health (HSPH) website: https://cdn1.sph.harvard.edu/wp-content/uploads/sites/30/2012/10/tanzania-food-composition-tables.pdf

Mwai, J., Kimani, A., Mbelenga, E., Charrondiere, U. R., Grande, F., Rittenschober, D., … Murugu, D. (2018). Kenya Food Composition Tables. FAO/Government of Kenya.

Nyirenda, D. B., Musukwa, M., Mugode, R. H., & Shindano, J. (2009). Zambia Food Composition Tables, 4th edition. Lusaka, Zambia: National Food and Nutrition Commission. Retrieved from National Food and Nutrition Commission website: http://nfnc.org.zm/download/file/fid/537

Roseland, J. M., Nguyen, Q. A., Williams, J. R., Patterson, K. Y., Showell, B., & Pehrsson, P. R. (2014). USDA Table of Cooking Yields for Meat and Poultry. USDA Agricultural Research Service. Retrieved from https://data.nal.usda.gov/dataset/usda-table-cooking-yields-meat-and-poultry

Ryckman, T., Beal, T., Nordhagen, S., Chimanya, K., & Matji, J. (2021). Affordability of nutritious foods for complementary feeding in Eastern and Southern Africa. Nutrition Reviews, 79(Supplement_4). http://dx.doi.org/10.1093/nutrit/nuaa137

Stadlymayr, B., Charrondiere, U. R., Enujiugha, V. N., Bayili, R. G., Fagbohoun, E. G., Samb, B., … Barikmo, I. (2012). West African food composition table/table De composition Des Aliments D’afrique De L’ouest. Rome, Italy: Food and Agricultural Organization of the United Nations. Retrieved from Food and Agricultural Organization of the United Nations website: http://www.fao.org/3/a-i2698b.pdf

Steiner‐Asiedu, M., Lied, E., Lie, Ø., Nilsen, R., & Julshamn, K. (1993). The nutritive value of sun-dried pelagic fish from the rift valley in Africa. Journal of the Science of Food and Agriculture, 63(4), 439–443. https://doi.org/10.1002/jsfa.2740630410

U.S. Department of Agriculture, Agricultural Research Service. (2019). FoodData Central. Retrieved January 26, 2020, from https://fdc.nal.usda.gov./

World Health Organization, & Food and Agriculture Organization of the United Nations (Eds.). (2004). Vitamin and mineral requirements in human nutrition (2nd ed). Geneva : Rome: World Health Organization ; FAO. Retrieved from https://apps.who.int/iris/bitstream/handle/10665/42716/9241546123.pdf

**Appendix Figure 4: Current household consumption, by county and province**

**
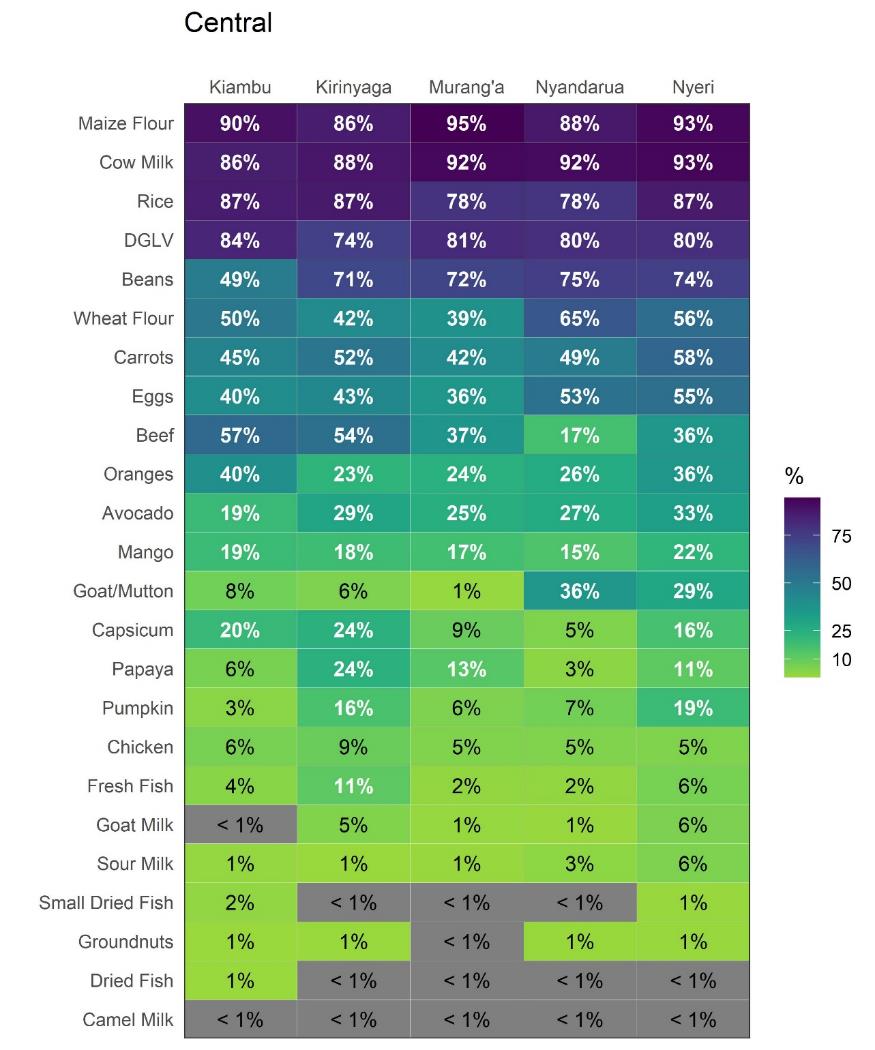
**

Graph shows the proportion of households that consumed different foods in the week prior to being surveyed. Foods with ≥ 10% consumption, shown in white, were selected for the affordability analysis for each province and nationally.

**
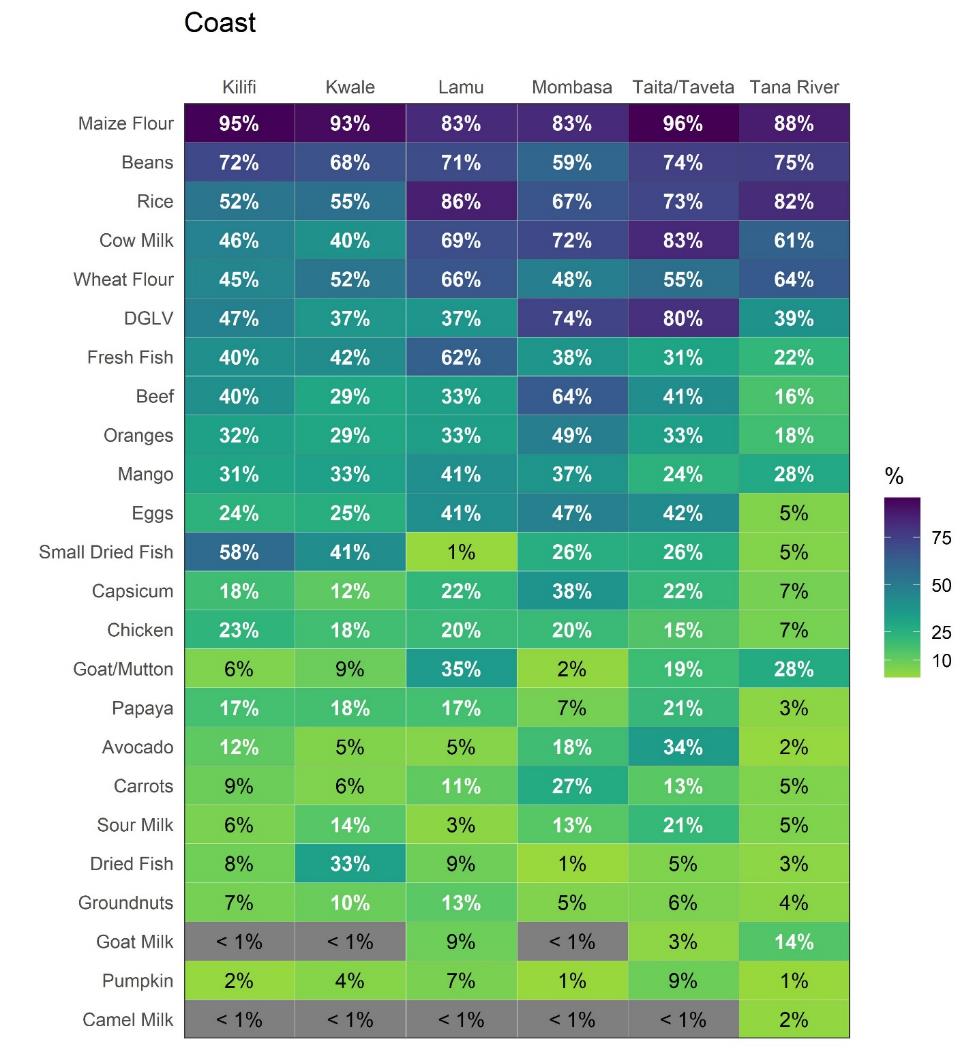
**

Graph shows the proportion of households that consumed different foods in the week prior to being surveyed. Foods with ≥ 10% consumption, shown in white, were selected for the affordability analysis for each province and nationally.

**
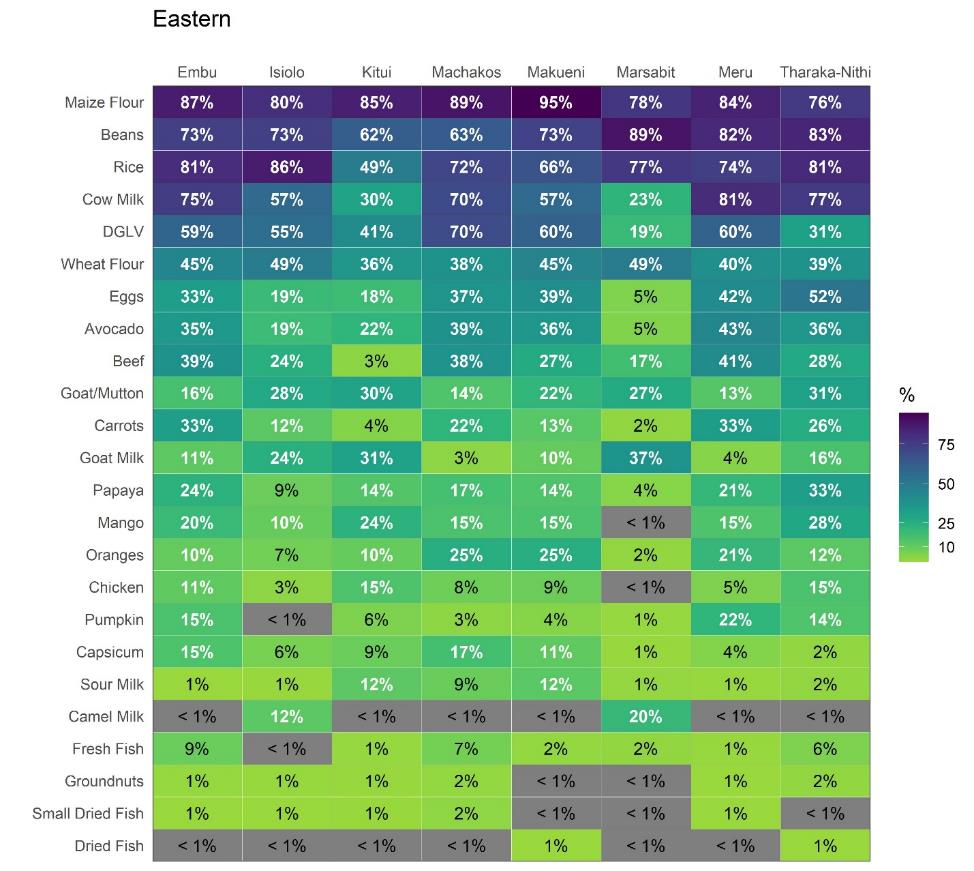
**

Graph shows the proportion of households that consumed different foods in the week prior to being surveyed. Foods with ≥ 10% consumption, shown in white, were selected for the affordability analysis for each province and nationally.

**
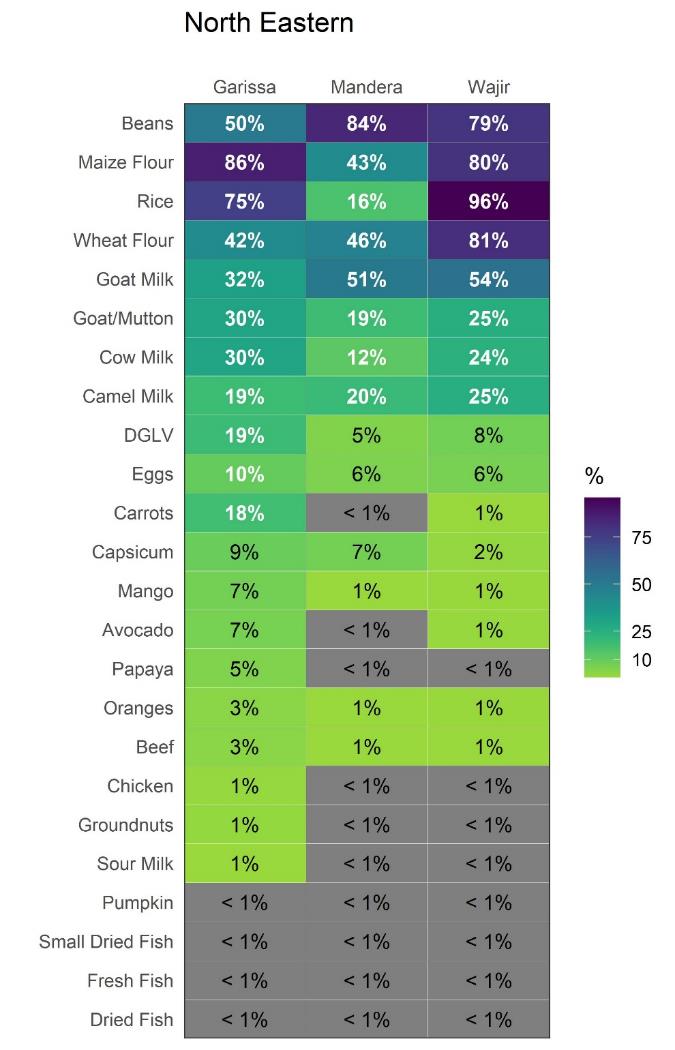
**

Graph shows the proportion of households that consumed different foods in the week prior to being surveyed. Foods with ≥ 10% consumption, shown in white, were selected for the affordability analysis for each province and nationally.

**
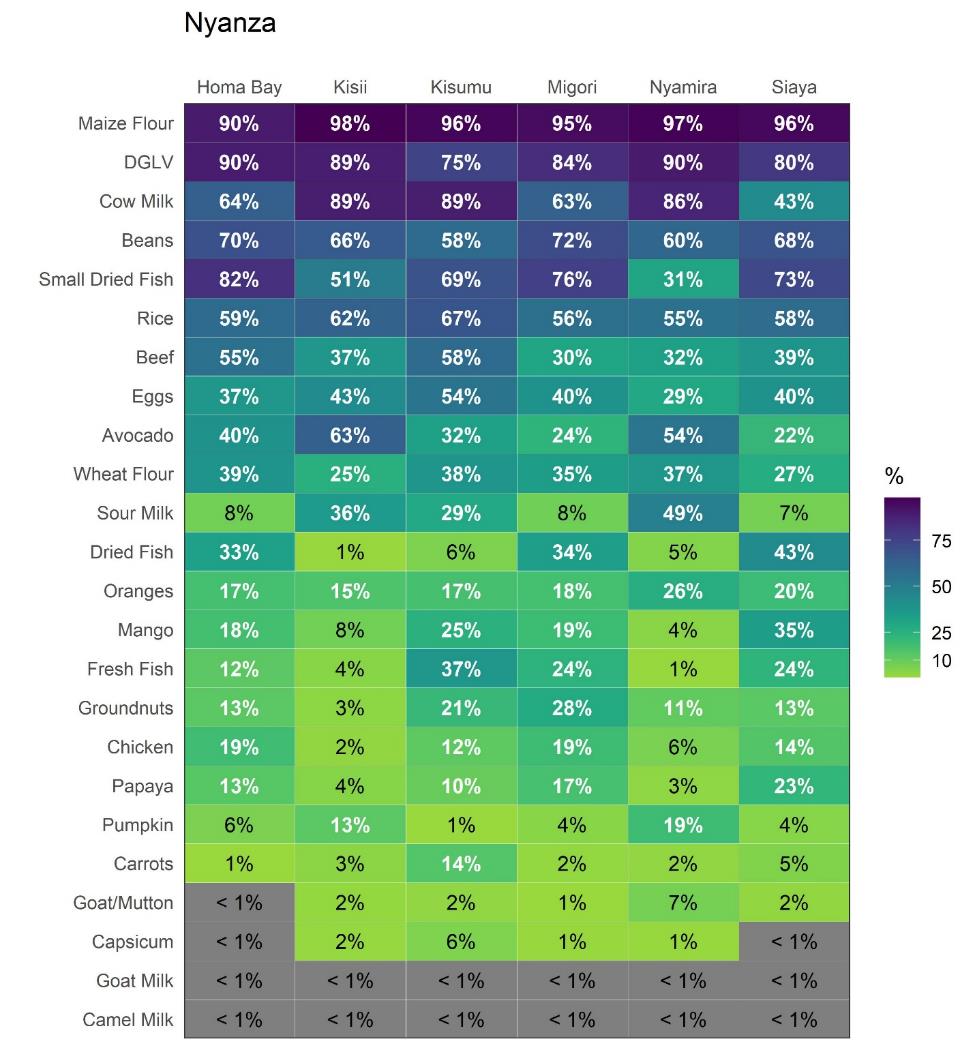
**

Graph shows the proportion of households that consumed different foods in the week prior to being surveyed. Foods with ≥ 10% consumption, shown in white, were selected for the affordability analysis for each province and nationally.

**
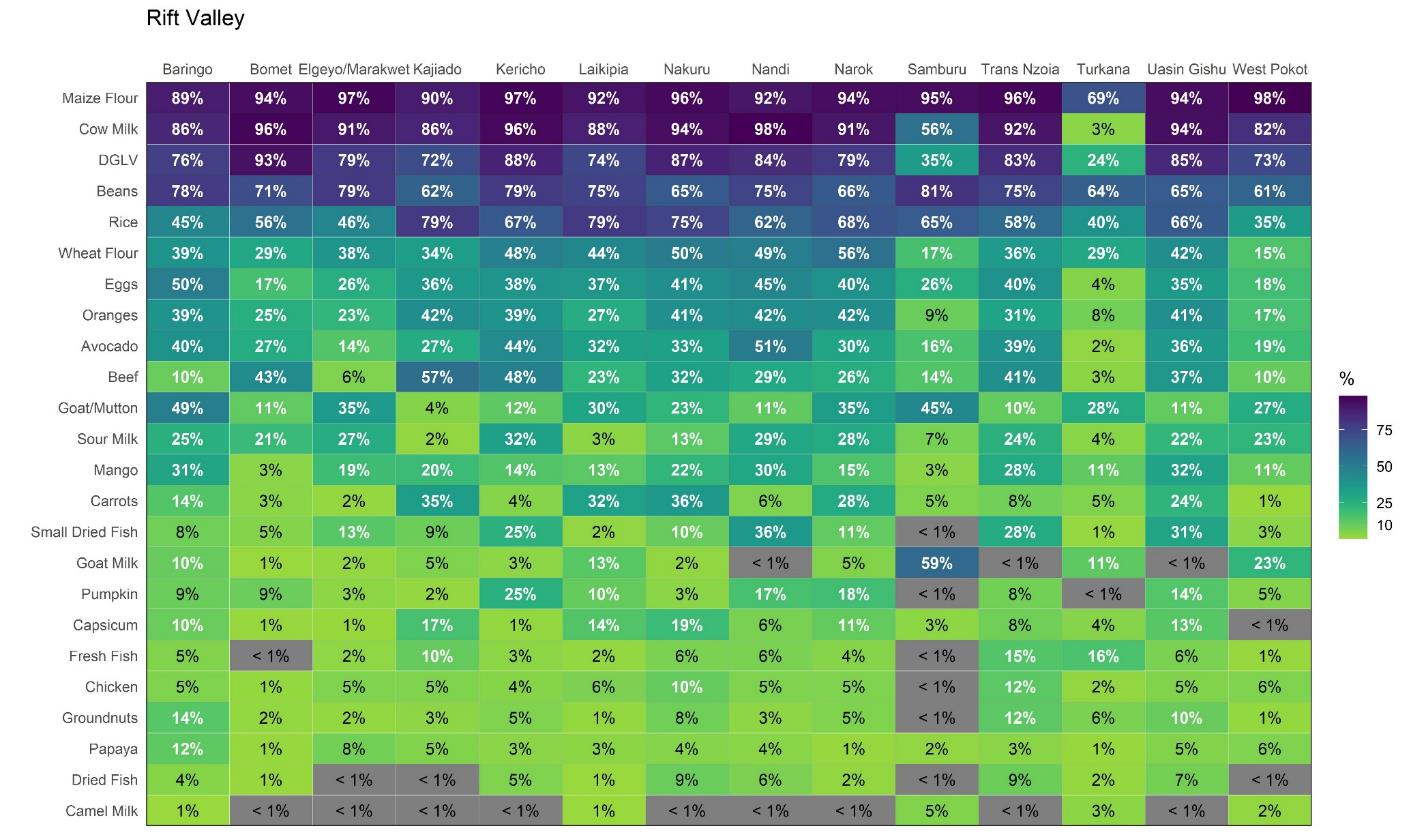
**

Graph shows the proportion of households that consumed different foods in the week prior to being surveyed. Foods with ≥ 10% consumption, shown in white, were selected for the affordability analysis for each province and nationally.

**
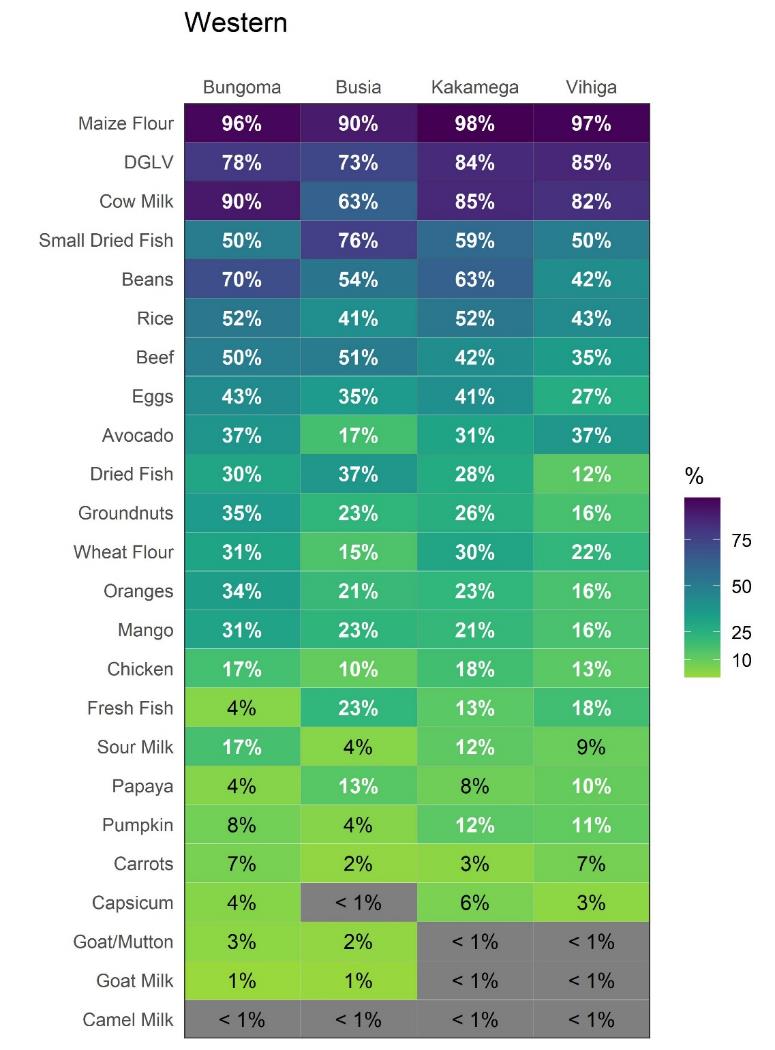
**

Graph shows the proportion of households that consumed different foods in the week prior to being surveyed. Foods with ≥ 10% consumption, shown in white, were selected for the affordability analysis for each province and nationally.

**Appendix Figure 5: Food and nutrient affordability nationally and by province**

**
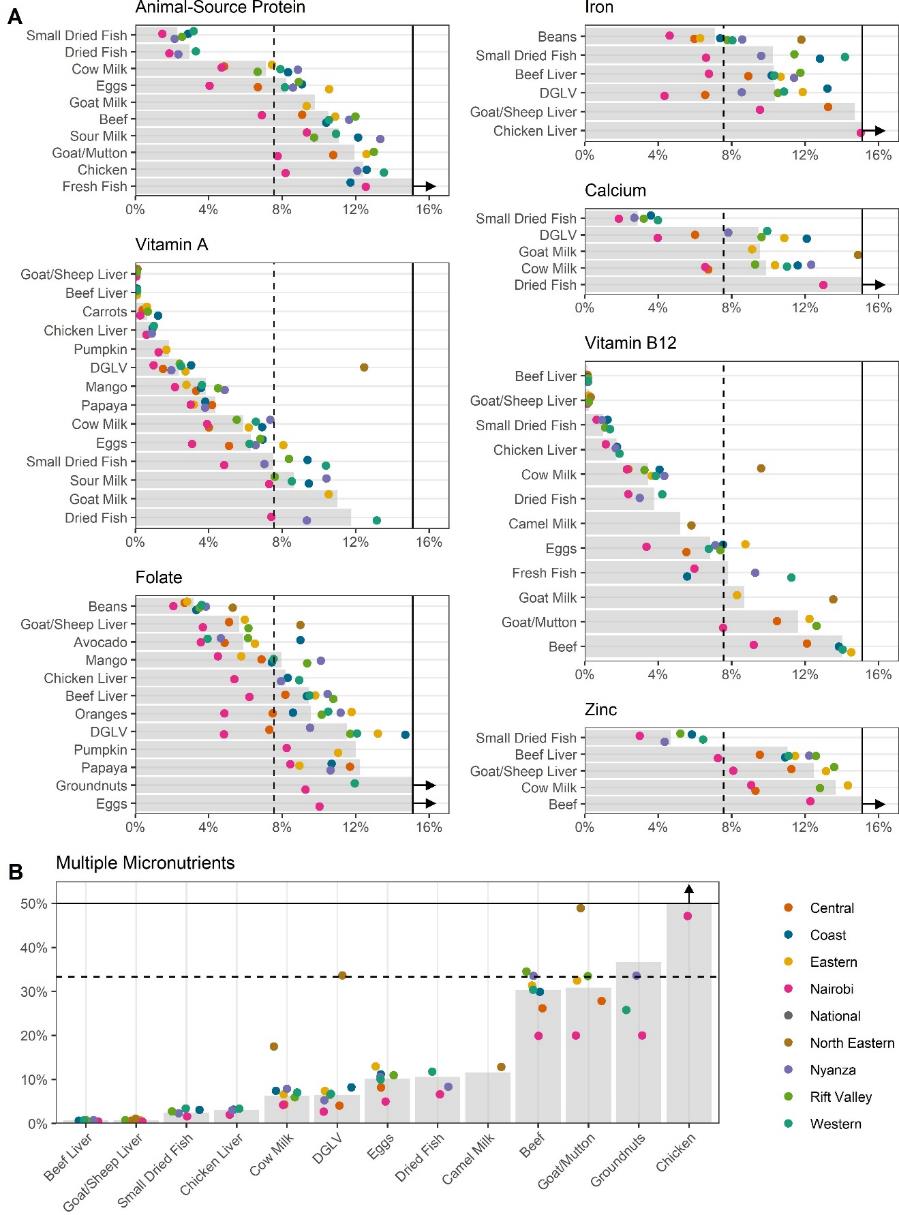
**

Dots show cost as a share of adjusted food expenditure for each province, while grey bars shown national values. Values were truncated at 15% (single nutrients) and 50% (multiple nutrients). The dashed lines indicate the single and multiple nutrient affordability thresholds of 7.6% and 25.2%; bars below these thresholds are considered affordable for the average household.

**Appendix Figure 6: Cost of food portions that could meet half of single nutrient requirements, as a share of adjusted household food expenditures, by province**

**
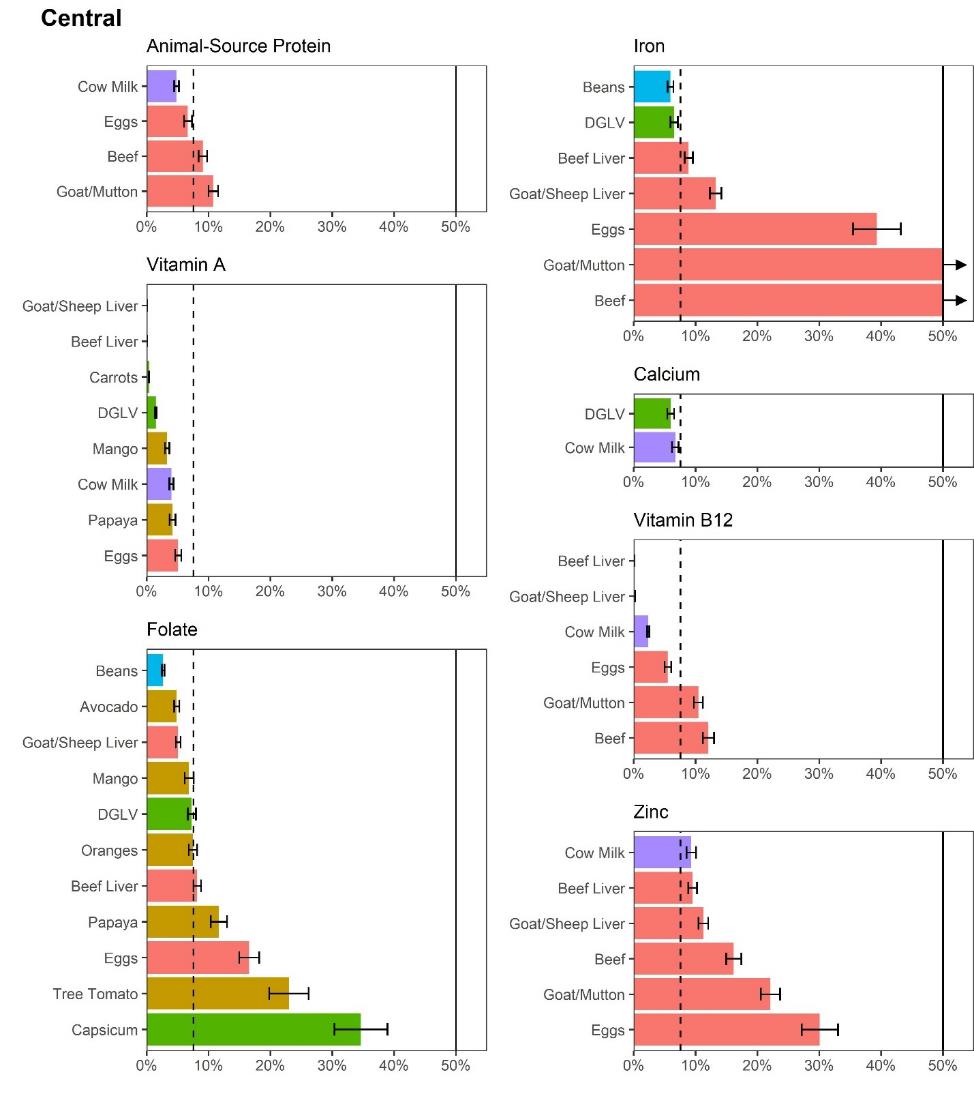
**

Bars were truncated at 50%; arrows indicate that the true values extend beyond 50%. The dashed lines indicate the adjusted single nutrient affordability threshold of 7.6%; bars below that threshold are considered affordable for the average household. Error bars indicate 95% CIs on the means.

**
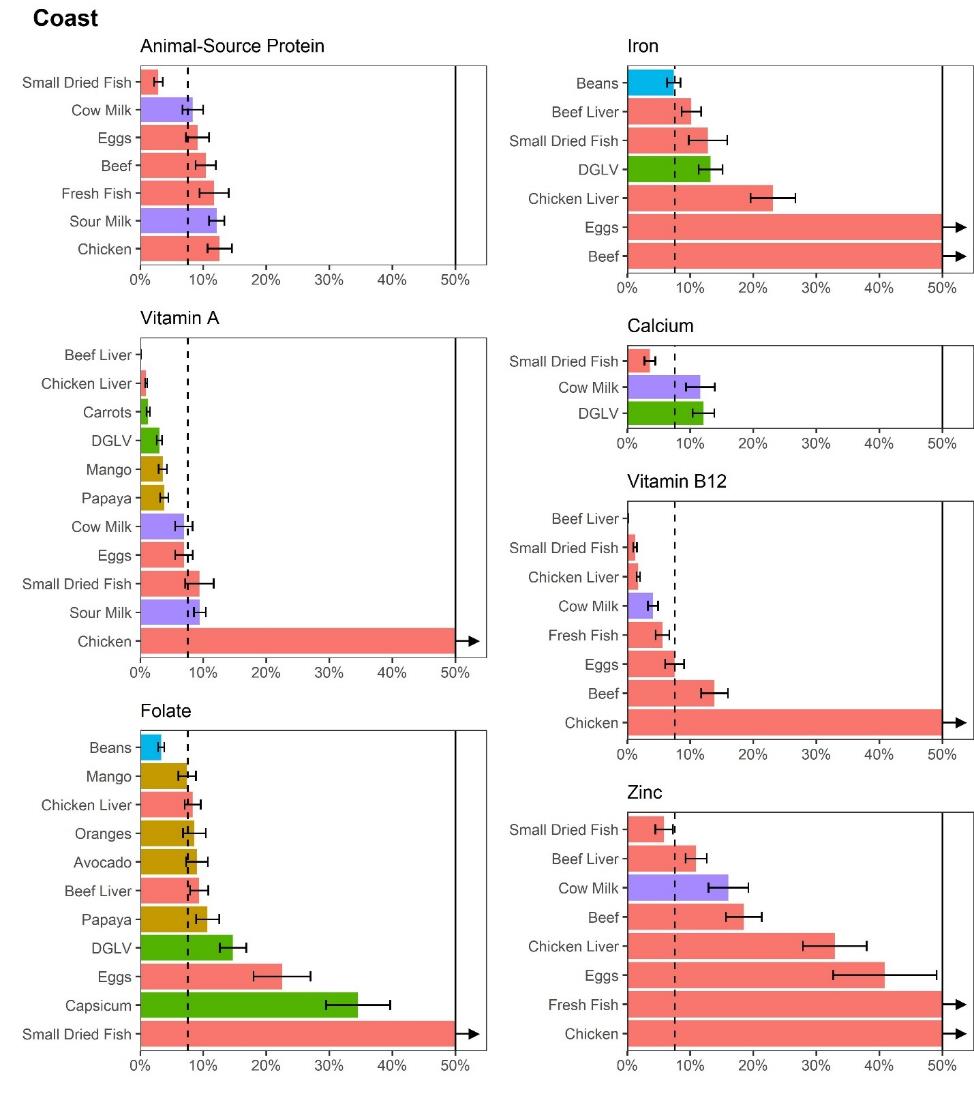
**

Bars were truncated at 50%; arrows indicate that the true values extend beyond 50%. The dashed lines indicate the adjusted single nutrient affordability threshold of 7.6%; bars below that threshold are considered affordable for the average household. Error bars indicate 95% CIs on the means.

**
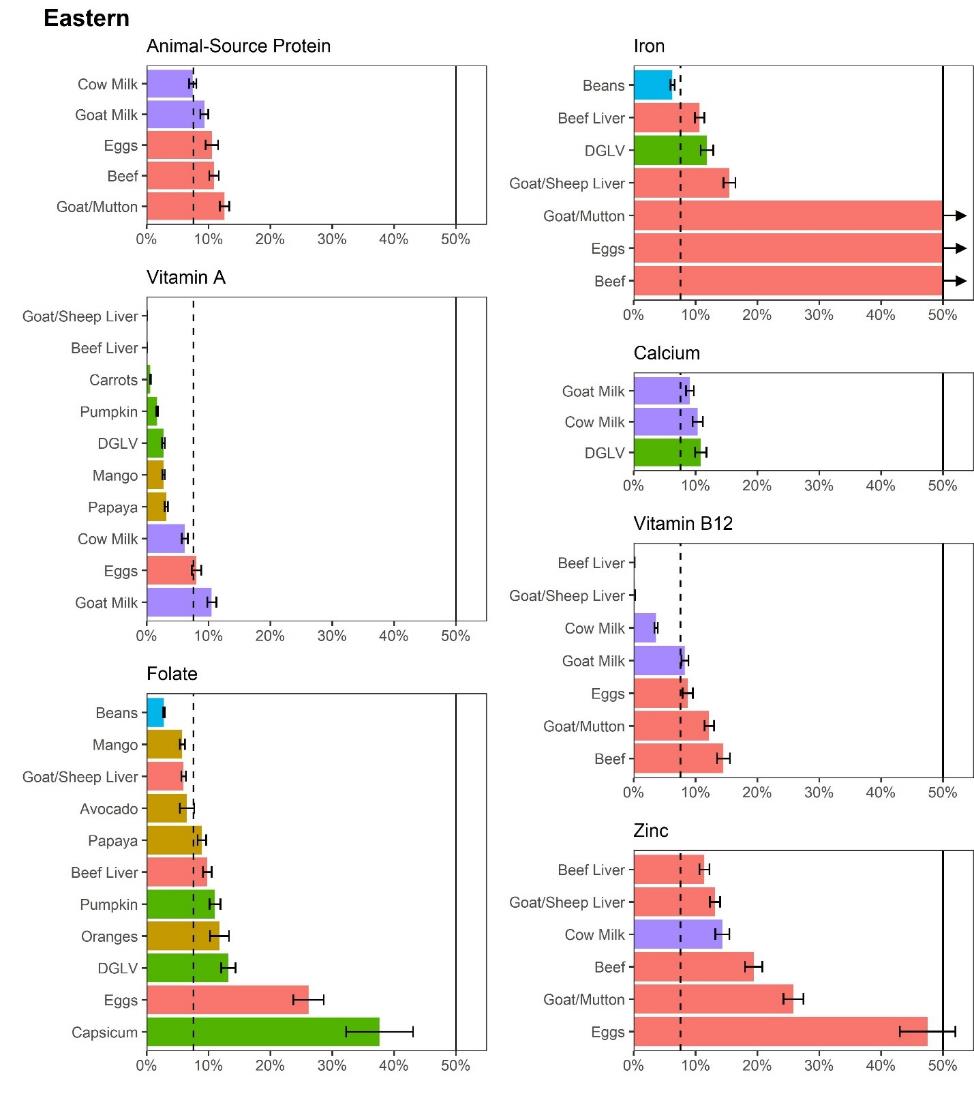
**

Bars were truncated at 50%; arrows indicate that the true values extend beyond 50%. The dashed lines indicate the adjusted single nutrient affordability threshold of 7.6%; bars below that threshold are considered affordable for the average household. Error bars indicate 95% CIs on the means.

**
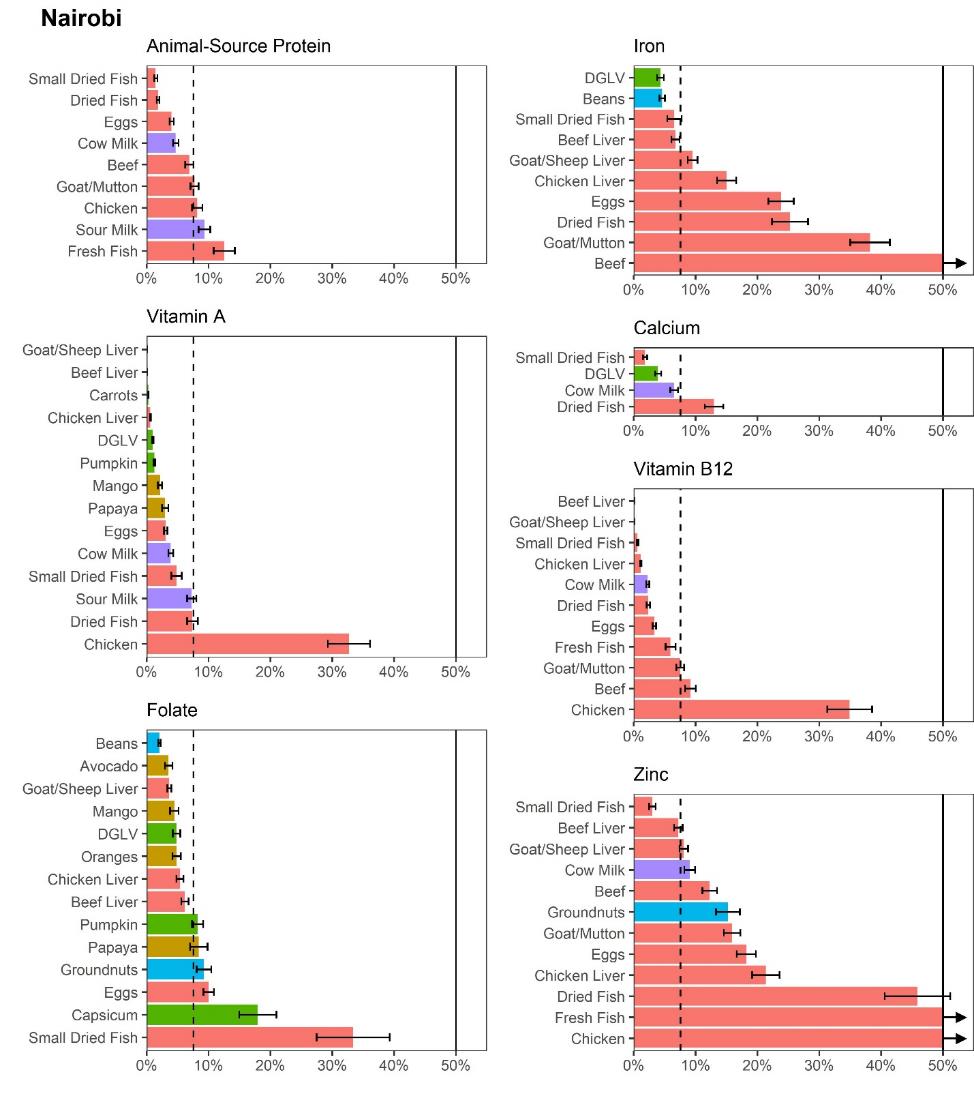
**

Bars were truncated at 50%; arrows indicate that the true values extend beyond 50%. The dashed lines indicate the adjusted single nutrient affordability threshold of 7.6%; bars below that threshold are considered affordable for the average household. Error bars indicate 95% CIs on the means.

**
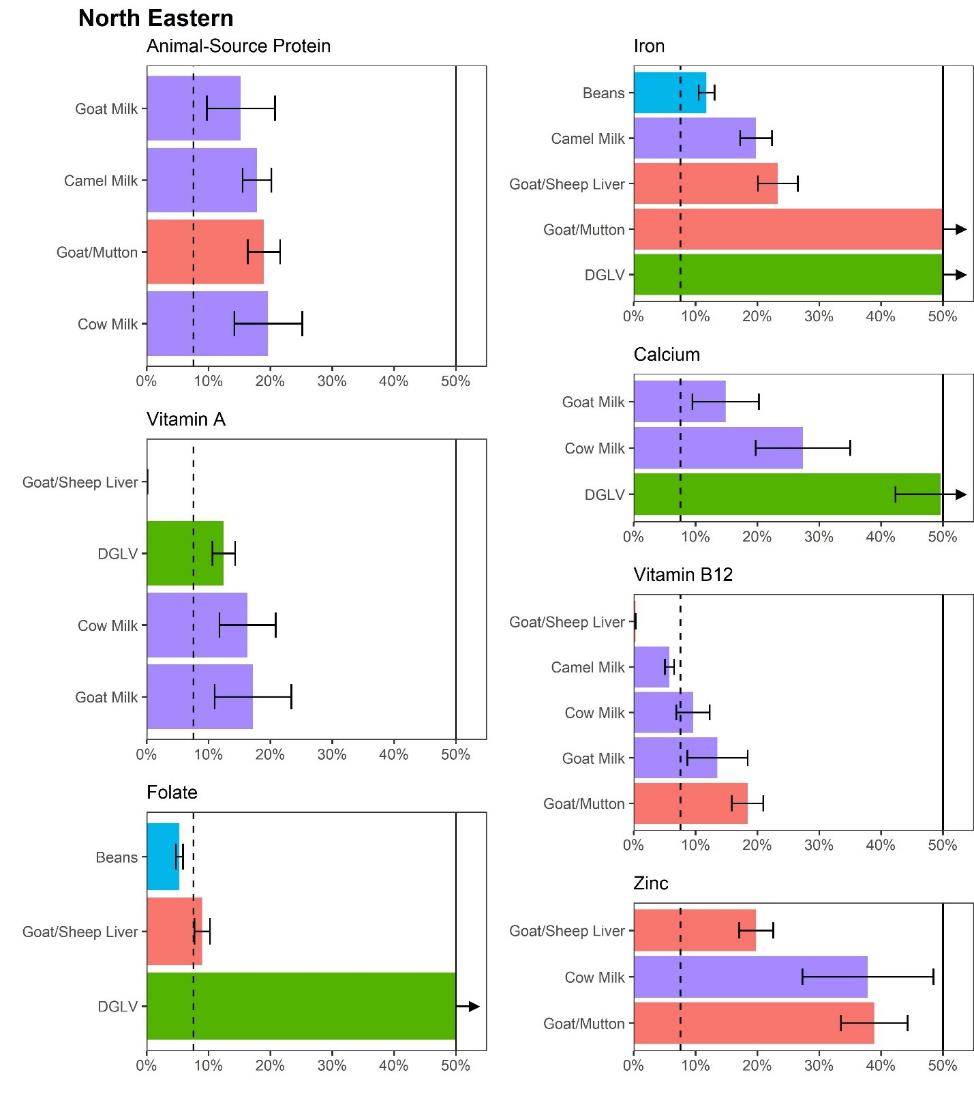
**

Bars were truncated at 50%; arrows indicate that the true values extend beyond 50%. The dashed lines indicate the adjusted single nutrient affordability threshold of 7.6%; bars below that threshold are considered affordable for the average household. Error bars indicate 95% CIs on the means.


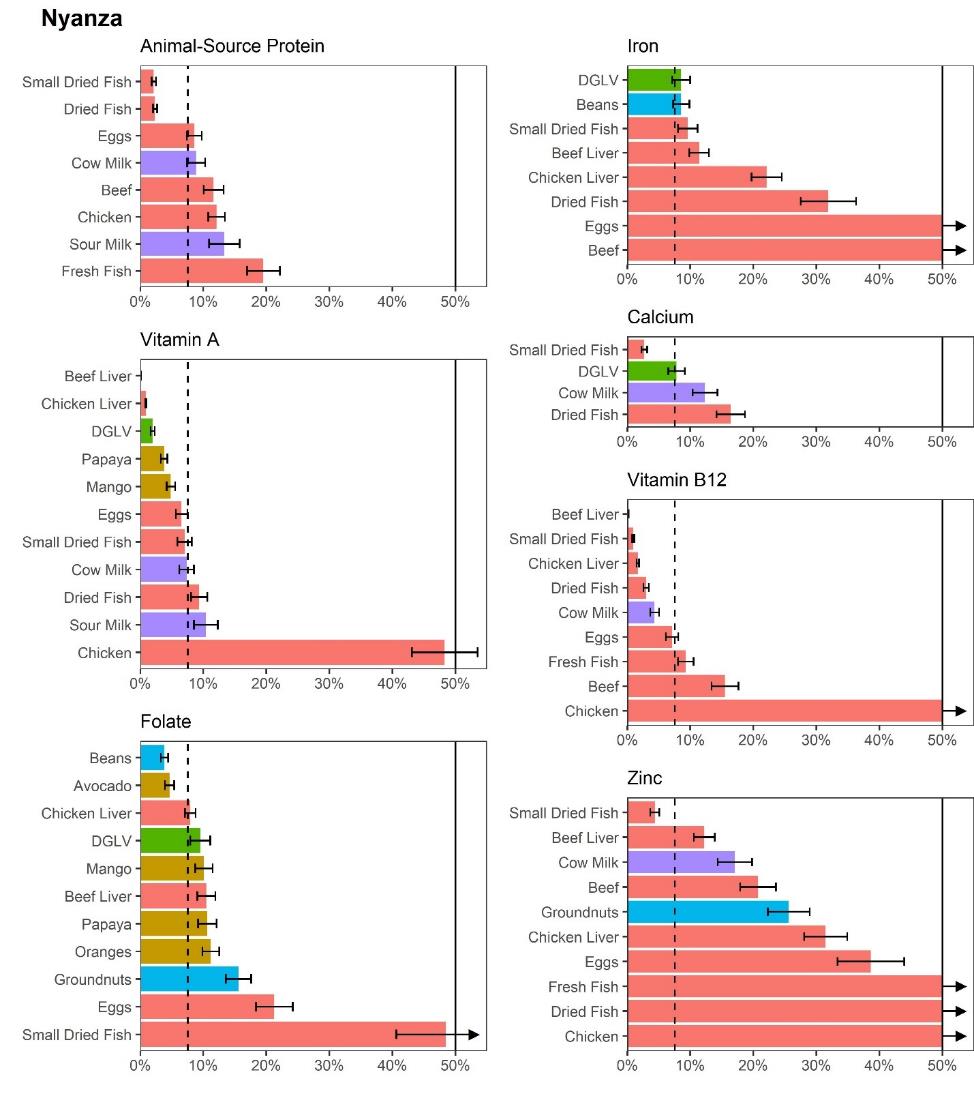


Bars were truncated at 50%; arrows indicate that the true values extend beyond 50%. The dashed lines indicate the adjusted single nutrient affordability threshold of 7.6%; bars below that threshold are considered affordable for the average household. Error bars indicate 95% CIs on the means.


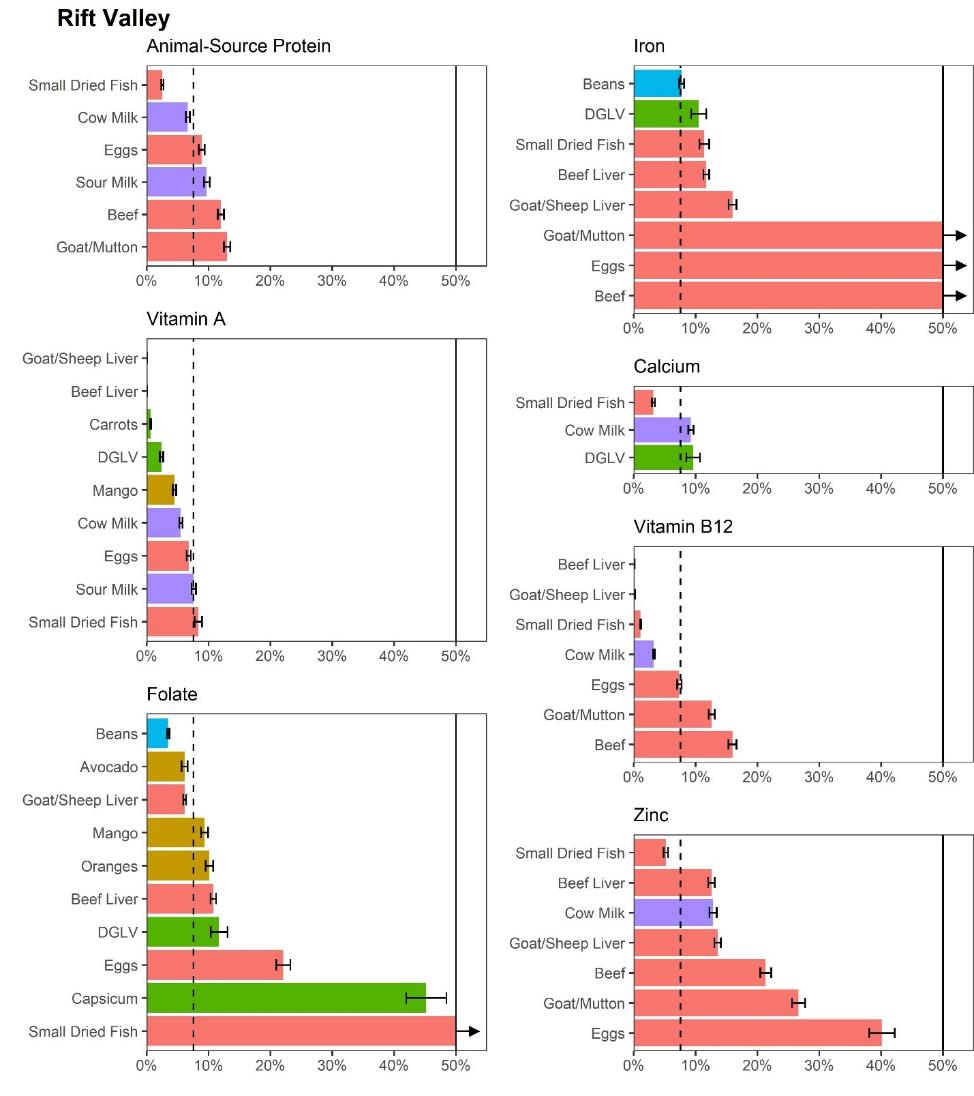


Bars were truncated at 50%; arrows indicate that the true values extend beyond 50%. The dashed lines indicate the adjusted single nutrient affordability threshold of 7.6%; bars below that threshold are considered affordable for the average household. Error bars indicate 95% CIs on the means.


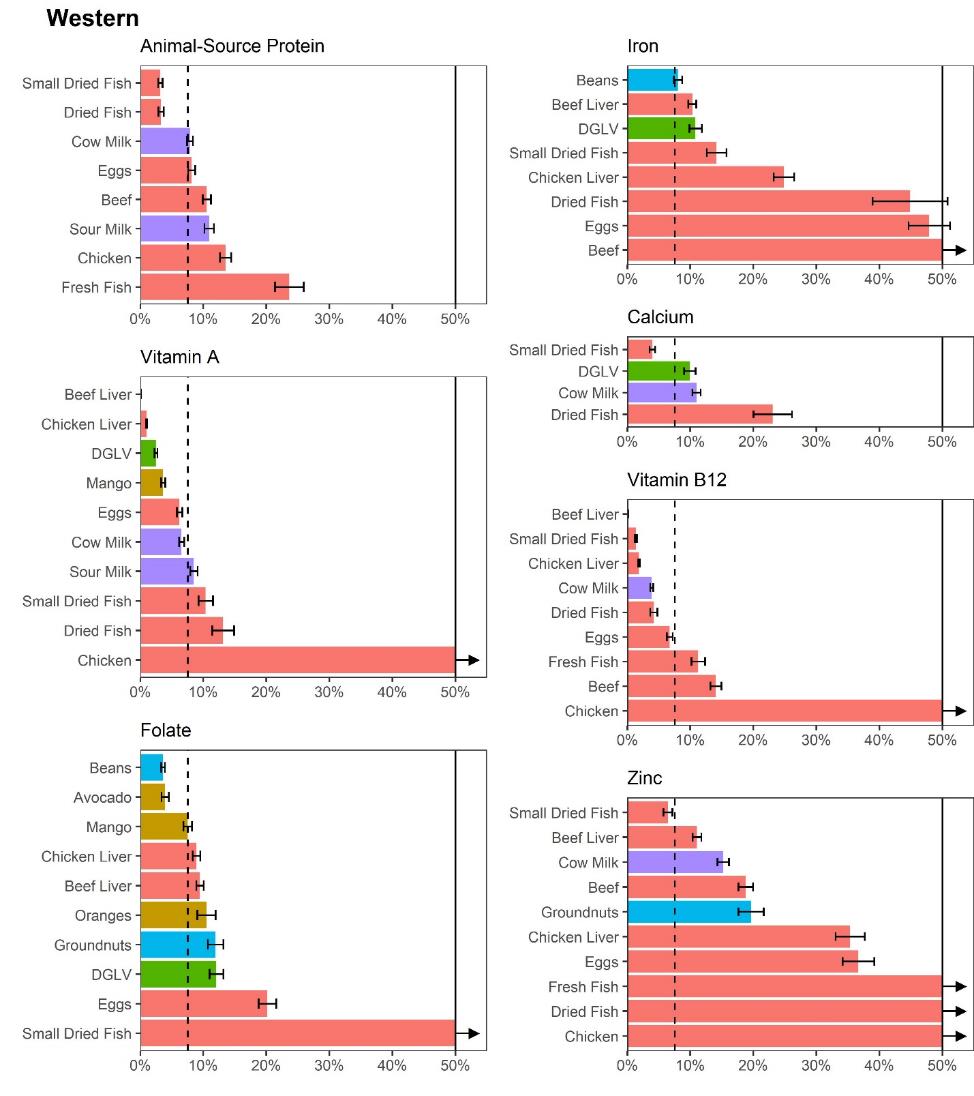


Bars were truncated at 50%; arrows indicate that the true values extend beyond 50%. The dashed lines indicate the adjusted single nutrient affordability threshold of 7.6%; bars below that threshold are considered affordable for the average household. Error bars indicate 95% CIs on the means.

**Appendix Figure 7: Percent of households that can afford foods and nutrients at various thresholds, nationally and by province**

**
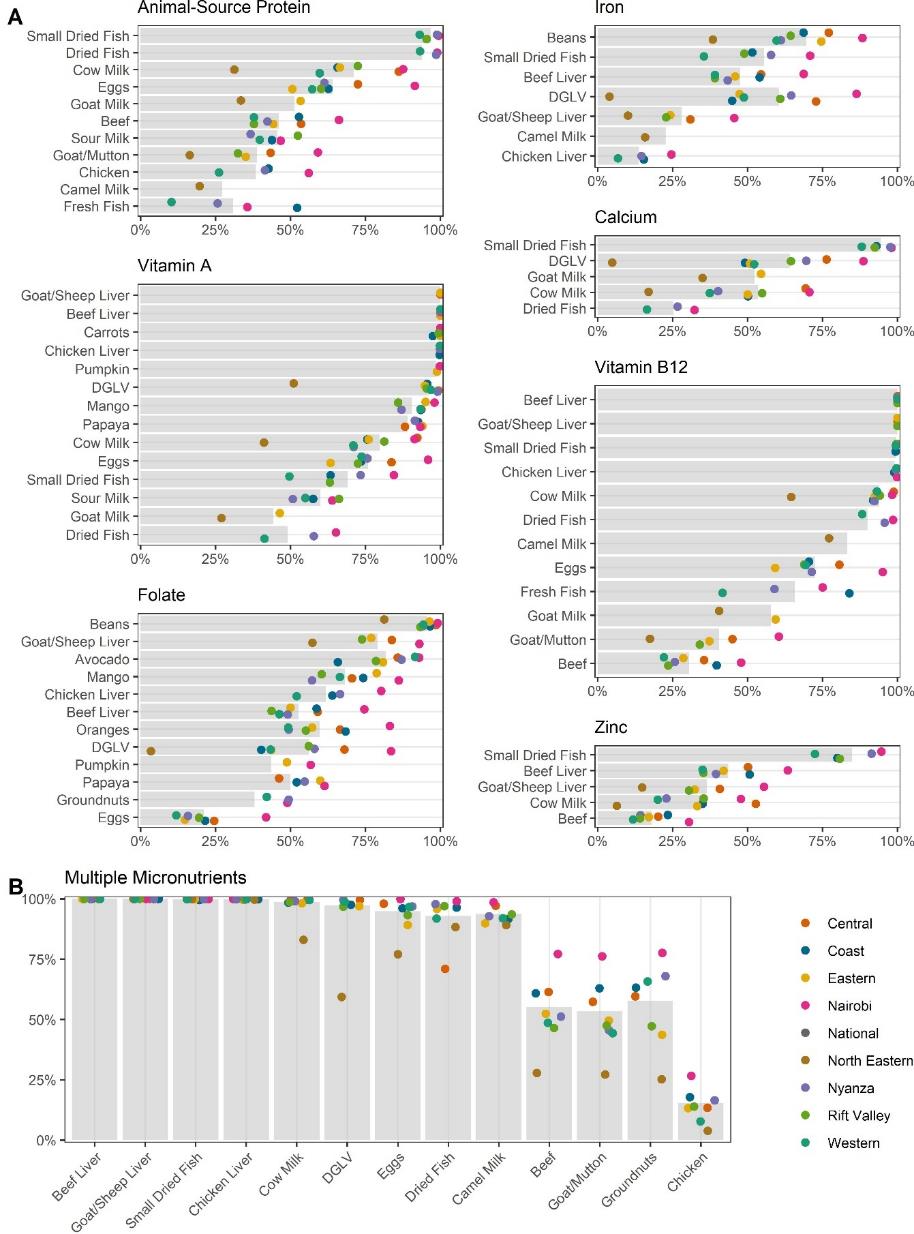
**

Figure shows the percentage of households nationally (grey bars) and provincially (colored markers) that can afford each food and nutrient at the base affordability thresholds (7.6% for single nutrient analysis and 25.2% for joint nutrient analysis).

**Appendix Figure 8: Percent of households that can afford foods and single nutrients at various affordability thresholds, by province**


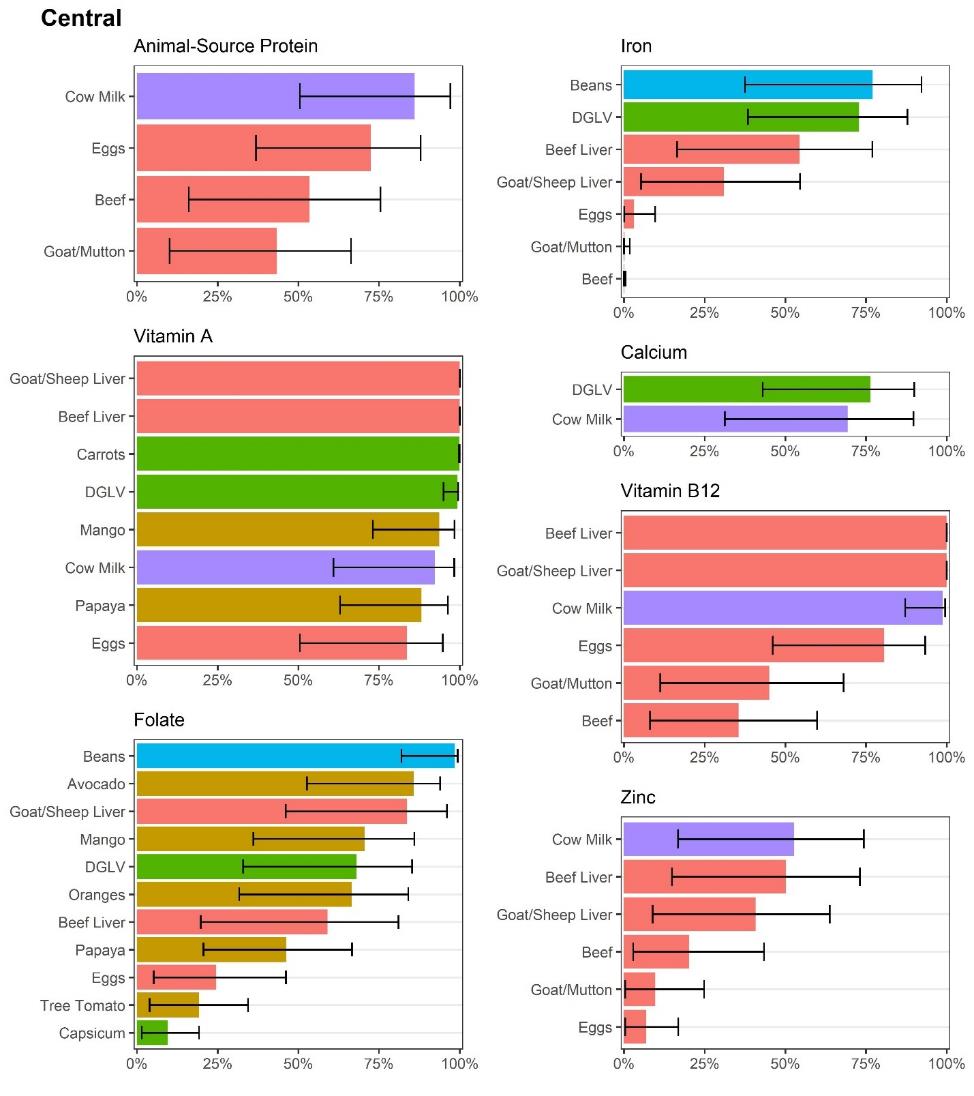


Figure shows the percentage of households that can afford each single food and nutrient at the base affordability thresholds (filled bars; 7.6%), lower thresholds (lower error bars; half the base thresholds, requiring that foods cost less to be considered affordable), and higher thresholds (upper error bars; 50% higher than the base thresholds, allowing foods to cost more and still be considered affordable).


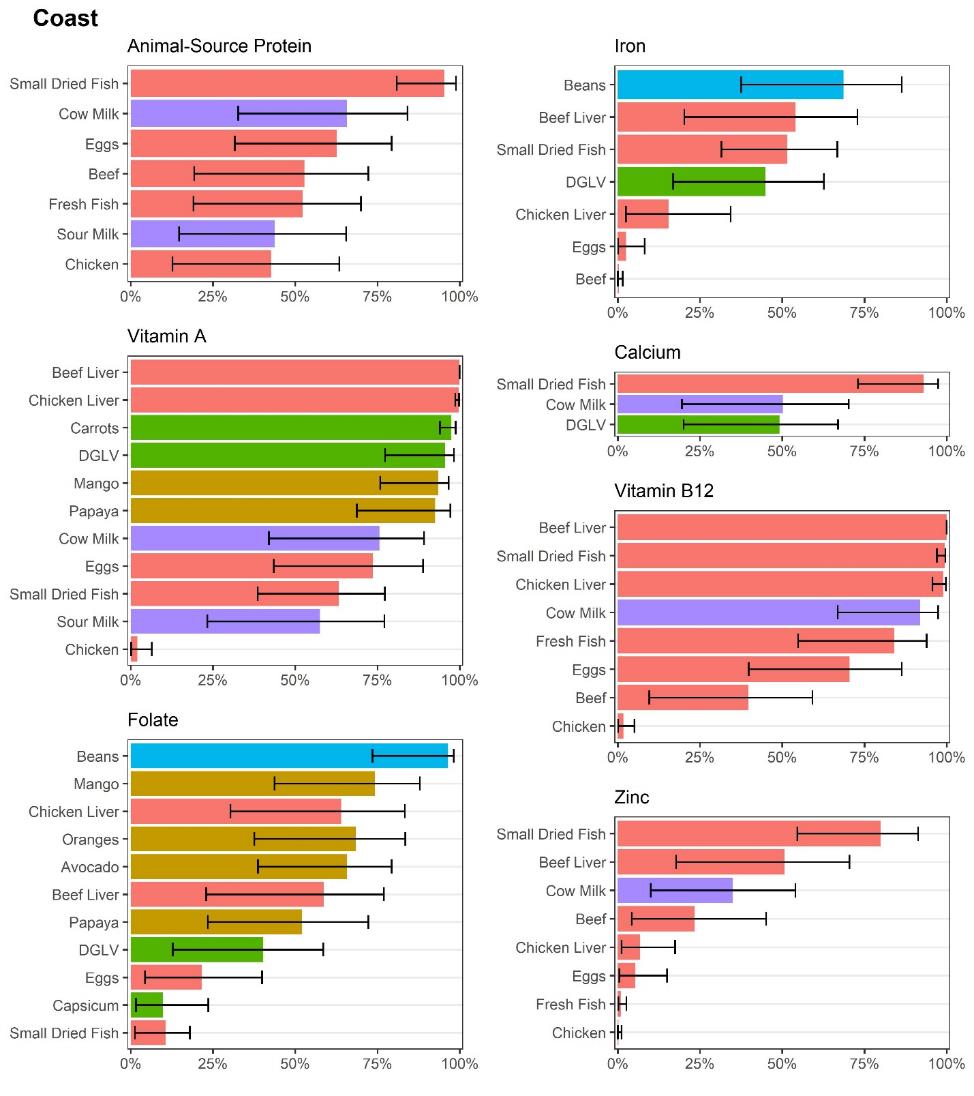


Figure shows the percentage of households that can afford each single food and nutrient at the base affordability thresholds (filled bars; 7.6%), lower thresholds (lower error bars; half the base thresholds, requiring that foods cost less to be considered affordable), and higher thresholds (upper error bars; 50% higher than the base thresholds, allowing foods to cost more and still be considered affordable).


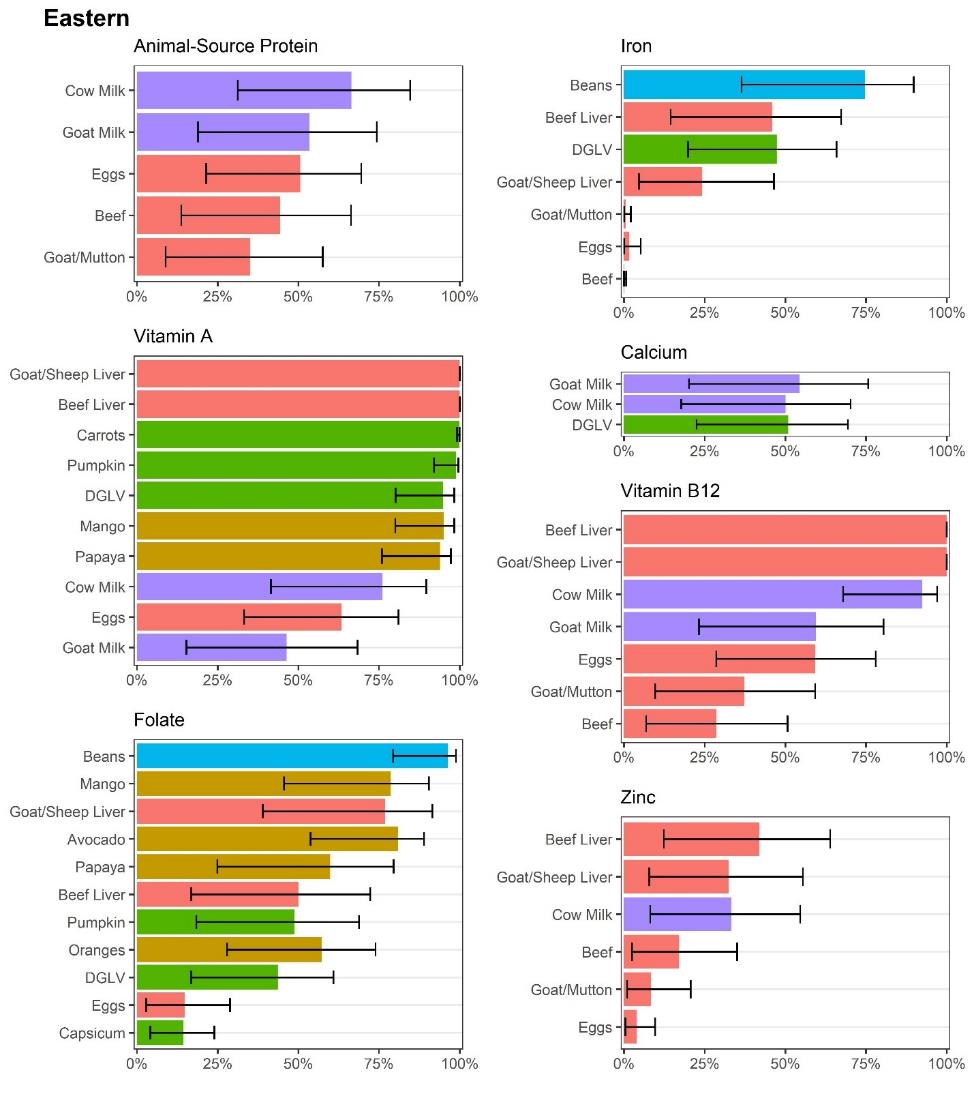


Figure shows the percentage of households that can afford each single food and nutrient at the base affordability thresholds (filled bars; 7.6%), lower thresholds (lower error bars; half the base thresholds, requiring that foods cost less to be considered affordable), and higher thresholds (upper error bars; 50% higher than the base thresholds, allowing foods to cost more and still be considered affordable).


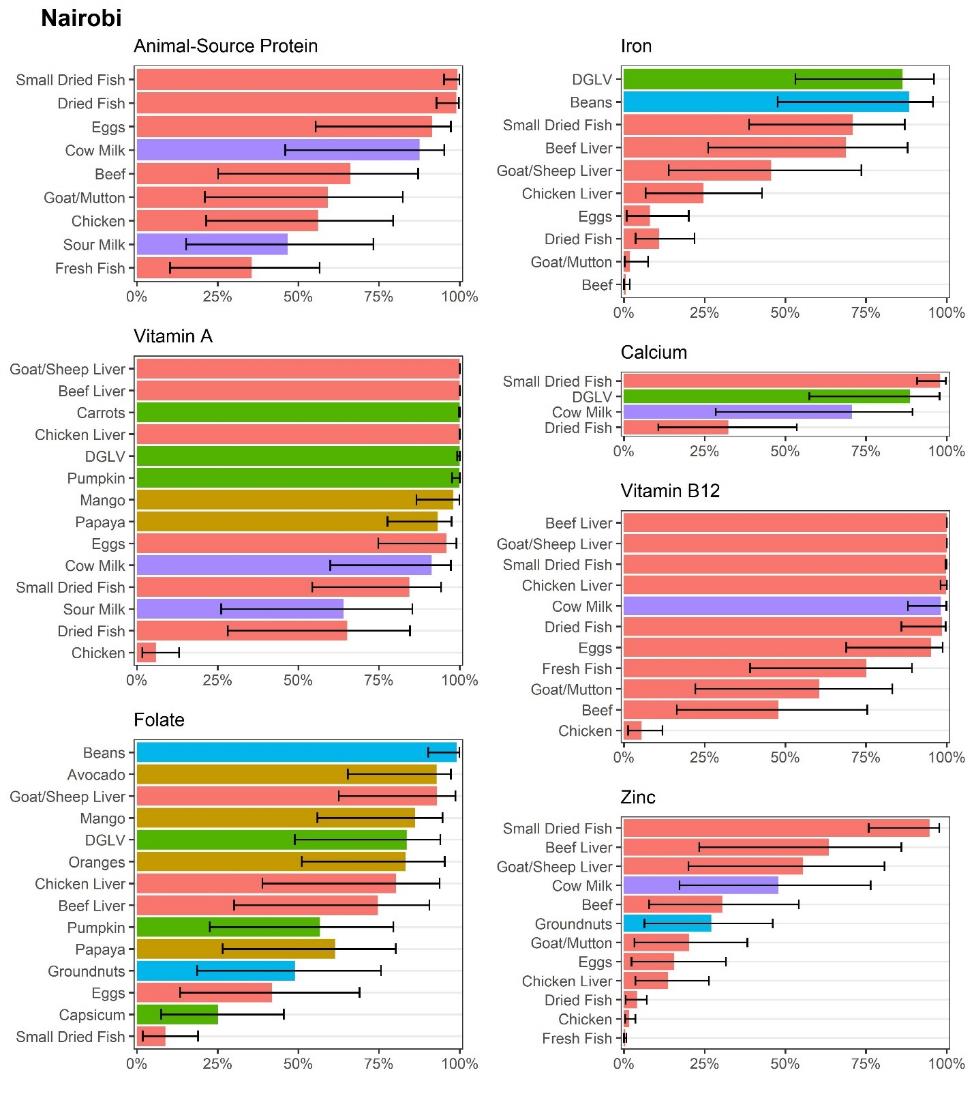


Figure shows the percentage of households that can afford each single food and nutrient at the base affordability thresholds (filled bars; 7.6%), lower thresholds (lower error bars; half the base thresholds, requiring that foods cost less to be considered affordable), and higher thresholds (upper error bars; 50% higher than the base thresholds, allowing foods to cost more and still be considered affordable).


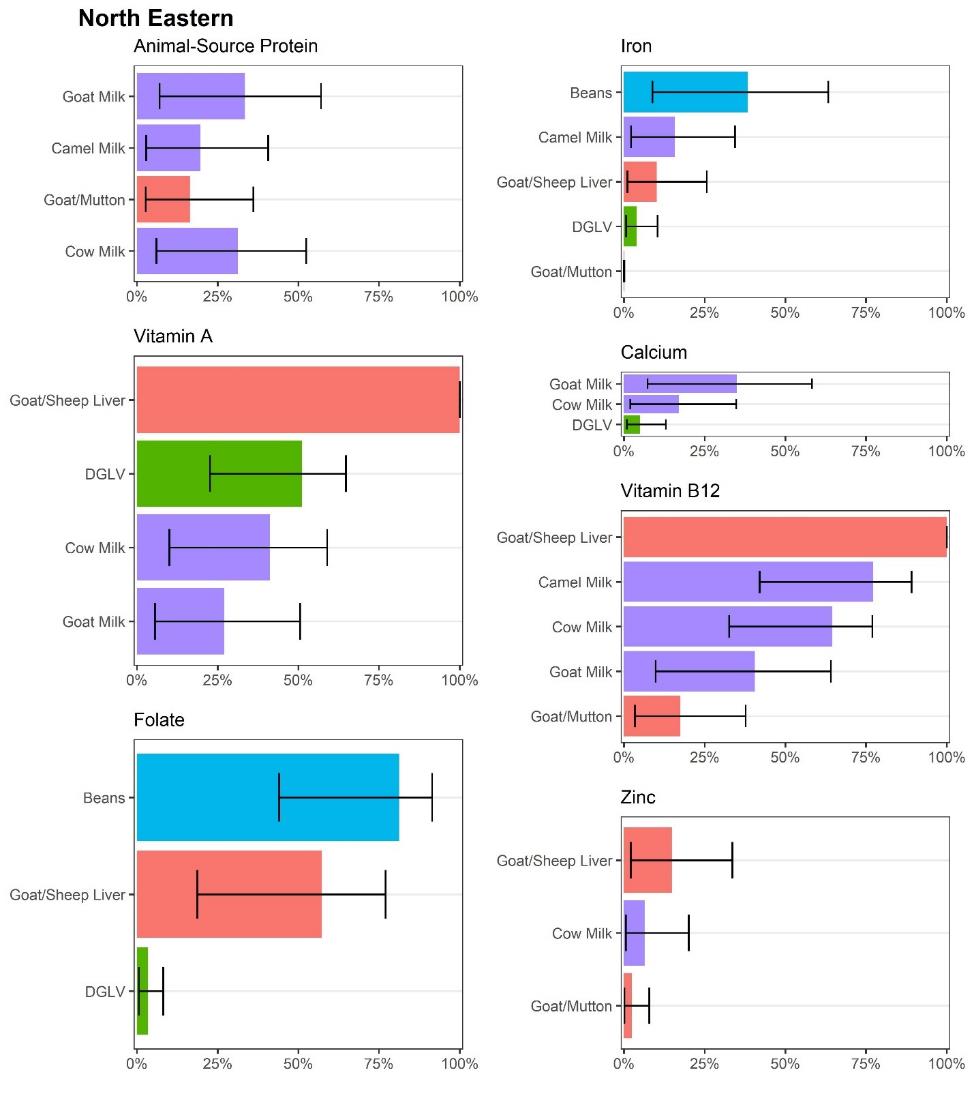


Figure shows the percentage of households that can afford each single food and nutrient at the base affordability thresholds (filled bars; 7.6%), lower thresholds (lower error bars; half the base thresholds, requiring that foods cost less to be considered affordable), and higher thresholds (upper error bars; 50% higher than the base thresholds, allowing foods to cost more and still be considered affordable).


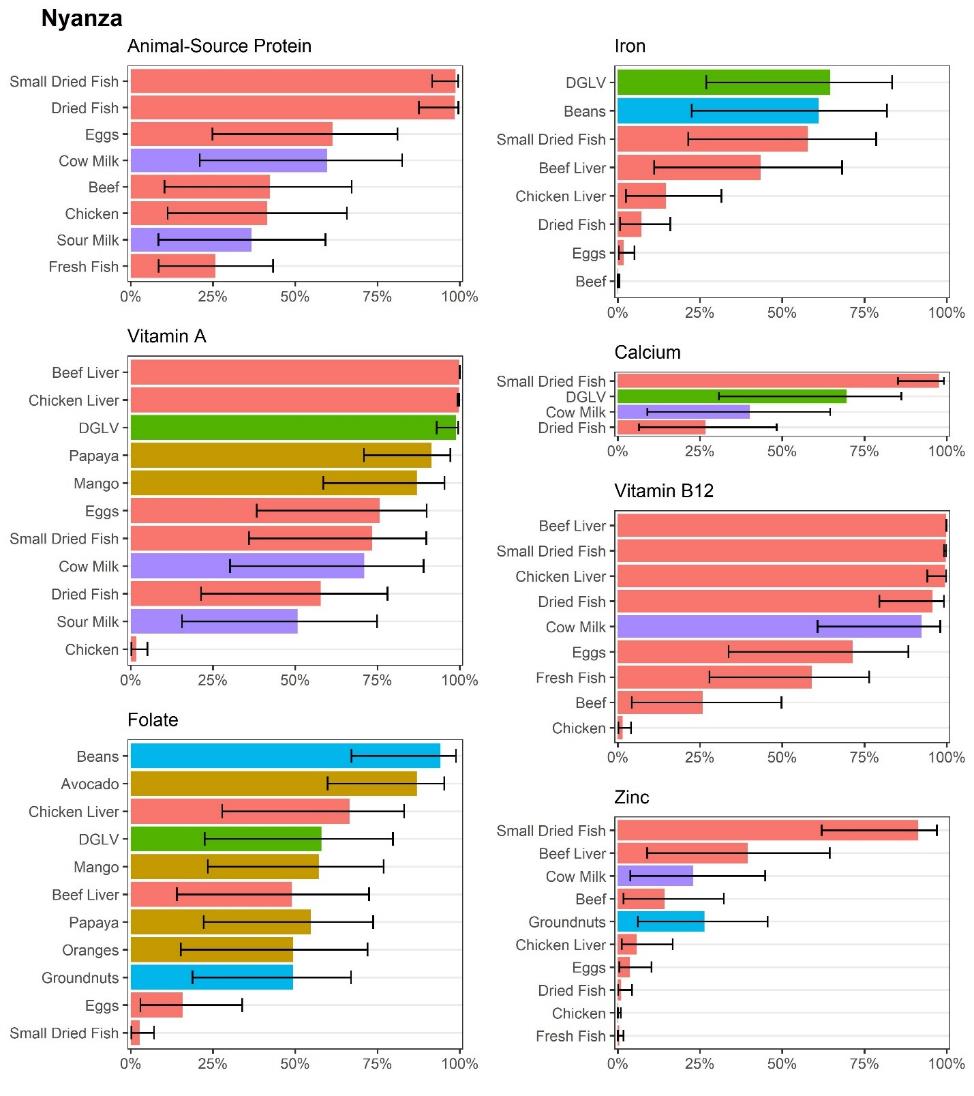


Figure shows the percentage of households that can afford each single food and nutrient at the base affordability thresholds (filled bars; 7.6%), lower thresholds (lower error bars; half the base thresholds, requiring that foods cost less to be considered affordable), and higher thresholds (upper error bars; 50% higher than the base thresholds, allowing foods to cost more and still be considered affordable).


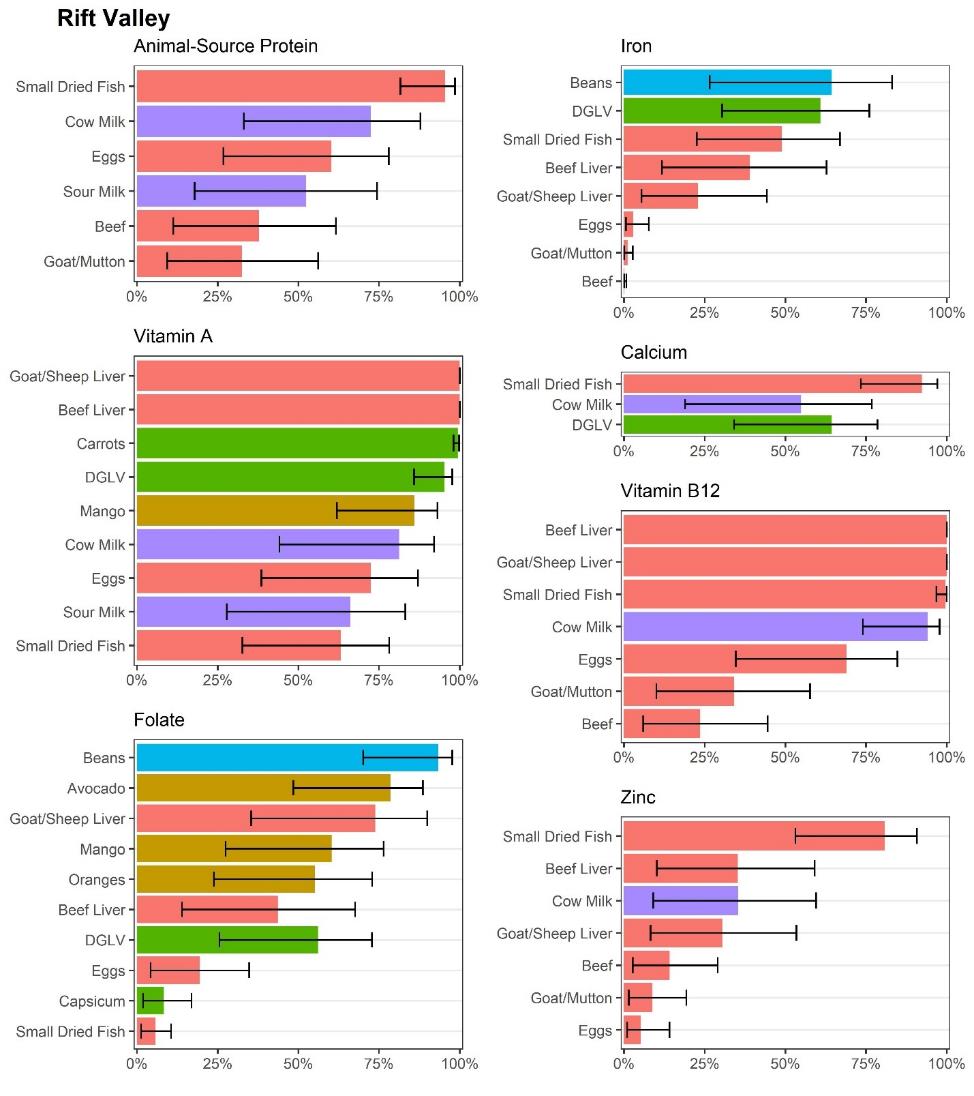


Figure shows the percentage of households that can afford each single food and nutrient at the base affordability thresholds (filled bars; 7.6%), lower thresholds (lower error bars; half the base thresholds, requiring that foods cost less to be considered affordable), and higher thresholds (upper error bars; 50% higher than the base thresholds, allowing foods to cost more and still be considered affordable).


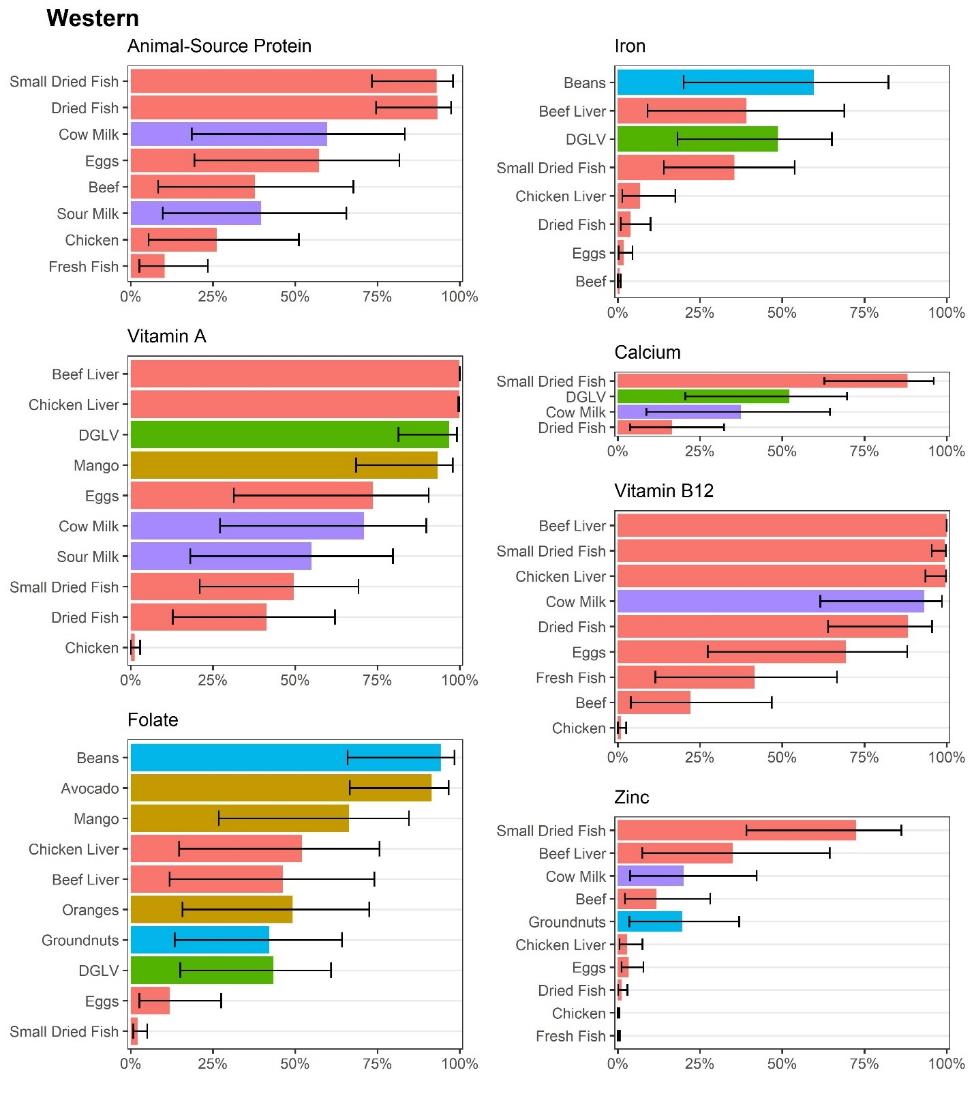


Figure shows the percentage of households that can afford each single food and nutrient at the base affordability thresholds (filled bars; 7.6%), lower thresholds (lower error bars; half the base thresholds, requiring that foods cost less to be considered affordable), and higher thresholds (upper error bars; 50% higher than the base thresholds, allowing foods to cost more and still be considered affordable).

**Appendix Figure 9: Net cost of food portions that could meet half of single nutrient requirements, as a share of adjusted household food expenditures, nationally**


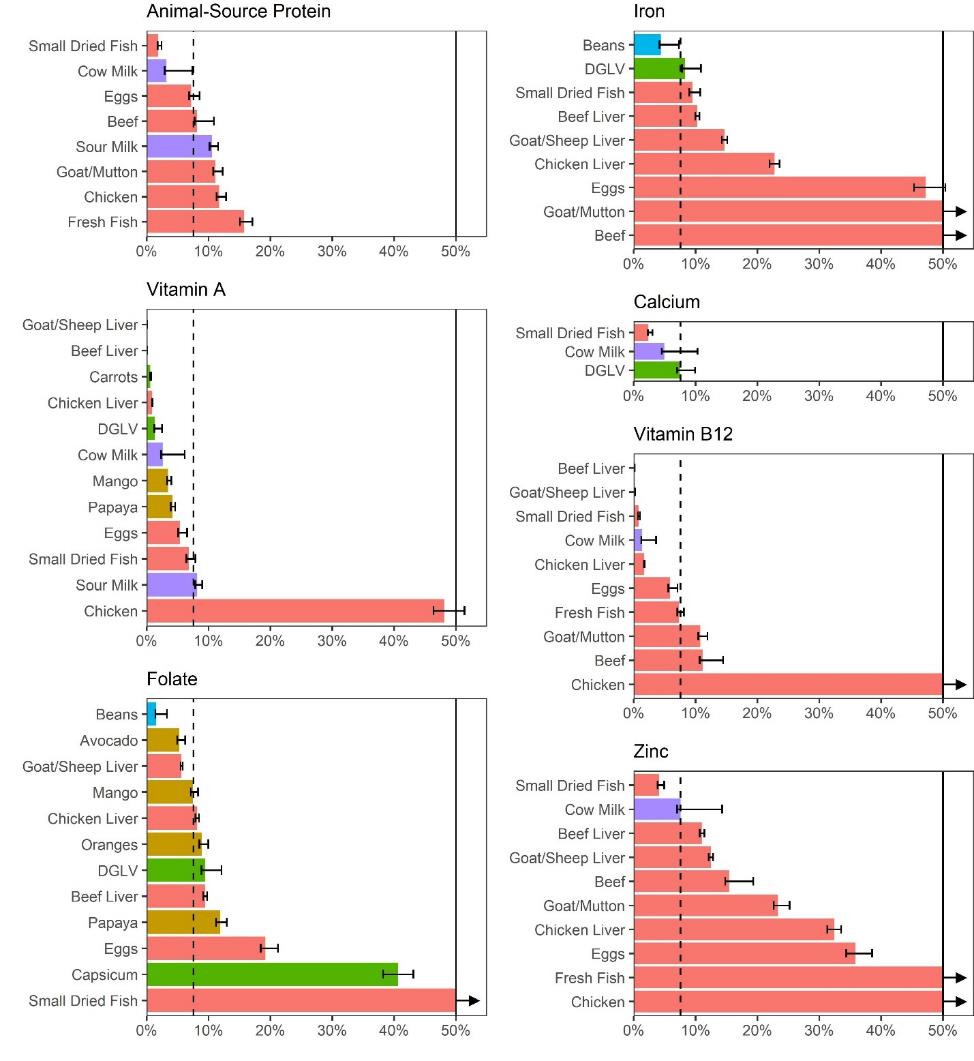


This analysis aims to adjust for current consumption of a food by subtracting current adjusted expenditures on each food from the portion sizes required to meet half of nutrient requirements. Bars were truncated at 50%; arrows indicate that the true values extend beyond 50%. The dashed lines indicate the adjusted single nutrient affordability threshold of 7.6%; bars below that threshold are considered affordable for the average household. Error bars indicate 95% CIs on the means.

**Appendix Figure 10: Net cost of food portions that could meet half of single nutrient requirements, as a share of adjusted household food expenditures, by province**


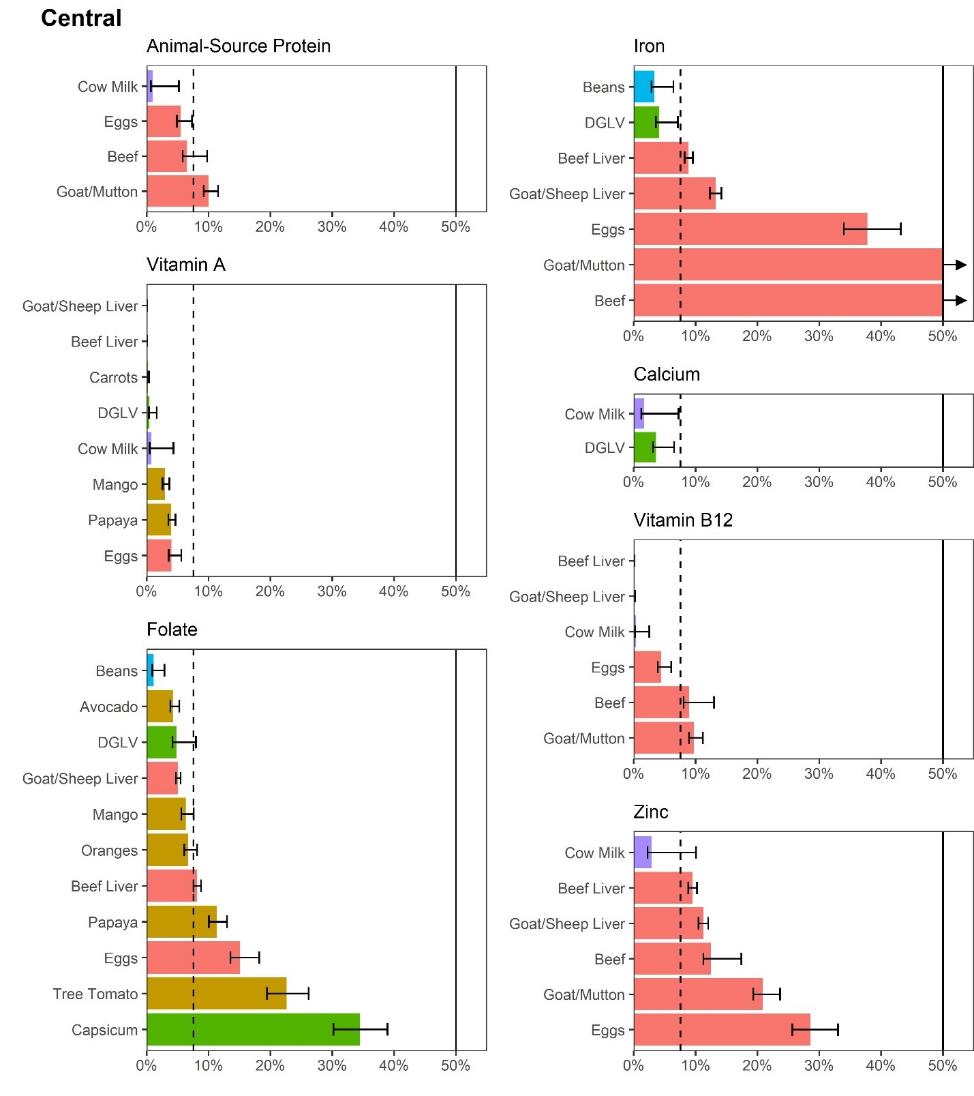


This analysis aims to adjust for current consumption of a food by subtracting current adjusted expenditures on each food from the portion sizes required to meet half of nutrient requirements. Bars were truncated at 50%; arrows indicate that the true values extend beyond 50%. The dashed lines indicate the adjusted single nutrient affordability threshold of 7.6%; bars below that threshold are considered affordable for the average household. Error bars indicate 95% CIs on the means.


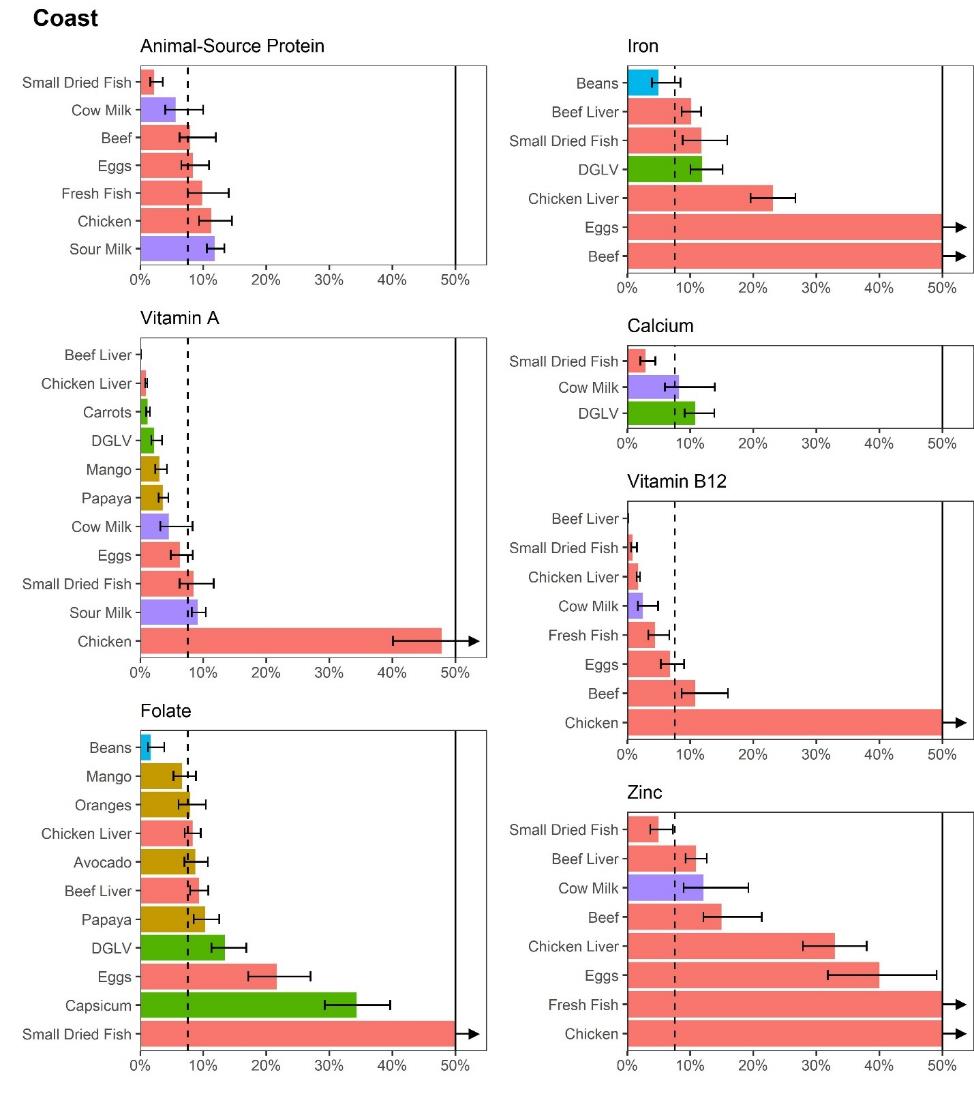


This analysis aims to adjust for current consumption of a food by subtracting current adjusted expenditures on each food from the portion sizes required to meet half of nutrient requirements. Bars were truncated at 50%; arrows indicate that the true values extend beyond 50%. The dashed lines indicate the adjusted single nutrient affordability threshold of 7.6%; bars below that threshold are considered affordable for the average household. Error bars indicate 95% CIs on the means.


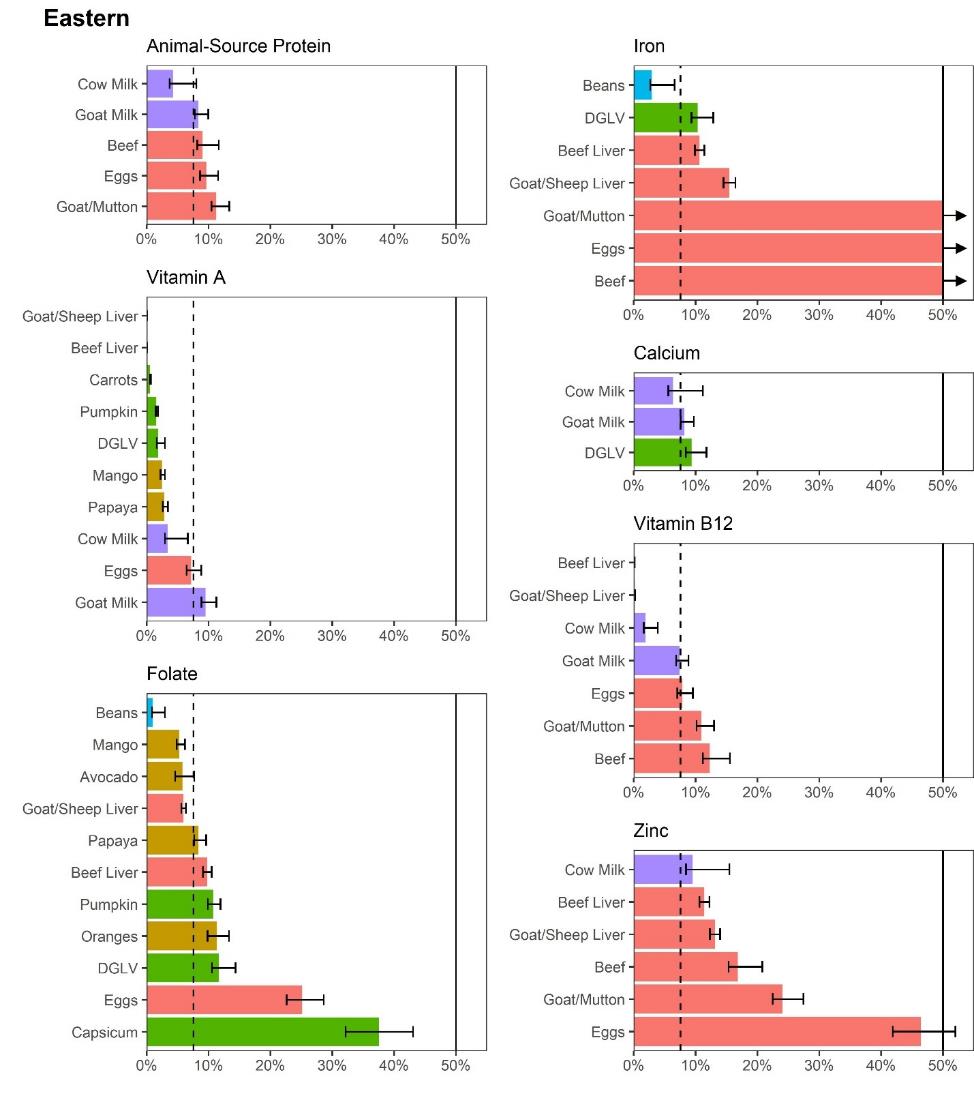


This analysis aims to adjust for current consumption of a food by subtracting current adjusted expenditures on each food from the portion sizes required to meet half of nutrient requirements. Bars were truncated at 50%; arrows indicate that the true values extend beyond 50%. The dashed lines indicate the adjusted single nutrient affordability threshold of 7.6%; bars below that threshold are considered affordable for the average household. Error bars indicate 95% CIs on the means.


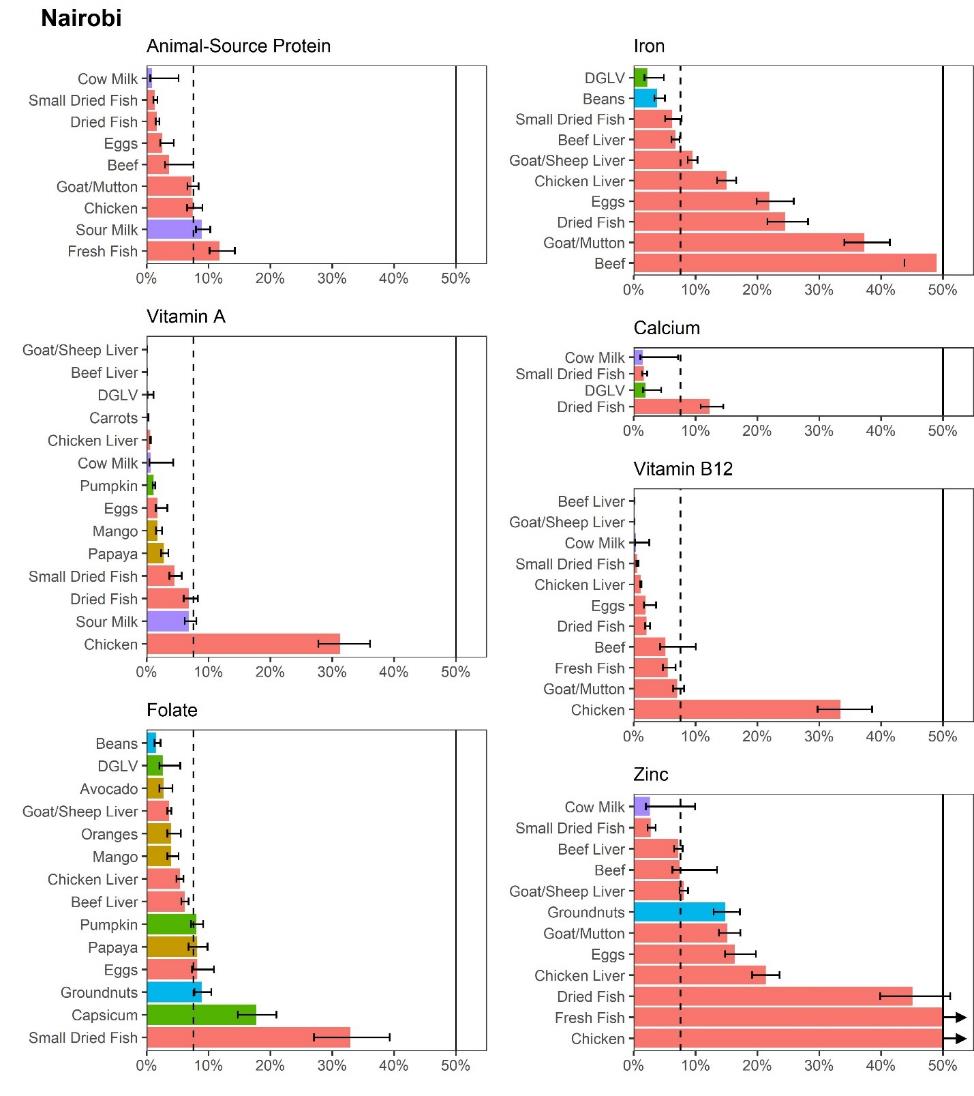


This analysis aims to adjust for current consumption of a food by subtracting current adjusted expenditures on each food from the portion sizes required to meet half of nutrient requirements. Bars were truncated at 50%; arrows indicate that the true values extend beyond 50%. The dashed lines indicate the adjusted single nutrient affordability threshold of 7.6%; bars below that threshold are considered affordable for the average household. Error bars indicate 95% CIs on the means.


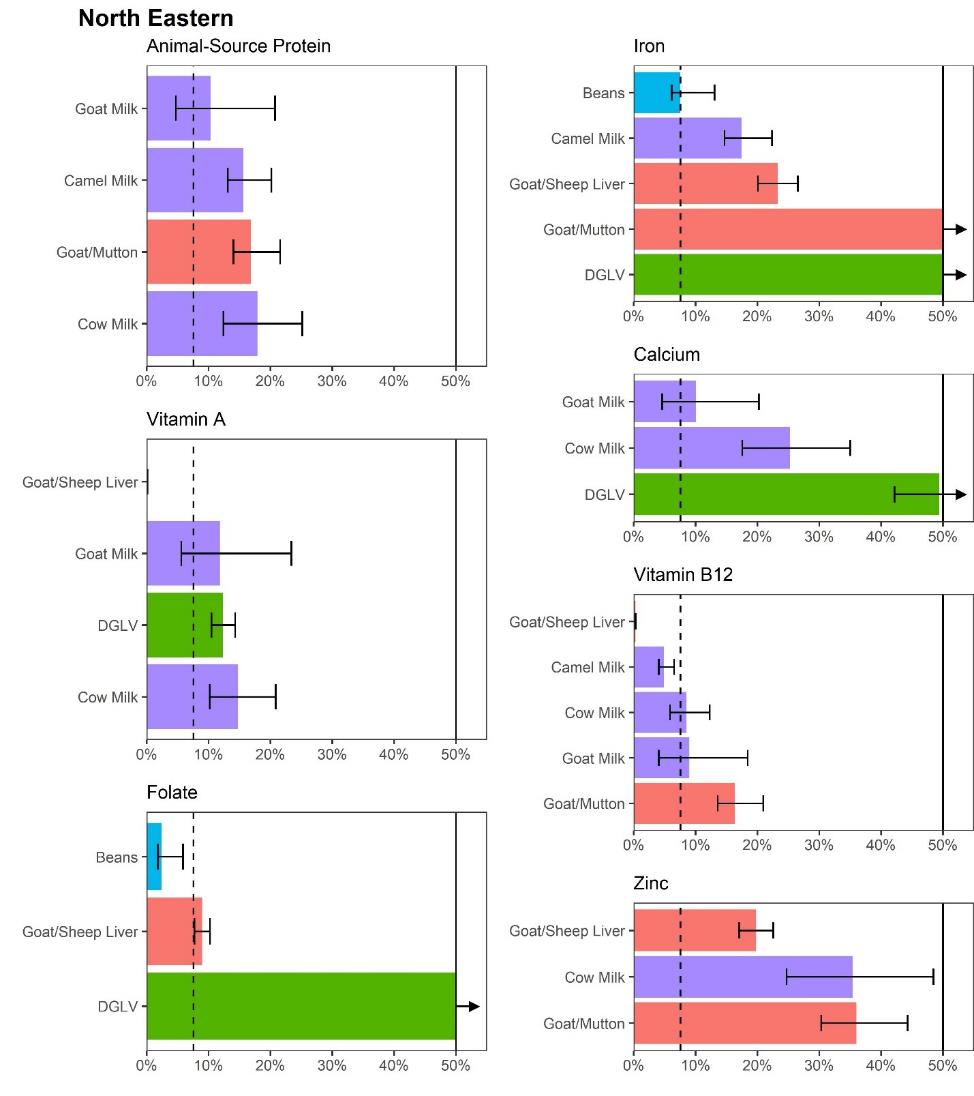


This analysis aims to adjust for current consumption of a food by subtracting current adjusted expenditures on each food from the portion sizes required to meet half of nutrient requirements. Bars were truncated at 50%; arrows indicate that the true values extend beyond 50%. The dashed lines indicate the adjusted single nutrient affordability threshold of 7.6%; bars below that threshold are considered affordable for the average household. Error bars indicate 95% CIs on the means.


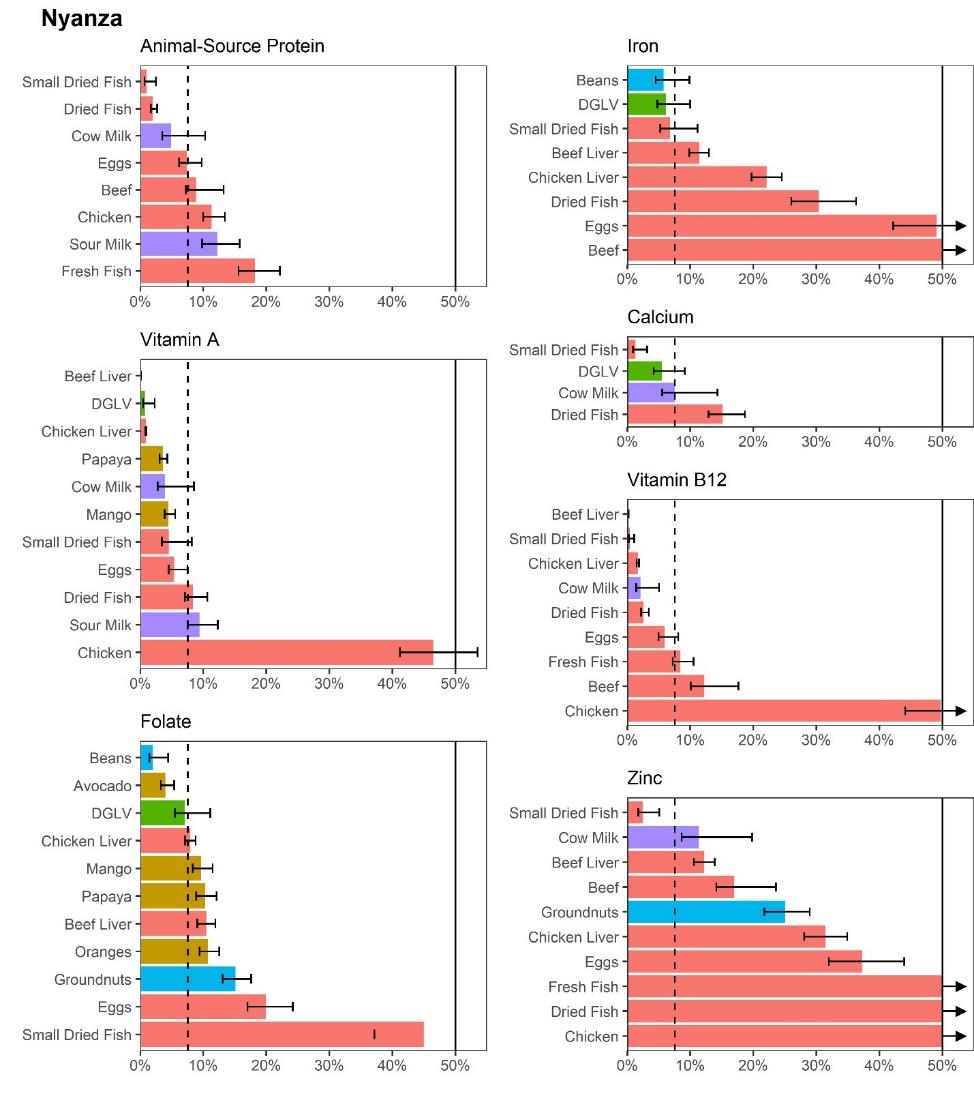


This analysis aims to adjust for current consumption of a food by subtracting current adjusted expenditures on each food from the portion sizes required to meet half of nutrient requirements. Bars were truncated at 50%; arrows indicate that the true values extend beyond 50%. The dashed lines indicate the adjusted single nutrient affordability threshold of 7.6%; bars below that threshold are considered affordable for the average household. Error bars indicate 95% CIs on the means.


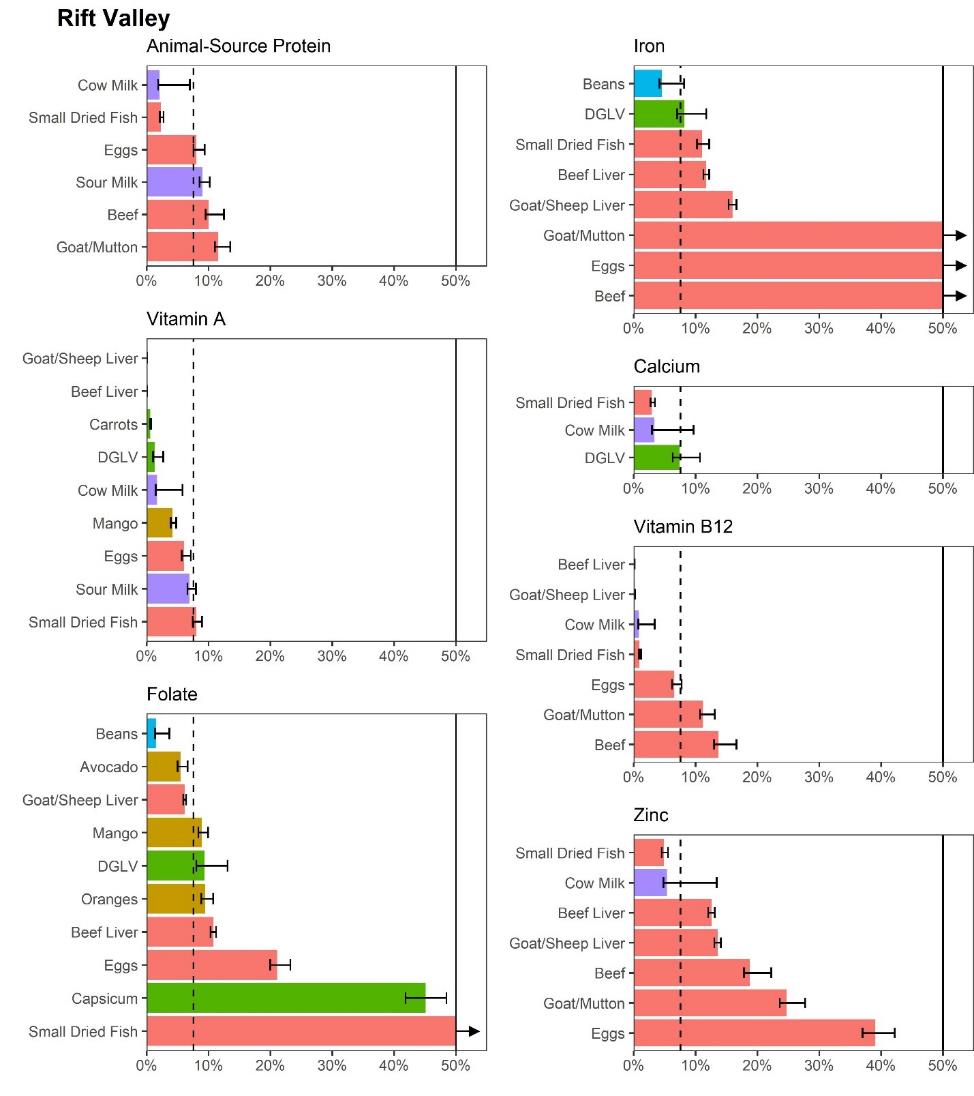


This analysis aims to adjust for current consumption of a food by subtracting current adjusted expenditures on each food from the portion sizes required to meet half of nutrient requirements. Bars were truncated at 50%; arrows indicate that the true values extend beyond 50%. The dashed lines indicate the adjusted single nutrient affordability threshold of 7.6%; bars below that threshold are considered affordable for the average household. Error bars indicate 95% CIs on the means.


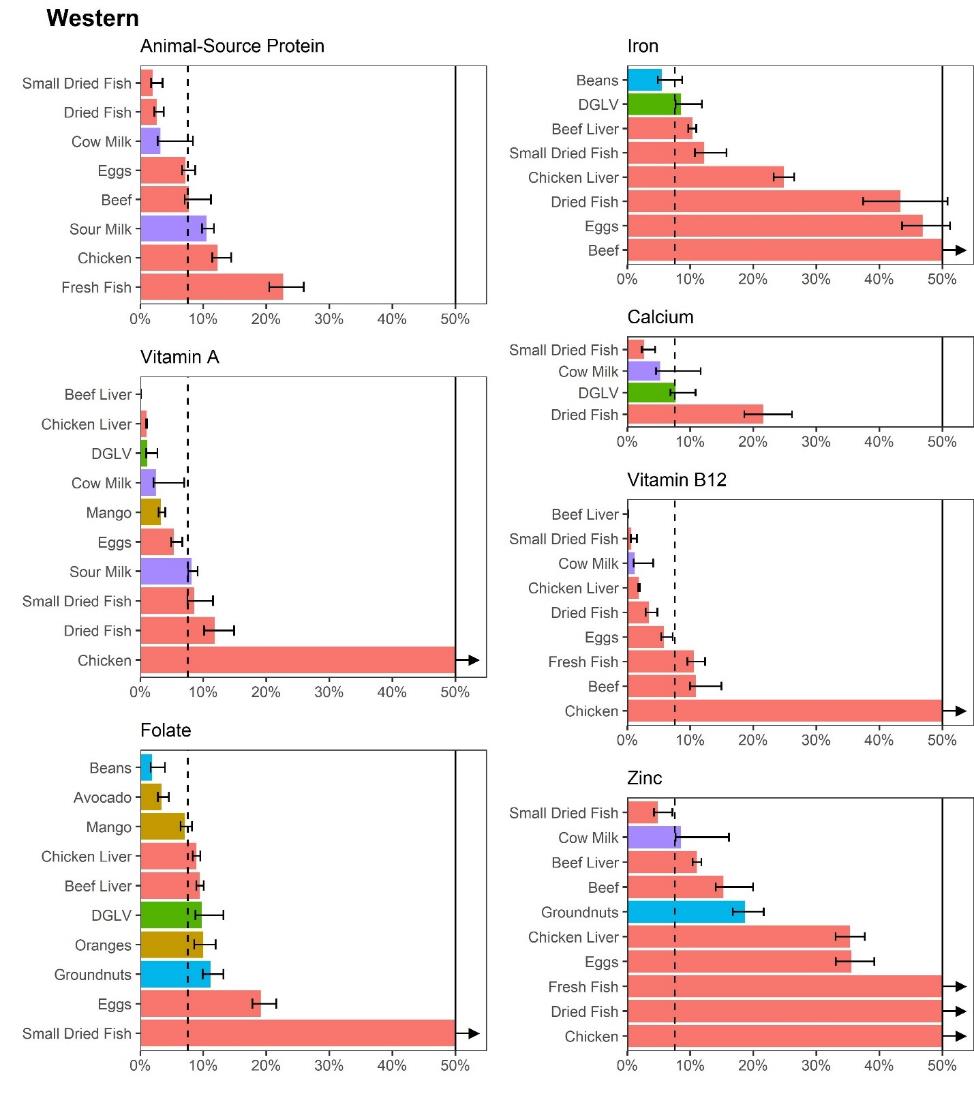


This analysis aims to adjust for current consumption of a food by subtracting current adjusted expenditures on each food from the portion sizes required to meet half of nutrient requirements. Bars were truncated at 50%; arrows indicate that the true values extend beyond 50%. The dashed lines indicate the adjusted single nutrient affordability threshold of 7.6%; bars below that threshold are considered affordable for the average household. Error bars indicate 95% CIs on the means.

**Appendix Figure 11: Cost of foods to meet half of single nutrient requirements as a share of adjusted household food expenditures, with sensitivity analysis on nutrient densities for select foods**

**
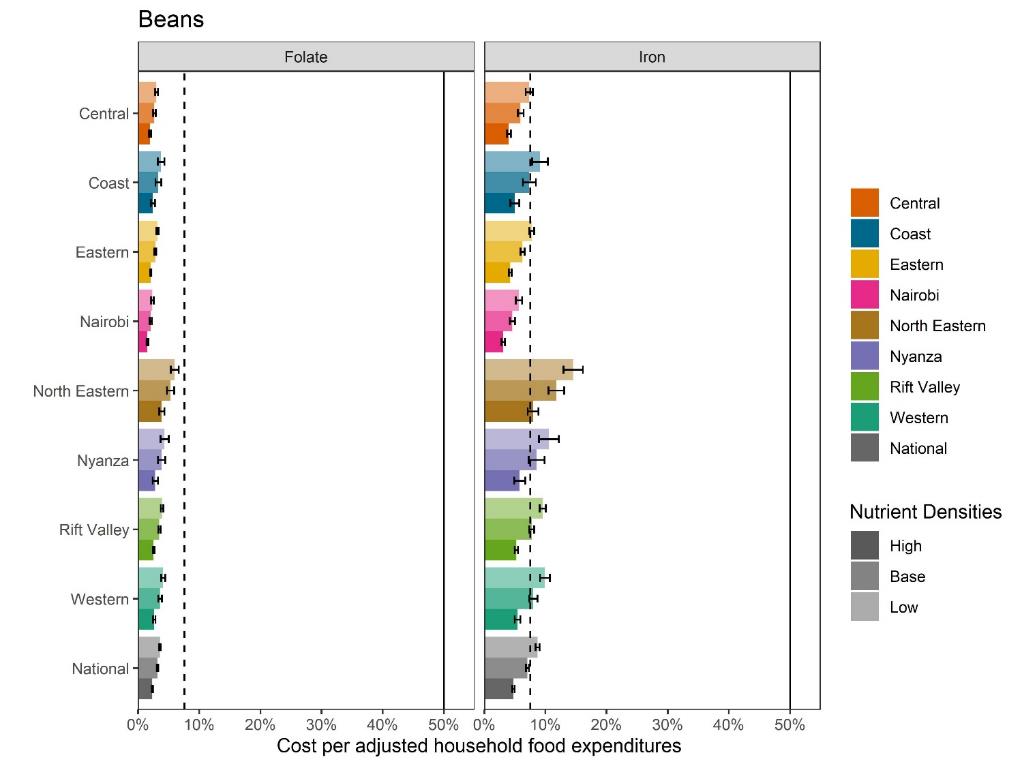
**

Figure shows affordability when the nutrient density of beans was varied from the median (base, used in main analysis) to the 10^th^ (low) and 90^th^ (high) percentiles over several varieties of beans from the Kenya food composition table. Bars were truncated at 50%; arrows indicate that the true values extend beyond 50%. The dashed lines indicate the adjusted single nutrient affordability threshold of 7.6%; bars below that threshold are considered affordable for the average household. Error bars indicate 95% CIs on the means.

**
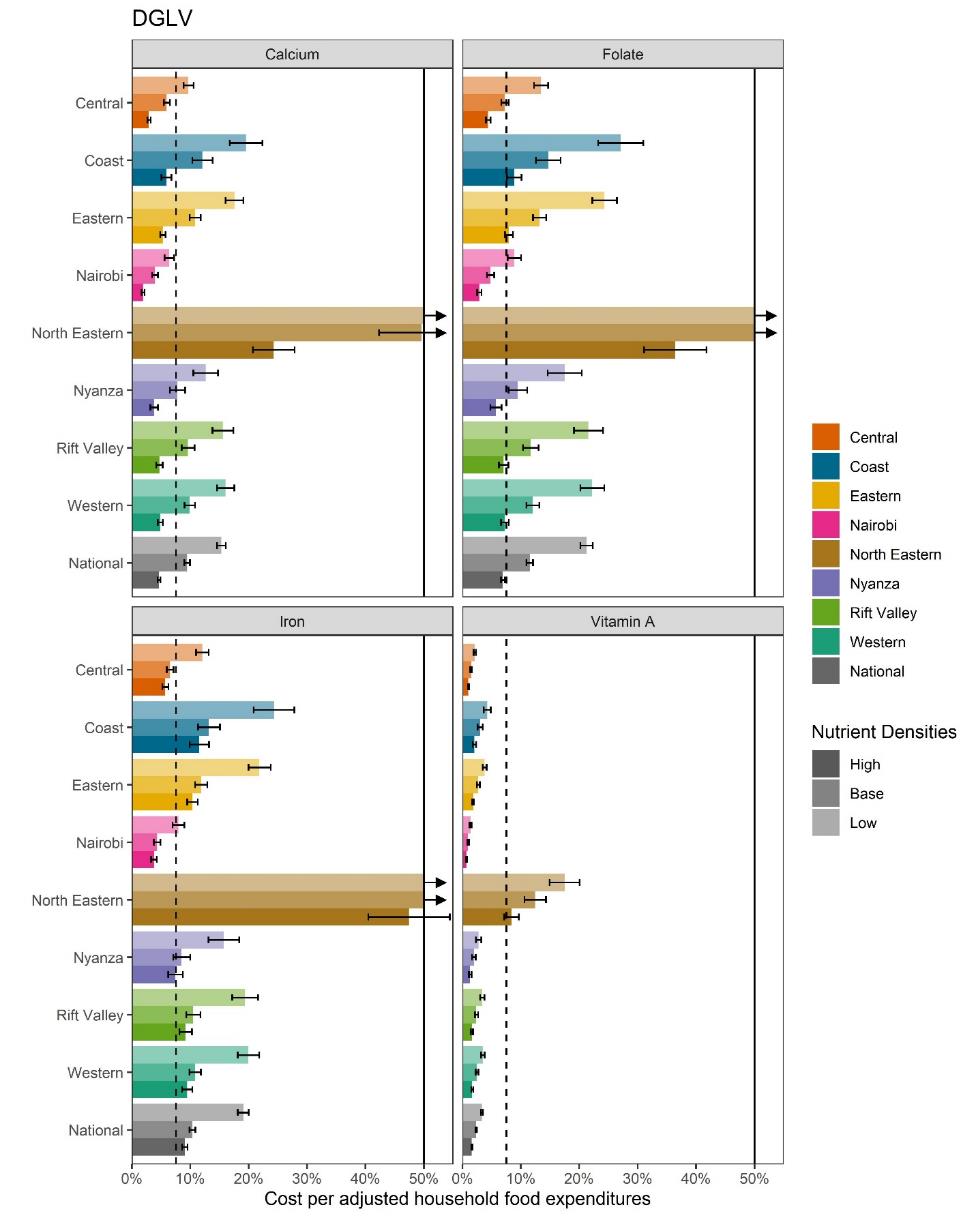
**

Figure shows affordability when the nutrient density of dark green leafy vegetables was varied from the median (base, used in main analysis) to the 10^th^ (low) and 90^th^ (high) percentiles over several varieties of DGLV from different food composition tables (see Appendix Table 2). Bars were truncated at 50%; arrows indicate that the true values extend beyond 50%. The dashed lines indicate the adjusted single nutrient affordability threshold of 7.6%; bars below that threshold are considered affordable for the average household. Error bars indicate 95% CIs on the means.

**
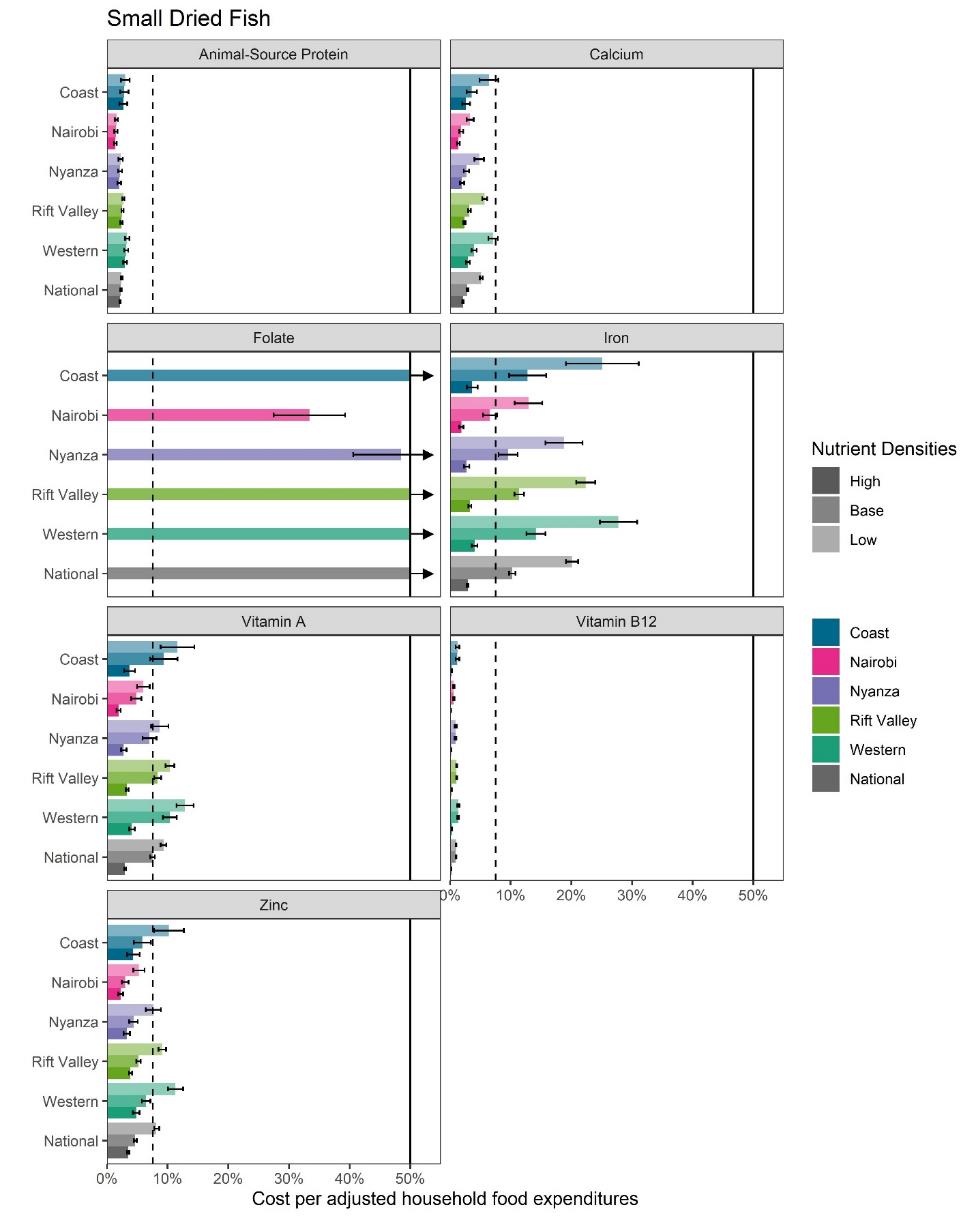
**

Figure shows affordability when the nutrient density of small dried fish was varied from the median (base, used in main analysis) to the 10^th^ (low) and 90^th^ (high) percentiles over several varieties and values from different food composition tables (see Appendix Table 2). Bars were truncated at 50%; arrows indicate that the true values extend beyond 50%. The dashed lines indicate the adjusted single nutrient affordability threshold of 7.6%; bars below that threshold are considered affordable for the average household. Error bars indicate 95% CIs on the means.

**
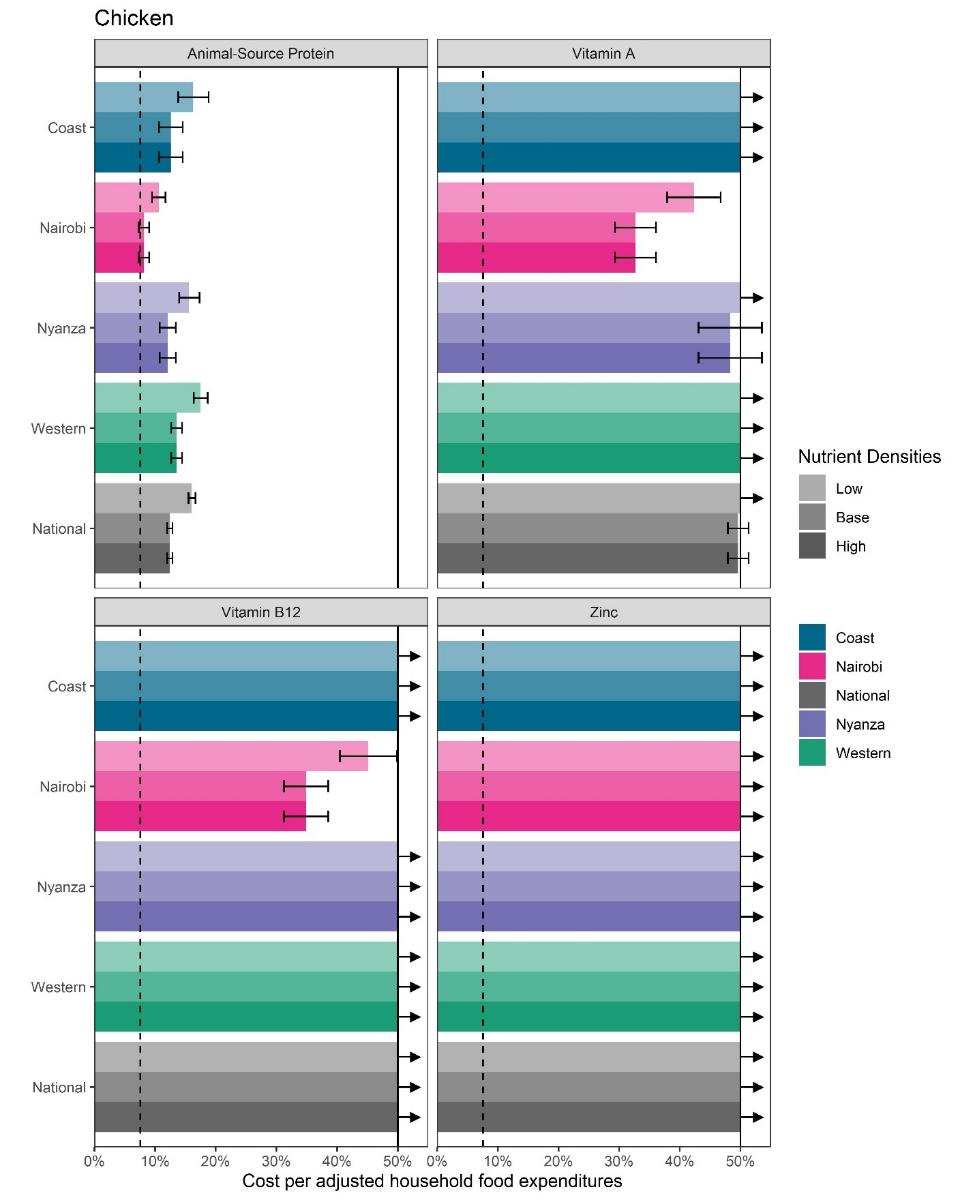
**

The nutrient density of a purchased chicken depends on whether that chicken is purchased live or dead (and presumably partially butchered). In the base analysis, we assumed a 50/50 split of live and dead chickens were purchased. In this figure, high nutrient density corresponds to 100% dead chickens and low nutrient density corresponds to 100% live chickens being purchased for consumption. Bars were truncated at 50%; arrows indicate that the true values extend beyond 50%. The dashed lines indicate the adjusted single nutrient affordability threshold of 7.6%; bars below that threshold are considered affordable for the average household. Error bars indicate 95% CIs on the means.
